# Chronic kidney disease in cats alters response of the plasma metabolome and fecal microbiome to dietary fiber

**DOI:** 10.1371/journal.pone.0235480

**Published:** 2020-07-02

**Authors:** Jean A. Hall, Matthew I. Jackson, Dennis E. Jewell, Eden Ephraim

**Affiliations:** 1 Department of Biomedical Sciences, College of Veterinary Medicine, Oregon State University, Corvallis, Oregon, United States of America; 2 Pet Nutrition Center, Hill’s Pet Nutrition, Topeka, Kansas, United States of America; 3 Department of Grain Science and Industry, Kansas State University, Manhattan, Kansas, United States of America; University of Illinois, UNITED STATES

## Abstract

The objectives were to determine the effects of feeding different fiber types to healthy cats and cats with chronic kidney disease (CKD) on plasma metabolites and the fecal microbiome.

**Methods:**

A cross-over study within a split-plot design was performed using healthy (n = 10) and CKD cats [IRIS Stage 1 and 2; n = 10]. Cats were fed dry Prescription Diet^®^ k/d^®^ Feline with chicken, during a pre-trial period and then randomly assigned to two fiber treatments for 4 weeks each. Treatment foods were formulated similar to pre-trial food, with the exception that they contained 0.500% betaine, 0.586% oat beta glucan, and either 0.407% short chain fructooligosaccharides (scFOS) fiber or 3.44% apple pomace. Both foods had similar crude fiber percent (2.0 and 2.1% for scFOS and apple pomace, respectively) whereas soluble fiber was 0.8 and 1.6%, respectively.

**Results:**

Plasma metabolites separated cats based on health status. At baseline, cats with CKD had significantly higher circulating concentrations of creatinine, urea, and some microbial and host tryptophan metabolites including several indole sulfates and kynurenate. Healthy cats had higher concentrations of the antioxidant α-tocopherol after consuming apple pomace; alternatively, they had higher concentrations of inflammatory sphingolipid metabolites after consuming scFOS, but not after consuming apple pomace. The CKD cats had higher concentrations of the more oxidized glutathione metabolites after consuming apple pomace compared with scFOS, as well as higher concentrations of inflammatory sphingolipid metabolites after consuming apple pomace, but not scFOS. After consuming scFOS, CKD cats had lower concentrations of the phenolic uremic toxins guaiacol sulfate and 4-vinylphenol sulfate compared with after consuming apple pomace. At baseline, there were five significant microbiota OTU differences in CKD cats compared with healthy cats. Overall, the OTUs in CKD cats were more resistant to change after feeding either fiber source. Counts of an unclassified genus in the family S24-7 in the order *Bacteroidales* (OTU 100296), were lower in CKD cats compared with healthy cats at baseline (*P* = 0.001), but increased after consumption of food containing scFOS (*P* = 0.006). Linear regression analysis showed that this genus had significant negative correlations with several microbial uremic toxins. None of the baseline differences in OTUs between healthy and CKD cats changed after CKD cats consumed food containing apple pomace.

**Conclusions:**

Health status impacts the influence of dietary fermentable fibers on the feline plasma metabolome and fecal microbiome. A more readily fermented fiber such as scFOS is preferable to apple pomace as a fiber source for cats with CKD.

## Introduction

In cats, chronic kidney disease (CKD) is a major cause of morbidity, especially in senior-adult and geriatric cats [1–4]. Cats with CKD experience similar manifestations as humans, in that both have progressive loss of renal function, which may eventually lead to uremic crises and death. In humans, dietary contents and their metabolites are closely related to CKD progression [5]. In cats, feeding a renal protective food with International Renal Interest Society (IRIS) stage 2 CKD or higher is considered the standard of care with strong evidence supporting this recommendation [6, 7]. We recently reported that cats with IRIS stage 1 and 2 CKD maintained body weight and lean muscle mass when fed food having increased caloric density, and enhanced concentrations of carnitine and essential amino acids [8]. Thus, feeding a renal-support diet to cats with earlier stages of CKD also slows disease progression.

Many factors can alter the biochemical milieu of the gastrointestinal track in patients with CKD [5, 9]. An abnormal microbiome composition has been noted in patients with CKD, and may result from dietary restrictions, as well as prescribed drugs, accumulations of toxins (urea, uric acid, oxalates, and other uremic toxins), and an impaired immune response [9, 10]. In humans, fruits and vegetables are a major source of dietary fiber; although consumption may be limited to prevent hyperkalemia [9]. Dietary fiber is degraded by colonic bacteria to generate short-chain fatty acids, which are a major source of energy for colonocytes [9].

We hypothesized that the type of fermentable fiber fed to cats with CKD might influence the fecal microbiota. Pathogenic links between the intestinal microbiota and CKD progression have been described in humans [10], including increased gut permeability, and excessive production of microbiota generated nephrotoxins such as p-cresyl sulfate, p-cresyl-glucuronide, 3-hydroxyindole sulfate (3-indoxylsulfate), indole-3 acetic acid, and trimethylamine N-oxide [11]. Additionally, in cats with CKD, decreased fecal microbiome diversity and richness have been reported [12]. Concentrations of 3-indoxylsulfate were significantly increased in cats with CKD [12]. Thus, we hypothesized that the source of dietary fiber fed might alter circulating concentrations of uremic toxins in cats with CKD.

We have previously shown that feeding a combination of betaine and prebiotics (including oat beta glucan) compared with feeding control food resulted in CKD cats having higher body mass [13]. Thus, betaine (0.500%) and oat beta glucan (0.586%) were included in both test foods in this study. Betaine is known to have osmoprotective effects on the kidney [14]. It also decreases concentrations of homocysteine by serving as a methyl donor [15]. In addition, betaine acts as a chaperone to stabilize protein structure under denaturing conditions [16]. Oat beta glucan is a natural soluble fiber. It is a viscous polysaccharide made up of repeating glucose subunits with bonds that are beta 1–3 and beta 1–4 linkages. The beta 1–3 linkages make it soluble. In comparison, the indigestible fiber cellulose is also a beta glucan, but it is insoluble because it has only beta 1–4 linkages.

The objective of this study was to evaluate the effects of feeding betaine, oat beta glucan, and either short chain fructooligosaccharides (scFOS) or apple pomace as additional fiber sources to healthy cats and cats with CKD (IRIS stages 1 and 2). We assessed circulating metabolite concentrations, and in particular, markers of inflammation (e.g., sphingolipid metabolites [17, 18]) and oxidative stress (e.g., those involved in tocopherol and glutathione metabolism), as well as the gastrointestinal microbial community found in fecal specimens.

## Methods

All study protocols and this study were reviewed and approved by the Institutional Animal Care and Use Committee, Hill’s Pet Nutrition, Inc., Topeka, KS, USA (Permit Number: CP709), and complied with the National Institutes of Health Guide for the Care and Use of Laboratory Animals [19]. Cats were housed in groups and allowed access to indoor runs. Cats also had exposure to natural light that varied with seasonal changes. All cats were provided with regular opportunities to exercise, with access to toys. Cats were owned by the commercial funders of this research or their affiliates, who gave permission for them to be included in this study. At the conclusion of the study, all cats were returned to the Hill’s Pet Nutrition, Inc. colony.

### Participants and study design

This was a cross-over study within a split-plot design performed using healthy cats (n = 10) and CKD cats (IRIS stage 1 and 2; n = 10). The study used a pre-trial food, Food A and Food B. The pre-trial food was Prescription Diet^®^ k/d^®^ Feline with chicken, dry. Food A was the pre-trial food supplemented with betaine (0.500%), oat beta glucan (0.586%), and 0.407% scFOS. Food B was the pre-trial food supplemented with betaine (0.500%), oat beta glucan (0.586%), and 3.44% apple pomace. Cats were fed pre-trial food for 14-days and then were randomly assigned to Food A or Food B. Cats fed Food A for 4 weeks were then switched to Food B for 4 weeks. Cats fed Food B for 4 weeks were then switched to Food A for 4 weeks. All cats had access to electronic feeders whereby fresh food was offered daily with amounts available for consumption calculated to maintain body weight; water was available *ad libitum*. Actual daily food intake (g/day) was recorded for each cat.

All cats were of domestic shorthair breed. Demographic data is shown in Table 1. Cats in this study ranged in age from 4.8 to 9.3 years. Inclusion criteria were healthy cats or cats with CKD. Cats were determined to be healthy based on the results of an annual physical examination, complete blood count (CBC), serum biochemistries, and urinalysis. Cats were excluded from the healthy group if they were known to have problems eating new foods or problems with repeated blood sampling, and/or had any diagnosed disease condition such as inflammatory bowel disease, dermatitis, food allergy, cancer/tumor, liver disease, CKD, or chronic urinary tract infection. The criterion for removal from the study was development of any condition whereby removal would benefit the animal, including any cat refusing to eat, or inadequate food intake resulting in weight loss greater than 15% of body weight. No healthy cats were removed from the study.

**Table 1:** Demographic data, mean (standard deviation), at baseline for healthy adult cats (n = 10) and cats with IRIS stage 1 and 2 chronic kidney disease (CKD; n = 10).

Cats	Healthy	CKD
Age, years	7.2 (1.3)	8.0 (1.8)
Sex	8 SF, 2 NM	5 SF, 5 NM
Body weight, kg	4.55 (0.68)	5.40 (0.85)

SF: spayed female; NM: neutered male

Cats with CKD were determined to have IRIS stage 1 or 2 CKD based on the results of an annual physical examination, CBC, serum biochemistries, and urinalysis. Cats with CKD included cats that were persistently azotemic with creatinine (Cr) 1.6 to 3.2 mg/dL over an extended period, typically for ≥ 3 months (n = 5); four of these cats had nephrolithiasis. Nonazotemic cats with kidney stones (n = 4) as well as one cat with abnormal kidneys found on physical examination and ultrasonographic imaging (n = 1; right kidney missing) were also included in the CKD group. Cats with any other abnormal clinical findings except CKD, IRIS stage 1 or 2, were excluded from the CKD group, notwithstanding history and physical examination findings (changes in urine volume or changes in kidney size or shape) that were consistent with CKD. The criterion for removal of CKD cats from the study was the same as for healthy cats. No CKD cats were removed from the study.

Blood and fecal samples were collected at baseline (end of 14-day pre-trial period) and at the end of each 4-week feeding period to evaluate changes in plasma metabolites and fecal microbiome. Additional assays included CBC, serum biochemical analysis, urinalysis, and urine protein/creatinine ratio. Body weights were recorded weekly.

### Foods

Prescription Diet^®^ k/d^®^ Feline with chicken, dry, was fed during a pre-trial period for 14 days (Table 2). Foods A and B were formulated similar to the food fed in the pre-trial-period, with the exception that both were supplemented with betaine (0.500%) and oat beta glucan (0.586%). In addition, Food A was supplemented with 0.407% scFOS fiber and Food B was supplemented with 3.44% apple pomace (Table 2). Oat beta glucan was 22% beta glucan (0.129%); scFOS was 95% scFOS (0.387%) and thus, 3× higher concentration than oat beta glucan. Apple pomace contained 43.2% total dietary fiber, 20.8 mg/g free polyphenol and 27.34 mg/g bound polyphenols. All cat foods were prepared by Hill’s Pet Nutrition, Inc., and met the nutritional requirements for adult cats (≥ 1 year) as established by the Association of American Feed Control Officials (AAFCO). Food was available in dry form only. Macronutrient composition, and soluble and insoluble fiber concentrations of foods were determined by a commercial laboratory (Eurofins Scientific, Inc., Des Moines, IA). Proximate analyses were completed using the following techniques: moisture—AOAC 930.15; protein—AOAC 2001.11; fat—AOAC 954.02; fiber—AOAC 962.09; ash—AOAC 942.0; and soluble and insoluble fiber AOAC 991.43. Carbohydrate composition was determined by calculation. Food composition, expressed as percentage of food, as fed, is shown in Table 2. Vitamin, mineral, and fatty acid analyses were performed by the same commercial laboratory. Fatty acid (FA) concentrations were determined by gas chromatography of FA methyl esters, and were expressed as g/100 g of FAs as fed. The sum of dietary saturated FA (SFA) was determined as follows: 8:0+10:0+11:0+12:0+14:0+15:0+16:0+17:0+18:0+20:0+22:0+24:0. The sum of dietary monounsaturated FA (MUFA) was determined as follows: 14:1+15:1+16:1+17:1+18:1+20:1+22:1+24:1. The sum of dietary polyunsaturated FA (PUFA) was determined as follows: 18:2(n-6)+18:3(n-6)+18:3(n-3)+18:4(n-3)+20:2(n-6)+20:3(n-6)+20:3(n-3)+20:4(n-6)+20:4(n-3)+20:5(n-3)+21:5(n-3)+22:2(n-6)+22:4(n-6)+22:5(n-6)+22:5(n-3)+22:6(n-3).

**Table 2:** Composition of pre-trial food^1^, Food A^2^, and Food B^3^.

Nutrient	Pre-trial Food	Food A with scFOS	Food B with apple pomace
Moisture	5.47	5.34	5.97
Protein	27.6	28.0	27.3
Fat	19.9	19.5	19.5
Atwater Energy,^4^ kcal/kg	4101	4063	4032
Ash	4.50	4.42	4.59
Crude Fiber	1.3	2.0	2.1
Insoluble Fiber	4.0	3.4	5.1
Soluble Fiber	1.3	0.8	1.6
Total Dietary Fiber	5.3	4.2	6.7
Calcium	0.73	0.72	0.79
Phosphorus	0.64	0.51	0.47
Sodium	0.24	0.22	0.22
ARA [20:4 (n-6)]	0.04	0.04	0.04
EPA [20:5 (n-3)]	<0.01	0.02	0.02
DHA [22:6 (n-3)]	0.01	0.03	0.02
SFA^5^	6.32	6.59	6.72
MUFA^6^	7.40	8.04	8.24
PUFA^7^	9.58	7.83	8.25
Total FA	18.56	18.61	19.16
(n-6) FA^8^	4.10	3.70	3.90
(n-3) FA^9^	0.69	0.22	0.23
(n-6):(n-3) ratio	5.9	16.8	17.0

^1^Pre-trial food was Prescription Diet^®^ k/d^®^ Feline with chicken, dry. All analytical values are expressed as percentage of food, as fed, unless otherwise indicated.

^2^ Food A was prepared by Hill’s Pet Nutrition, Inc. and was similar to the pre-trial food, with the exception that it was supplemented with betaine (0.500%), oat beta glucan (0.586%), and 0.407% scFOS.

^3^Food B was prepared by Hill’s Pet Nutrition, Inc. and was similar to the pre-trial food, with the exception that it contained 0.500% betaine, 0.586% oat beta glucan, and 3.44% apple pomace.

^4^ Energy calculated using the modified Atwater factors as described [20].

^5^ Sum of the SFA: 8:0+10:0+11:0+12:0+14:0+15:0+16:0+17:0+18:0+20:0+22:0+24:0.

^6^ Sum of the MUFA: 14:1+15:1+16:1+17:1+18:1+20:1+22:1+24:1.

^7^ Sum of the PUFA: 18:2(n-6)+18:3(n-6)+18:3(n-3)+18:4(n-3)+20:2(n-6)+20:3(n-6)+20:3(n-3)+20:4(n-6)+20:4(n-3)+20:5(n-3)+21:5(n-3)+22:2(n-6)+22:4(n-6)+22:5(n-6)+22:5(n-3)+22:6(n-3).

^8^ Sum of the (n-6) fatty acids.

^9^ Sum of the (n-3) fatty acids.

All three foods contained similar concentrations (within analytical variance of targets) of protein, and had similar predicted caloric content. Foods A and B were otherwise similar with equal crude fiber (2.0 and 2.1% for Food A scFOS and Food B apple pomace, respectively) whereas the soluble fiber was 0.8 and 1.6%, respectively.

### Plasma metabolomics

Analysis of plasma metabolomic profiles was performed by a commercial laboratory (Metabolon, Morrisville, NC) as previously described [21]. Briefly, extracted supernatant was split and run on gas chromatography and liquid chromatography mass spectrometer platforms in randomized order. Gas chromatography (for hydrophobic molecules) and liquid chromatography (for hydrophilic molecules) were used to identify and provide relative quantification of small metabolites present in plasma samples. Endogenous biochemical included amino acids, peptides, carbohydrates, lipids, nucleotides, cofactors and vitamins. The complete plasma dataset is shown as a heat map of statistically significant biochemicals profiled in this study (S1 Table).

### Fecal sample collection and fecal microbiome

Fecal samples were collected, homogenized, and frozen as aliquots within 1 hour of defecation. Whole feces were collected after defecation and homogenized thoroughly using Thinky Mixer model ARM-310 (THINKY USA, Inc). Homogenous samples were aliquoted into labeled cryovials. The tubes were snap-frozen immediately in liquid nitrogen followed by storing at −80°C until further processing.

The fecal microbiome analysis was performed as previously published [22], except total DNA was extracted from frozen feces samples using the Qiagen MagAttract Power Microbiome DNA/RNA EP DNA isolation kit (Qiagen Cat. No. ID:27500-4-EP) optimized for use with the Eppendorf epMotion 5075 TMX platform (Eppendorf, AG, Hamburg). In brief, polymerase chain reaction (PCR) amplification spanned the V3-V4 hypervariable regions of the 16S rRNA gene, amplicon sequencing was performed using the Illumina library preparation protocol (15044223 Rev. A), sequences were de-multiplexed to obtain FASTQ Files, and bacterial taxonomic classification was per the GreenGenes reference taxonomy. Copy numbers of the 16S genes in their respective taxa were corrected using Phylogenetic Investigation of Communities by Reconstruction of Unobserved States (PICRUSt). Samples with fewer than 5000 reads were removed from analysis. The mean ± SD number of reads per sample was 62,126.7 ± 14,930. Zeros were imputed using Bayesian multiplicative treatment and natural log centered log-ratio (CLR) transformation of the copy-corrected operational taxonomic units (OTU) count data was performed to enable appropriate statistical analysis. Further statistical analyses were performed on the data as described below. The sequences were deposited in NCBI SRA under accession number PRJNA616033.

### Statistical methods

Statistical analyses were performed in JMP, version 14, and SAS, version 9.4 (both SAS Institute, Cary, NC) for food intake, body weights, CBC, serum chemistries, urinalyses, and urine protein/creatinine ratios. These data were normally distributed. Metabolomics data were log transformed before analyses were performed using ArraySudio (Omissoft Corporation, Cary NC). A mixed model was used with cat identity as random effect to test whether means were different based on health status of cats (healthy versus CKD), type of fiber fed (scFOS versus apple pomace), or an interaction between health status and type of fiber fed. This test allows for unequal variances and has an approximate *t*-distribution with degrees of freedom estimated using Satterthwaite’s approximation. Significance was established when *P* ≤ 0.05 (for type 1 error) and *q* ≤ 0.1 (*q*-values were used to estimate false discovery rate in multiple comparisons). The mixed model was then used to test whether the difference of two paired observations from a single cat at baseline and the end of the 4 week feeding period was different than zero. This test was also used to test whether means were different after treatment with apple pomace versus scFOS as a fiber source within healthy cats and within CKD cats.

**Fig 1:**
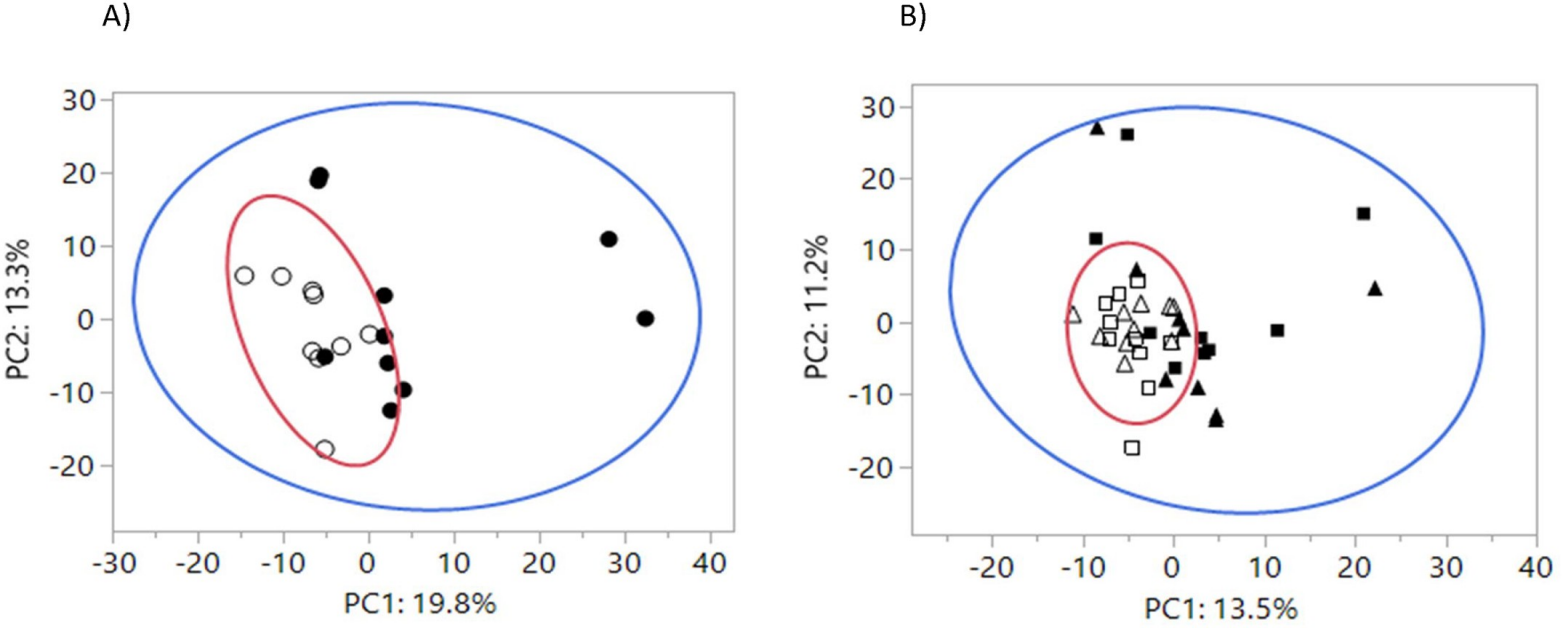
Principal component analysis (PCA) of plasma metabolites. Large circles represent 95% variance areas. A) Plasma metabolites at baseline for healthy cats (open circles) and CKD cats (closed circles). Means and variance for PC1 were different by health (*P* < 0.05). B) Plasma metabolites after feeding scFOS (open squares) or apple pomace (open triangles) for 4 weeks to healthy cats, and after feeding scFOS (closed squares) or apple pomace (closed triangles) for 4 weeks to CKD cats. Means and variance for PC1 and variance for PC2 were different by health (*P* < 0.05).

An independent *t*-test was used to test for differences of the means of the principal components (PC) in the principal component analysis (PCA) of plasma metabolomics data at baseline comparing healthy and CKD cats (Fig 1A). The mixed model was then used to test the means of PC1 and PC2 after treatment with fiber sources (Fig 1B). Levene’s test was used to test whether the variance of PC1 and PC2 in the PCA analysis were different.

For microbiome analysis, the CLR transformed data were further analyzed using JMP with unpaired *t*-tests for disease state comparisons (healthy versus CKD cats) and paired *t*-tests for effects of fiber (scFOS and apple pomace). In order to minimize imputed numbers, >80% of cats in any one group had to have that OTU. Significance was established when *P* ≤ 0.05 (for type 1 error) and *q* ≤ 0.1 (to estimate false discovery rate in multiple comparisons). For microbiome taxonomic classification, chi-square analysis was performed on operational taxonomic units (OTU) differences. Linear regression analyses are reported by Pearson’s correlation coefficient (*r*) and *P*-values. Log transformation was applied to non-normally distributed variables. Statistical significance was attested at *P* ≤ 0.05.

Alpha diversity was calculated as described in Jackson & Jewell [22] on genus-level count data in the R programming environment [23] using the R vegan package [24]. Four alpha diversity metrics were assessed: Taxa richness (S) indicates the number of unique genus level taxa detected whereas Pielou’s Evenness (J) indicates the heterogeneity of the relative abundances of these taxa. The Shannon and Simpson indices both factor in richness and evenness. While the Shannon index considers all taxa according to their abundance, in the Simpson index the highly abundant taxa are greater contributors to the final value. The exponent of the Shannon index (expH) and the inverse of the Simpson index (invSimp) were used for statistical analysis and reporting, as these respective transformations facilitate direct comparisons between the two indices by placing them on the same scale. Statistical comparison of alpha diversity metrics across health status were performed as Student’s *t*-test (unpaired), whereas comparisons within health status were performed using a within-animal paired *t*-test. Phylogenetically derived abundance weighted UniFrac distances were also calculated and plotted as PCoA using the R package phyloseq [25].

## Results and discussion

Microbial fermentation of dietary fiber in the hindgut of cats is considered important for health even though cats are obligate carnivores [26]. The scFOS fed in this study is a well-defined fiber that is quickly fermented. Apple pomace, on the other hand, is composed of fiber, and free and bound polyphenols, which are slowly fermented in the hind gut. It is a by-product of the apple juice industry, and a rich source of fibers including pectins, cellulose, hemicelluloses, lignins and gums. Crude fiber, which represents the type of dietary fiber that remains as a residue after food receives a standardized laboratory treatment with dilute acid and alkali, was similar for the two foods. Such treatment dissolves all soluble fibers and some insoluble fibers. The residue, or crude fiber, is primarily composed of cellulose and lignin. Insoluble fiber, some of which can be fermented by colonic bacteria, adds bulk to stools and was 50% higher in the food containing apple pomace as the fiber source compared with food containing scFOS. Soluble fiber absorbs water and becomes a gel during the digestive process. Some soluble fiber is readily fermented in the colon) generating gases and physiologically active by-products. Food containing apple pomace was 100% higher in soluble fiber compared with food containing scFOS.

The effects of dietary fiber in cats with diseases such as CKD, as well as the best type of fiber to feed to subjects with this health condition, has not been well studied. Previous studies in rats have shown that blood urea decreases when feeding fermentable fiber because of the nitrogen trap principle [27]. In addition, fermentation of soluble fibers in cats [28–30] and dogs [31] may spare the amino acid valine by producing propionic acid as a glucose precursor. In most of the studies reviewed on scFOS supplementation in cats, an increase in fecal *Bifidobacterium spp* was observed [26].

The purpose of this study was to evaluate the effects of feeding betaine, oat beta glucan, and either scFOS or apple pomace to healthy cats and cats with CKD (IRIS stages 1 and 2). We assessed plasma metabolite concentrations as well as the fecal microbiome. We hypothesized that the type of fermentable fiber fed could influence the fecal microbial composition and, in particular, microbiota generated phenolic and indolic uremic toxins. Finally, we hypothesized that changes in the gastrointestinal tract might also affect plasma metabolites associated with inflammation and oxidative stress.

### Food intake and body weights

Food intake was not affected by inclusion of fiber in the diet. The intake of food A with scFOS (63.57 ± 3.66 kcal/kg^0.75^/day) was similar to the intake of food B containing apple pomace (59.10 ± 3.76 kcal/kg^0.75^/day; *P* = 0.27). Health status did not influence mean food intake (65.1 ± 4.3 kcal/kg^0.75^/day in healthy cats versus 61.9 ± 4.3 kcal/kg^0.75^/day in CKD cats; *P* = 0.46). Body weights at baseline were lower for healthy cats compared with CKD cats (*P* = 0.03), and did not change after consuming test foods.

### Baseline and treatment serum chemistries and urine analytes based on health status of cats and fiber source

CBC and serum chemistry profiles were obtained following an overnight fast at baseline and at the end of each 4-week feeding period (Table 3). Values not reported were within normal reference intervals. At baseline, CKD cats had higher serum Cr and blood urea nitrogen (BUN) and lower albumin concentrations (all *P* <0.05) compared with healthy cats. However, all cats had serum albumin concentrations in the normal reference interval (2.5 to 3.8 g/dL).

**Table 3:** Serum chemistries and urine analytes in healthy cats (H cats) and chronic kidney disease (CKD) cats at baseline (BSL, after feeding pre-trial food^1^ for 14 days), and after feeding Food A^2^ (containing scFOS) or Food B^3^ (containing apple pomace) as fiber sources for 4 weeks each.

	Mean Values						Group Effect^‡^				
	H Cats			CKD Cats			Overall Effect of Health	Overall Effect of Both Treatments	H Cats: Treatment with Food B versus Food A^¥^	CKD Cats: Treatment with Food B versus Food A^¥^	Overall Effect of Interaction
Serum Chemistries	BSL	Food A	Food B	BSL	Food A	Food B	*P*-value	*P*-value	*P*-value	*P*-value	*P*-value
Cr, mg/dL	1.23^#^	1.23	1.25	1.58^#^	1.47	1.41	0.09	0.65	0.60	0.30	0.42
BUN, mg/dL	16.8^#^	17.3	16.9	18.6^#^	18.0	19.4	0.20	0.75	0.24	0.49	0.19
Albumin, g/dL	3.34^#^	3.28	3.34	3.07^#^	3.07	3.06	0.02	0.50	0.17	0.90	0.66
**Urine Analytes**											
pH	5.96^#^	5.99	5.77	6.24^#^	6.31	6.15	0.03	0.02	0.03	0.15	0.58
USG	1.046^#^	1.046	1.045	1.039^#^	1.041	1.039	0.03	0.32	0.66	0.33	0.69

^1^Baseline (BSL; pre-trial) food was Prescription Diet^®^ k/d^®^ Feline with chicken, dry.

^2^ Food A was prepared by Hill’s Pet Nutrition, Inc. and was similar to the pre-trial food, with the exception that it was supplemented with betaine (0.500%), oat beta glucan (0.586%), and 0.407% short chain FOS (scFOS).

^3^Food B was prepared by Hill’s Pet Nutrition, Inc. and was similar to the pre-trial food, with the exception that it contained 0.500% betaine, 0.586% oat beta glucan, and 3.44% apple pomace.

^‡^A mixed model was used with cat identity as random effect to test whether means were different based on health status of cats (healthy versus CKD), type of fiber fed (scFOS versus apple pomace), or an interaction between health status and type of fiber fed.

^¥^The mixed model was used to test whether means were different after treatment with apple pomace versus scFOS as a fiber source within healthy cats and within CKD cats.

^#^The mixed model was used to test whether means were different at baseline based on health status of cats (healthy versus CKD).

Significance was established when *P* ≤ 0.05 (for type 1 error).

Urinalysis and urine protein/creatinine ratios were also compared at baseline and at the end of each 4-week feeding period. At baseline, urine pH was higher and urine specific gravity (USG) was lower (both *P* <0.05) in CKD cats compared with healthy cats. Urine pH was lower in healthy cats (*P* = 0.03) after consuming Food B (apple pomace) compared with Food A (scFOS). No differences were observed in urine protein/creatinine ratio. Thus, overall differences in serum chemistry and urine analytes were mainly the result of health status rather than feeding different fiber sources, although urine pH was decreased in healthy cats after consuming apple pomace.

### Plasma metabolomics at baseline and after treatment with different fiber sources

Plasma metabolite concentrations were compared at baseline and at the end of each 4-week feeding period for healthy and CKD cats. In total, 701 metabolites were detected across all test times. Of these, 54 plasma metabolites were different between healthy cats and CKD cats at baseline (after the 14-day pre-trial feeding period). Amongst these metabolites, 38 were increased and 16 were decreased in CKD cats compared with healthy cats.

After feeding Food A (scFOS) as a fiber source for 4 weeks: 58 metabolites differed from baseline in healthy cats and 51 metabolites differed from baseline in CKD cats. After feeding Food B (apple pomace) as a fiber source for 4 weeks, 48 metabolites differed from baseline in healthy cats and 68 metabolites differed from baseline in CKD cats. Overall, there was a greater number of metabolites that changed from baseline in CKD cats after consuming apple pomace vs a greater number of metabolites than changed from baseline in healthy cats after consuming scFOS.

To gain insight into how fiber treatments impacted global plasma metabolomics data, a 2-dimensional principal component analysis (PCA) was performed, which groups metabolites that track together and reduces the dimensions of the data down to two linear components. There was no separation of the 95% confidence areas for healthy cats and cats with CKD at baseline based on plasma metabolites (Fig 1A), although the means of principal component 1 (PC1) for healthy and CKD groups were different (*P* = 0.021). The top eigenvector metabolites that contributed to the separation of the two groups included urea, 3-hydroxy-2-ethylpropionate, creatine and tryptophan. S2 Table includes all eigenvectors in PC1. The means of principal component 2 (PC2) were not different by health status (*P* = 0.17).

After feeding the two fiber sources for 4 weeks each there was still no separation of the 95% confidence areas based on health status (Fig 1B); the means for PC1 were again different between healthy and CKD groups (mean ± SEM, -4.5 ± 0.65 and 4.8 ± 2.75, respectively; *P* = 0.027). S3 Table includes all eigenvectors in PC1. The PC2 means were not different between healthy and CKD groups (mean ± SEM, -1.6 ± 1.15 and 1.64 ± 2.64, respectively; *P* = 0.4). Overall, there was no difference in plasma metabolites based on type of fiber fed (PCA not shown; both PC1 and PC2 *P* > 0.10). Nonetheless, individual metabolite changes that differed based on type of fiber fed are noted below in Tables 4 and 5.

**Table 4:** Relative plasma metabolite concentrations for antioxidants, markers of oxidation and inflammation, methylated metabolites, TCA cycle, and urea cycle intermediates in healthy cats (H cats) and chronic kidney disease (CKD) cats at baseline (after feeding pretrial food^1^ for 14 days) and after feeding Food A^2^ (containing scFOS) or Food B^3^ (containing apple pomace) as fiber sources for 4 weeks each.

	Mean Values*						Group Effect^‡^				
	H Cats			CKD Cats			Overall Effect of Health	Overall Effect of Both Treatments	H Cats: Treatment with Food B versus Food A^¥^	CKD Cats: Treatment with Food B versus Food A^¥^	Overall Effect of Interaction
Metabolites	BSL	Food A	Food B	BSL	Food A	Food B	*P*-value	*P*-value	*P*-value	*P*-value	*P*-value
**Tocopherol Metabolism**											
α-tocopherol	0.99	0.97	1.05^§^	0.90	0.91	0.95	0.08	0.04	0.01	0.62	0.28
α-CEHC sulfate	0.95	0.93	1.13	1.63	3.12^§^	2.48	0.78	0.36	0.46	0.43	0.08
γ-tocopherol/β-tocopherol	1.75^#^	0.84^§^	1.49	1.18^#^	0.79^§^	1.15	0.03	0.000	0.002	0.08	0.43
**Glutathione Metabolism**											
oxidized glutathione (GSSG)	0.83	1.34^§^	1.03	1.20	0.97	1.58	0.41	0.26	0.21	0.03	0.02
Cysteine-glutathione disulfide	0.88	1.12^§^	0.97	0.99	0.99	1.23^§^	0.31	0.01	0.04	0.012	0.005
cysteinylglycine	0.68	1.41^§^	1.09^§^	1.15	1.33	1.84	0.32	0.03	0.74	0.34	0.42
cysteinylglycine disulfide	0.72	1.06^§^	1.01^§^	0.85	1.10^§^	1.22^§^	0.12	0.000	0.58	0.15	0.27
5-oxoproline	0.93	0.98	0.92	1.11	1.13	1.10	0.03	0.38	0.13	0.77	0.44
2-aminobutyrate	0.77	1.12^§^	1.28^§^	0.73	1.00^§^	1.06^§^	0.49	0.000	0.16	0.53	0.40
2-hydroxybutyrate/2-hydroxyisobutyrate	0.89	1.40^§^	1.39^§^	1.44	1.76^§^	1.72^§^	0.45	0.000	0.76	0.91	0.13
Ophthalmate	0.27	0.96^§^	0.93^§^	0.93	2.07^§^	1.17^§^	0.12	0.000	0.52	0.17	0.72
**Sphingolipid Metabolism**											
Sphinganine	0.81	2.55^§^	1.02	1.10	1.30	2.65^§^	0.28	0.004	0.003	0.012	0.001
sphinganine-1-phosphate	0.93	2.66^§^	0.95	1.08	1.28	3.24^§^	0.58	0.01	0.002	0.019	0.001
Sphingosine	0.80	2.92^§^	0.99	1.01	1.21	2.84^§^	0.20	0.005	0.004	0.008	0.001
sphingosine 1-phosphate	0.89	1.71^§^	0.95	0.93	1.03	1.84^§^	0.63	0.01	0.007	0.009	0.001
sphingadienine	0.93	2.20^§^	0.91	0.99	1.10	2.12^§^	0.56	0.03	0.001	0.005	0.000
**Glycine, Serine, & Threonine Metabolism**											
Glycine	0.94^#^	1.00^§^	0.98	1.13^#^	1.10	1.12	0.049	0.43	0.53	0.31	0.14
Sarcosine	1.44	0.79^§^	0.95^§^	1.49	0.83^§^	0.88^§^	0.94	<0.001	0.038	0.74	0.35
dimethylglycine	0.80	1.31^§^	1.43^§^	0.86	1.13^§^	1.10^§^	0.23	<0.001	0.16	0.62	0.03
Betaine	0.46	1.15^§^	1.30^§^	0.54	1.19^§^	1.37^§^	0.17	<0.001	0.09	0.15	0.43
S-adenosylhomocysteine (SAH)	0.75^#^	1.03	0.65	1.52^#^	1.12^§^	0.98^§^	0.005	0.0387	0.020	0.46	0.02
**TCA Cycle**											
Citrate	0.96	0.98	1.05^§^	1.11	1.09	1.02^§^	0.36	0.81	0.046	0.18	0.01
aconitate [cis or trans]	0.82^#^	1.18^§^	0.95^§^	1.12^#^	1.10	1.23^§^	0.09	0.000	0.000	0.004	0.000
alpha-ketoglutarate	1.11	1.11	1.35^§^	0.94	0.99	0.91	0.20	0.49	0.004	0.13	0.01
succinylcarnitine	1.03	0.97	1.01	1.38	1.35	1.25	0.13	0.56	0.40	0.55	0.74
Succinate	1.12^#^	1.28	1.07	0.90^#^	0.91	1.18^§^	0.08	0.53	0.12	0.012	0.008
Malate	1.33	1.46	1.43	0.96	0.88	0.98	0.03	0.61	0.64	0.36	0.76
**Urea Cycle**											
Arginine	1.05	1.03	1.00	1.05	1.02	1.03	0.85	0.73	0.58	0.99	0.88
argininosuccinate	0.76	0.88	0.92	1.11	1.02	0.72^§^	0.59	0.39	0.63	0.10	0.03
Urea	0.87^#^	0.93^§^	0.91	1.48^#^	1.31^§^	1.24	0.03	0.99	0.66	0.55	0.01
Ornithine	0.94	0.98	1.02	1.05	1.02	1.13	0.20	0.08	0.43	0.08	0.41
Citrulline	0.95^#^	0.88	0.85	1.30^#^	1.19	1.08^§^	0.01	0.01	0.76	0.23	0.70
**Arginine and Proline Metabolism**											
2-oxoarginine	0.97	1.08	1.00	0.78	0.90	0.87	0.20	0.10	0.42	0.58	0.61
Homoarginine	1.40	1.52	1.70	1.08	1.34	1.23	0.57	0.33	0.29	0.79	0.65
Homocitrulline	0.65	1.11^§^	1.08^§^	1.22	1.89^§^	1.44^§^	0.07	0.000	0.83	0.26	0.39
Proline	0.95	1.01	1.02	0.99	0.94	1.08	0.79	0.08	0.92	0.026	0.18
dimethylarginine (SDMA + ADMA)	0.88^#^	0.91	0.93	1.20^#^	1.21	1.14	0.002	0.77	0.56	0.82	0.52
N-acetylarginine	0.87^#^	0.94^§^	0.94^§^	1.17^#^	1.16	1.11	0.01	0.14	0.93	0.71	0.01
N-delta-acetylornithine	0.98	0.93	1.00	1.22	1.17	1.18	0.34	0.70	0.52	0.79	0.96
trans-4-hydroxyproline	1.12	0.93^§^	0.91	1.23	1.08	1.08	0.25	0.03	0.81	0.99	0.93
pro-hydroxy-pro	1.44	0.85^§^	0.92^§^	1.20	0.95^§^	0.92^§^	0.73	0.000	0.25	0.88	0.03
N-methylproline	1.04	1.10	0.92	1.30	1.29	1.10	0.36	0.12	0.016	0.74	0.30
N-monomethylarginine	1.00	0.94	0.92	1.13	1.04	1.07	0.09	0.11	0.60	0.43	0.64
Argininate	1.12	1.71^§^	1.59^§^	1.30	1.64^§^	1.27^§^	0.30	0.000	0.49	0.81	0.95

^**1–3, ‡**, ¥, #^ See Table 3.

*For each metabolite, mean value is the group mean of re-scaled data to have median equal to 1.

^§^The mixed model was used to test whether the difference of two paired observations from a single cat at baseline and the end of the 4 week feeding period was different than zero.

Significance was established when *P* ≤ 0.05 (for type 1 error) and *q* ≤ 0.1 (*q*-values were used to estimate false discovery rate in multiple comparisons).

**Table 5:** Relative plasma metabolite concentrations for other renal-associated markers and metabolites in healthy cats (H cats) and CKD cats at baseline (after feeding pretrial food^1^ for 14 days) and after feeding food A^2^ (containing scFOS) or food B^3^ (containing apple pomace) as fiber sources for 4 weeks each.

	Mean Values*						Group Effect^‡^				
	H Cats			CKD Cats			Overall Effect of Health	Overall Effect of Both Treatments	H Cats: Treatment with Food B vs Food A^¥^	CKD Cats: Treatment with Food B vs Food A^¥^	Overall Effect of Interaction
Metabolites	BSL	Food A	Food B	BSL	Food A	Food B	*P*-value	*P*-value	*P*-value	*P*-value	*P*-value
**Creatine Metabolism**											
Guanidinoacetate	0.94	1.08^§^	1.02	0.92	1.27^§^	1.25^§^	0.67	0.001	0.48	0.81	0.35
Creatine	1.01	1.06	0.97	0.93	0.88	1.06^§^	0.43	0.46	0.052	0.001	0.001
Creatinine	0.98	0.95	0.97	1.12	1.12	1.05	0.11	0.69	0.42	0.78	0.55
**Tryptophan Metabolism**											
Tryptophan	1.10	1.03^§^	1.07	0.95	0.93	0.98	0.08	0.22	0.27	0.77	0.67
N-acetyltryptophan	1.03	1.03	1.06	1.95	1.16^§^	1.61^§^	0.29	0.11	0.81	0.18	0.03
C-glycosyltryptophan	0.79^#^	0.99^§^	0.83	1.31^#^	1.41	1.18	0.003	0.11	0.047	0.56	0.50
tryptophan betaine	0.08	1.23^§^	1.21^§^	0.08	0.97^§^	1.24^§^	0.85	0.000	0.84	0.34	0.61
Kynurenine	0.94	0.98	0.89	1.01	1.07	1.15	0.34	0.23	0.048	0.74	0.17
N-acetylkynurenine (2)	0.97	0.91	1.18	1.37	1.05	1.61	0.90	0.10	0.11	0.15	0.75
Kynurenate	0.85^#^	0.72	0.79	1.30^#^	1.27	1.22	0.001	0.58	0.53	0.80	0.92
Anthranilate	0.57	1.02^§^	0.98	0.86	1.03	1.33	0.06	0.06	0.44	0.46	0.49
Picolinate	0.94	1.02	1.09^§^	1.05	1.16	1.24	0.56	0.06	0.41	0.81	0.85
Serotonin	0.90	1.94	0.96	0.93	1.08	1.65	0.74	0.46	0.22	0.41	0.28
5-hydroxyindoleacetate	0.80^#^	0.89	0.89	1.61^#^	1.21^§^	1.19	0.02	0.43	0.96	0.32	0.25
Indolelactate	1.18	1.13	1.22	1.33	1.10^§^	1.28	0.44	0.06	0.24	0.34	0.19
Indoleacetate	0.96	1.05	1.09	0.88	1.52^§^	1.86^§^	0.34	0.01	0.24	0.27	0.25
Indolepropionate	1.41	1.15	1.64	1.47	1.27	0.83	0.72	0.41	0.78	0.07	0.30
Indoleacetylglutamine	0.71	0.89	0.86^§^	1.13	1.66	1.91	0.06	0.03	0.48	0.45	0.94
Indoleacetylglycine	0.97	0.91	1.03	1.56	1.82	2.11	0.04	0.21	0.09	0.56	0.74
3-indoxylsulfate	0.60^#^	1.77	1.31	4.06^#^	2.87	1.57	0.05	0.17	0.24	0.14	0.46
5-hydroxyindole sulfate	0.24^#^	1.26	0.86	2.81^#^	1.89	0.86	0.11	0.13	0.18	0.14	0.30
6-hydroxyindole sulfate	0.56	2.71	2.06	6.75	4.32	2.03	0.09	0.16	0.22	0.14	0.48
7-hydroxyindole sulfate	0.29^#^	0.93^§^	0.68	1.60^#^	0.98	0.54	0.14	0.16	0.13	0.24	0.07
5-bromotryptophan	0.90	1.20^§^	1.14^§^	0.95	0.99	1.04	0.25	0.02	0.53	0.48	0.48
**Benzoate Metabolism**											
Hippurate	1.51	1.69	1.13	4.76	4.00	5.14	0.15	0.81	0.66	0.68	0.55
3-hydroxyhippurate	1.18	0.33^§^	0.36^§^	1.97	0.59^§^	0.59^§^	0.07	0.000	0.79	0.80	0.89
4-hydroxyhippurate	1.18	0.68^§^	0.87	2.25	1.81	2.46	0.02	0.02	0.16	0.29	0.89
Benzoate	1.09	0.93	0.93	1.05	1.27	1.44	0.05	0.89	0.92	0.92	0.19
catechol sulfate	1.52	0.84^§^	0.94	1.41	1.06	1.33	0.86	0.08	0.27	0.30	0.32
guaiacol sulfate	2.76	0.49^§^	0.78^§^	1.89	0.56^§^	1.15	0.80	0.000	0.07	0.050	0.31
4-methylcatechol sulfate	1.21	1.10	1.53	1.77	1.46	1.57	0.91	0.20	0.10	0.48	0.61
4-acetylphenol sulfate	0.63	0.42	0.35	1.21	0.69	0.95	0.03	0.10	0.71	0.61	0.80
4-ethylphenylsulfate	0.51	2.80^§^	2.36^§^	0.99	1.07	1.46^§^	0.81	0.000	0.88	0.18	0.04
4-vinylphenol sulfate	0.95	0.71	1.08	1.99	0.96^§^	1.64	0.37	0.001	0.019	0.003	0.62
3-methoxycatechol sulfate (1)	1.81	0.53^§^	1.45	2.04	0.30	0.81	0.31	0.004	0.029	0.10	0.88
3-methoxycatechol sulfate (2)	0.90	0.71	0.68	1.21	0.64^§^	0.98	0.69	0.05	0.78	0.16	0.64
methyl-4-hydroxybenzoate sulfate	0.39	0.37	0.39	0.37	8.63^§^	0.98	0.03	0.13	0.91	0.06	0.10
p-cresol sulfate	0.85	1.01	1.05	1.78	1.53	1.21	0.99	0.57	0.67	0.97	0.91
Phenylpropionylglycine	0.78	0.99	0.50	3.40	2.56	2.92	0.24	0.66	0.21	0.92	0.53
3-(3-hydroxyphenyl)propionate sulfate	1.80	0.51^§^	0.71^§^	2.22	0.63^§^	0.97	0.95	0.000	0.20	0.23	0.64
3-(3-hydroxyphenyl)propionate	1.49	0.52^§^	0.47^§^	1.50	0.71^§^	0.61	0.99	0.000	0.68	0.72	0.26
3-(4-hydroxyphenyl)propionate	0.92	0.50^§^	0.71	1.04	1.42	1.66	0.21	0.41	0.46	0.62	0.26
3-phenylpropionate (hydrocinnamate)	1.18	1.42	1.17	1.44	2.77	5.08^§^	0.21	0.07	0.93	0.26	0.19
**Pyrimidine Metabolism, Uracil derived**											
Pseudouridine	0.85^#^	0.98^§^	0.90	1.31^#^	1.30	1.08^§^	0.002	0.05	0.09	0.10	0.03
**Pyrimidine Metabolism, Thymine derived**											
3-aminoisobutyrate	1.12	0.81^§^	0.68^§^	3.77	3.75	4.38	0.02	0.001	0.14	0.34	0.02

^**1–3, ‡**, ¥, #^ See Table 3.

*For each metabolite, mean value is the group mean of re-scaled data to have median equal to 1.

^§^The mixed model was used to test whether the difference of two paired observations from a single cat at baseline and the end of the 4 week feeding period was different than zero.

Significance was established when *P* ≤ 0.05 (for type 1 error) and *q* ≤ 0.1 (*q*-values were used to estimate false discovery rate in multiple comparisons).

Variance in plasma metabolites based on health status was expressed by denoting the 95% confidence areas with ellipses around healthy cats and CKD cats at baseline and after feeding fiber sources for 4 weeks. Variance was greater for CKD cats compared with healthy cats both at baseline (PC1, *P* = 0.03; PC2, *P* = 0.17; Fig 1A) and after feeding both fiber sources for 4 weeks (PC1, *P* = 0.006; PC2, *P* = 0.006; Fig 1B).

### Treatment differences in individual plasma metabolomics based on health status of cats and fiber source fed

We have previously reported that gastrointestinal microbial metabolites are readily modified in healthy cats after consuming their preferred macronutrient intake for 28 days (16/38 metabolites or 42% changed) [21], and that older cats fed similar protein intake as in this study had higher concentrations of sulfated microbial catabolic products (p-cresol sulfate and 4-ethyl phenyl sulfate) compared with younger, leaner cats [32]. In the current study, global metabolite analysis indicated CKD cats were different from healthy cats at baseline and responded differently than healthy cats to fiber supplementation. Examples of these differences are highlighted for the following selected categories of metabolites (antioxidants, markers of oxidation and inflammation, methylated compounds, TCA cycle, urea cycle, other renal-associated metabolites).

Markers of oxidation included those involved in tocopherol metabolism and those involved in glutathione metabolism (Table 4). At baseline, γ-tocopherol/β-tocopherol concentrations were significantly lower in CKD cats compared with healthy cats. Across all time points of the study, there were two significant differences based on overall effect of health (healthy cats versus CKD cats). The CKD cats had lower concentrations of γ-tocopherol/β-tocopherol and higher concentrations of 5-oxoproline. Most of the oxidative metabolites in healthy and CKD cats were affected by treatment with fiber (both scFOS and apple pomace). Compared with baseline, healthy cats had higher concentrations of α-tocopherol after consuming apple pomace, and both healthy cats and CKD cats had lower concentrations of γ-tocopherol/β-tocopherol after consuming scFOS compared with baseline. The CKD cats also had higher concentrations of the oxidized tocopherol metabolite alpha-carboxyethylhydroxychroman sulfate (α-CEHC sulfate) compared with baseline after consuming scFOS. Compared with baseline, healthy cats had higher concentrations of most glutathione metabolites after consuming scFOS; fewer significant differences were noted after consuming apple pomace. The CKD cats had higher concentrations of a larger number of glutathione metabolites after consuming apple pomace relative to the same cats consuming scFOS. Comparing the two fiber sources, healthy cats had higher concentrations of α-tocopherol and γ-tocopherol/β-tocopherol after consuming apple pomace as a fiber source. CKD cats on the other hand, had no significant differences in tocopherol metabolites, but the glutathione metabolite cysteine-glutathione disulfide was increased after consuming apple pomace as a fiber source. Overall, there was a significant interaction of health status and fiber source for cysteine-glutathione disulfide likely driven by the observation that healthy cats had higher concentrations after consuming scFOS and CKD cats had higher concentrations after consuming apple pomace as a fiber source. In summary, important effects at baseline for markers of oxidation (tocopherol and glutathione metabolism) were that γ/β tocopherol concentrations were lower in CKD cats compared with healthy cats, and decreased further after consuming scFOS. Healthy cats had greater concentrations of α-tocopherol after consuming apple pomace. The CKD cats had higher concentrations of more oxidized glutathione metabolites after consuming apple pomace compared with scFOS.

Because sphingolipid metabolites, particularly sphingosine-1-phosphate, are signaling molecules that regulate a diverse range of cellular processes that are important in immunity, inflammation and inflammatory disorders [17, 18], we analyzed sphingolipid metabolites as markers of inflammation. There were no significant differences based on overall effect of health (healthy cats versus CKD cats) at baseline or across all time points of the study. However, all sphingolipid metabolites were significantly affected by treatment with fiber (both scFOS and apple pomace) in healthy and CKD cats. Healthy cats had higher concentrations of all sphingolipid metabolites compared with baseline after consuming scFOS, yet no significant differences for sphingolipid metabolites after consuming apple pomace. The CKD cats had higher concentrations of all the sphingolipid metabolites after consuming apple pomace; none of the sphingolipid metabolites were increased in the CKD cats after consuming scFOS compared to baseline. Comparing the two fiber sources, healthy cats had greater amounts of all the sphingolipid metabolites after consuming scFOS as a fiber source. CKD cats on the other hand, had higher concentrations of three of four sphingolipid metabolites after consuming apple pomace as a fiber source. Thus, there was an overall interaction for all four sphingolipid metabolites because healthy cats had increases in sphingolipid metabolites after consuming scFOS, but not after consuming apple pomace as a fiber source, whereas CKD cats had increases in sphingolipid metabolites after consuming apple pomace, but not after consuming scFOS as a fiber source. In summary, important effects for sphingolipid metabolites were that healthy cats had higher concentrations of all sphingolipid metabolites compared with baseline after consuming scFOS, but not after consuming apple pomace. Compared to baseline, CKD cats had higher concentrations of all sphingolipid metabolites after consuming apple pomace, but not after consuming scFOS.

At baseline, for the metabolites related to methylation, glycine and S-adenosylhomocysteine (SAH) concentrations were significantly higher in CKD cats compared with healthy cats. Across all time points of the study, based on overall effect of health (healthy cats versus CKD cats) only SAH concentrations were significantly higher in CKD cats. Several metabolites of both healthy and CKD cats were affected by treatment with fiber containing foods (both scFOS and apple pomace foods, which also contained added betaine), including sarcosine, dimethylglycine, betaine, and SAH. Compared with baseline, healthy cats had higher concentrations of glycine after consumption of scFOS, and higher concentrations of dimethylglycine and betaine, but lower concentrations of sarcosine after consumption of both fiber sources. Compared with baseline, CKD cats had higher concentrations of dimethylglycine and betaine, but lower concentrations of sarcosine and SAH after consumption of both fiber sources. There were no effects of treatment with apple pomace versus scFOS in healthy cats or CKD cats, and no overall effect of interaction for methylated metabolites. In summary, important effects for methylated metabolites were that at baseline, glycine and SAH concentrations were higher in CKD cats compared with healthy cats. In both healthy and CKD cats, consumption of betaine in both treatment foods increased concentrations of dimethylglycine and betaine, and decreased concentrations of sarcosine (also SAH in CKD cats).

At baseline, for tricarboxylic acid cycle (TCA) and urea cycle metabolites, aconitate (cis or trans) concentrations were significantly higher and succinate concentrations were significantly lower in CKD cats compared with healthy cats. At baseline in the urea cycle, urea and citrulline, and in the arginine and proline metabolism category, the dimethylarginines (SDMA and ADMA), and N-acetylarginine concentrations were significantly higher in CKD cats compared with healthy cats. Across all time points of the study, these metabolites, based on overall effect of health (healthy cats versus CKD cats) continued to have higher concentrations in CKD cats. Several metabolites in the TCA and urea cycles of both healthy and CKD cats were affected by treatment with fiber (both scFOS and apple pomace), including aconitate in the TCA cycle, citrulline in the urea cycle, and homocitrulline, trans-4-hydroxyproline, pro-hydroxy-pro, and arginate in the arginine and proline metabolism category of associated amino acid metabolites. Compared with baseline, healthy cats had higher concentrations of aconitate after consuming both scFOS and apple pomace, whereas CKD cats had higher concentrations only after consuming apple pomace. Compared with baseline, healthy cats also had higher concentrations of citrate and alpha-ketoglutarate (TCA cycle metabolites), after consuming apple pomace, whereas CKD cats had higher concentrations of succinate and lower concentrations of citrate after consuming apple pomace. In the arginine and proline metabolism category, compared with baseline, healthy cats had higher concentrations of homocitrulline, N-acetylarginine, and arginate after consuming both fiber types, and lower concentrations of pro-hydroxy-pro. Healthy cats also had higher concentrations of urea and lower concentrations of trans-4-hydroxyproline after consuming scFOS compared with baseline. Compared with baseline, CKD cats had lower concentrations of arginosuccinate, citrulline, pro-hydroxy-pro, and arginate after consuming apple pomace. Homocitrulline was increased in CKD cats after consuming both fiber sources compared with baseline. Urea and pro-hydroxy-pro were decreased and arginate was increased in CKD cats after consuming scFOS as a fiber source. Comparing the two fiber sources, healthy cats had greater amounts of aconitate after consuming scFOS and greater amounts of alpha-ketoglutarate after consuming apple pomace. CKD cats had greater amounts of aconitate after consuming apple pomace. Thus, there was an overall interaction for TCA cycle metabolites because healthy cats had greater increases in citrate than CKD cats after consuming apple pomace, greater increases in aconitate after consuming scFOS, smaller increases in aconitate after consuming apple pomace, and greater increases in alpha-ketoglutarate after consuming apple pomace. The CKD cats had greater increases in aconitate and succinate after consuming apple pomace. For the urea cycle, and for arginine and proline metabolites, there were no effects of treatment with apple pomace versus scFOS in healthy cats or in CKD cats. There was an overall effect of interaction for urea (increased in healthy cats fed scFOS and decreased in CKD cats fed scFOS) and for N-acetylarginine (increased in healthy cats fed both fiber types but no effect in CKD cats).

In summary, important effects for the TCA cycle metabolites at baseline were that CKD cats had higher aconitate and lower succinate concentrations compared with healthy cats. In the urea cycle and the arginine and proline metabolism category, urea, citrulline, the dimethylarginnes (SDMA and ADMA), and N-acetylarginine concentrations were significantly higher in CKD cats compared with healthy cats at baseline and across all time points of the study. In CKD cats, urea concentrations decreased and argininate increased after consuming scFOS compared with baseline. Healthy cats had greater increases in citrate and alpha-ketoglutarate compared with CKD cats after consuming apple pomace, whereas CKD cats had increased succinate after consuming apple pomace. Urea concentrations increased in healthy cats, but decreased in CKD cats fed scFOS, whereas N-acetylarginine increased in healthy cats fed both fiber types, but had no effect in CKD cats.

Plasma metabolite concentrations for other renal-associated metabolites are shown in Table 7. At baseline, C-glycosyltryptophan, kynurenate, 5-hydroxyindoleacetate, 5-hydroxyindole sulfate, 7-hydroxyindole sulfate, 3-indoxyl-sulfate and pseudouridine concentrations were significantly higher in CKD cats compared with healthy cats. Across all time points of the study, based on overall effect of health (healthy cats versus CKD cats), there were eight significant differences: C-glycosyltryptophan, kynurenate, 5-hydroxyindoleacetate, 4-hydroxyhippurate, 4-acetylphenol sulfate, methyl-4-hydroxybenzoate sulfate, pseudouridine, and 3-aminoisobutyrate (BAIB) all had higher concentrations in CKD cats compared with healthy cats. Many metabolites in both healthy and CKD cats were affected by treatment with fiber (both scFOS and apple pomace), including guanidinoacetate in the creatine metabolites; tryptophan betaine, anthranilate, picolinate, indolelactate, indoleacetate, indoleacetylglutamine, and 5-bromotryptophan in the tryptophan metabolites; 3-hydroxyhippurate, 4-hydroxyhippurate, catechol sulfate, guaiacol sulfate, 4-ethylphenylsulfate, 4-vinylphenol sulfate, 3-methoxycatechol sulfate (1), 3-methoxycatechol sulfate (2), 3-(3-hydroxyphenyl)propionate sulfate, 3-(3-hydroxyphenyl)propionate, and 3-phenylpropionate (hydrocinnamate) in the benzoate metabolites; pseudouridine in the uracil derived pyrimidine metabolites; and BAIB in the thymine derived pyrimidine metabolites. Compared with baseline, healthy cats had higher concentrations of creatine (guanidinoacetate), tryptophan (C-glycosyltryptophan, anthranilate, 7-hydroxyindole sulfate), and uracil derived pyrimidine (pseudouridine) metabolites after consuming scFOS, or after consuming both fiber sources (tryptophan betaine, 5-bromotryptophan). Concentrations of the thymine derived pyrimidine metabolite BAIB were also decreased after consumption of both fiber sources in healthy cats. Tryptophan concentrations were lower after consuming scFOS. Only picolinate and indoleacetylglutamine had higher concentrations after consuming apple pomace. Compared with baseline, CKD cats had higher concentrations after consuming both fiber sources of guanidinoacetate, tryptophan betaine, and indoleacetate, and lower concentrations of N-acetyltryptophan. The CKD cats also had lower concentrations of 5-hydroxyindoleacetate and indolelactate after consuming scFOS, but higher concentrations of creatine and lower concentrations of pseudouridine after consuming apple pomace.

The benzoate metabolites, which contain many bacterial-generated phenolic nephrotoxins, had lower concentrations in healthy cats after consumption of both fiber sources [3-hydroxyhippurate, guaiacol sulfate, 3-(3-hydroxyphenyl)propionate sulfate, 3-(3-hydroxyphenyl)propionate] or after consuming scFOS [4-hydroxyhippurate, catechol sulfate, 3-methylcatechol sulfate (1), 3-(4-hydroxyphenyl)propionate]. Only 4-ethylphenylsulfate was increased after consumption of both fiber sources in healthy cats. Compared with baseline, after consumption of both fiber sources in CKD cats, only 3-hydroxyhippurate was decreased. After consumption of scFOS by CKD cats, guaiacol sulfate, 4-vinylphenol sulfate, 3-methoxycatechol sulfate (2), 3-(3-hydroxyphenyl)propionate sulfate, and 3-(3-hydroxyphenyl) propionate were decreased, whereas methyl-4-hydroxybenzoate sulfate was increased. After consumption of apple pomace by CKD cats, 4-ethylphenylsulfate and 3-phenylpropionate (hydrocinnamate) were increased.

There were no effects of treatment with apple pomace versus scFOS in healthy cats for renal-associated metabolites. In CKD cats, treatment with apple pomace increased creatine concentration more than scFOS, and treatment with scFOS decreased 4-vinylphenol sulfate more than treatment with apple pomace. The only overall effects of interaction were for creatine (CKD cats increased after consuming apple pomace and healthy cats had no change) and for BAIB (healthy cats decreased after both fiber treatments and CKD cats had no change).

In summary, in the category of other renal-associated metabolites, several metabolites were higher in CKD cats compared with healthy cats at baseline, including three indole sulfates, kynurenate, C-glycosyltryptophan, 5-hydroxyindoleacetate, and pseudouridine. In addition, across all time points, two more sulfates, BAIB, and 4-hydroxyhippurate concentrations were increased in CKD cats. These are plasma compounds produced by gut microbial metabolism. Evidence suggests these metabolites may have toxic effects on the host [33–37]. Many renal-associated metabolites were affected by consumption of dietary fiber (both scFOS and apple pomace) in both healthy and CKD cats. Overall effects of interaction were for creatine (CKD cats increased after consuming apple pomace and healthy cats had no change) and for BAIB (healthy cats decreased after both fiber treatments and CKD cats had no change). Beta-amino isobutyrate (BAIB) is formed in the cytosol from thymine degradation as the end product of pyrimidine metabolism. It is then transported into mitochondria and further metabolized to malonate semialdehyde by alanine-glyoxylate aminotransferase 2 (AGXT2). This enzyme is also known to catalyze the conversion of glyoxylate to glycine using L-alanine as the amino donor. Increased BAIB concentrations, noted in CKD cats in this study, may serve as an indicator of overall AGXT2 enzyme activity.

### Baseline and treatment differences in fecal microbiome based on health status of cats and type of fiber fed

Overall, 141 OTUs were evaluated (Table 6). In general, the OTUs in CKD cats were more resistant to change after feeding either fiber source (chi-square test, *P* < 0.05). This is in agreement with a study by Saggie et al. [38] which showed that the gut microbiome of human CKD patients is more resistant to change than healthy individuals. At baseline, genus *Sphingomonas* (OTU 1003206) was significantly higher in CKD cats compared with healthy cats. Four other OTUs, including an unclassified genus in the family f_S24-7 in the order Bacteroidales (OTU 100296), were significantly lower in CKD cats compared with healthy cats (S4 Table). The unclassified genus in f_S24-7-g_ (OTU 100296) was among the 9 OTUs that were significantly increased in CKD cats after consumption of food containing scFOS (S4 Table; *P* = 0.006). Linear regression analysis showed that this genus had significant negative correlations with several microbial uremic toxins: 3-indoxylsulfate (Pearson’s correlation coefficient, *r* = 0.39; *P* = 0.003), 5-hydroxyindole sulfate (*r* = 0.40; *P* = 0.002), 6-hydroxyindole sulfate (*r* = 0.39; *P* = 0.003), 7-hydroxyindole sulfate (*r* = 0.41; *P* = 0.001), 6-hydroxyindoleacetate (*r* = 0.33; *P* = 0.0098), hippurate (*r* = 0.36; *P* = 0.005) and 4-vinylguaiacol sulfate (*r* = 0.27; *P* = 0.047). None of the baseline differences in OTUs between healthy and CKD cats changed after CKD cats consumed food containing Apple pomace (S4 Table).

**Table 6:** OTU differences at baseline (BSL) and after feeding food A (containing scFOS) or food B (containing apple pomace) as fiber sources for 4 weeks each to healthy cats (H cats) and cats with chronic kidney disease (CKD).

	CKD vs H cats at BSL	H cats scFOS—BSL	H cats Apple pomace—BSL	H cats Apple pomace—scFOS	CKD cats scFOS—BSL	CKD cats Apple pomace—BSL	CKD cats Apple pomace—scFOS
**Total number of OTU significant at *P*<0.05**	5	41	46	17	22	17	7
**Number Increased**	1	24	29	6	9	2	2
**Number Decreased**	4	17	17	11	13	15	5

There may be a link between decreased abundance of bacterial members of the phylum Bacteroidetes and inflammatory diseases such as CKD. For example, decreased abundance of members of this phylum have been linked with obesity [39, 40] and other diseases including irritable bowel syndrome and CKD [41]. Mice with Alzheimer-like disease exhibit significant reduction in the abundance of bacteria in O_Bacteroidales_f_S24-7 (Park et al. 2017). Recent studies described the anti-inflammatory properties of bacteria in the Order Bacteroidales [42, 43]. In our study, the abundance of the genus increased when CKD cats were fed food containing scFOS. Takei et al [44] reported an increase in the abundance of f_S24-7 in mice fed fiber from edible algae. The negative correlations for the abundance of this genus with several circulating microbial uremic toxins suggest these bacterial members may have a role in decreasing levels of proteolytic microbial metabolite precursors of the uremic toxins.

The microbiome alpha diversity index for species richness showed that every measure of bacterial diversity decreased in healthy cats from baseline after consuming either type of fiber (Table 7). In CKD cats, microbiome abundance evenness (J) and the omnibus indices (expH, invSimp) decreased whereas species richness was unchanged by fiber consumption. Thus, although evenness and overall measures of diversity responded similarly in healthy and CKD cats, healthy cats showed a loss of species richness with fiber intake whereas the CKD cat microbiome was resistant to change. This resulted in the appearance of a significantly less species richness in healthy cats compared to CKD cats when consuming fiber treatments, but not at baseline. Although a more diverse microbiome is presumed advantageous, as evidenced in this study, healthy benefits are not based entirely on having greater diversity. Prebiotics like scFOS may be beneficial even though diversity decreases as a result of added fiber that promotes the growth of saccharolytic bacteria. Each cat received all three diets, thus minimizing individual variation in microbiota.

**Table 7:** Microbiome alpha-diversity indices in healthy cats (H cats) and chronic kidney disease (CKD) cats at baseline (after feeding pretrial food^1^ for 14 days) and after feeding food A^2^ (containing scFOS) or food B^3^ (containing apple pomace) as fiber sources for 4 weeks each.

	Mean Values						Group Effects^‡^				
	H Cats			CKD Cats			Overall Effect of Health	Overall Effect of Both Treatments	H Cats: Treatment with Food B vs Food A^¥^	CKD Cats: Treatment with Food B vs Food A^¥^	Overall effect of interaction
Alpha-diversity indices	BSL	Food A	Food B	BSL	Food A	Food B	*P*-value	*P*-value	*P*-value	*P*-value	*P*-value
S: species richness	69.00	63.20^§^^,^^#^	59.70^§^^,^^#^	69.80	73.20^#^	71.89^#^	0.01	0.20	0.20	0.81	0.01
expH: exponential of Shannon Index	11.47	8.22^§^	7.55^§^	13.20	9.28^§^	8.97^§^	0.18	< 0.001	0.38	0.95	0.82
invSimp: inverse of Simpson Index	7.32	5.15^§^	4.89^§^	8.50	5.42^§^	5.24^§^	0.35	< 0.001	0.59	0.99	0.51
J: evenness distribution	0.57	0.50^§^	0.49^§^	0.60	0.51^§^	0.50^§^	0.41	< 0.001	0.65	0.96	0.79

^**1–3, ‡**, ¥, #^ See Table 3.

^§^The mixed model was used to test whether the difference of two paired observations from a single cat at baseline and the end of the 4 week feeding period was different than zero.

Significance was established when *P* ≤ 0.05 (for type 1 error).

Barry et al. [45] previously reported that the number of *Bifidobacterium* spp. increased and *Escherichia coli* decreased in the feces of 4% FOS-supplemented cats, whereas in 4% pectin-supplemented cats the proportion of *Clostridium perfringens*, *E*. *coli*, and *Lactobacillus* spp increased. None-the-less, 4% supplemental fiber did not result in large-scale shifts of the gastrointestinal microbiota [46] in those studies, similar to what we found in this study. Pallotto et al. [47] reported that in obese cats, high-fiber diets of up to 16.8% (DM basis) have been fed to effect weight loss, and the relative abundance of Actinobacteria increased and Bacteroidetes decreased with weight loss; alpha diversity was not affected by weight loss. Even *in vitro* studies with feline fecal microbiota show that different prebiotics (including FOS and pectins from citrus fruit) exert different effects on the composition and activity of feline intestinal microbiota [48]. Overall, the data suggest the need to select fiber sources for CKD cats that overcome not only the resistance of the gut microbiome to change, but also that promote favorable changes to the microbiome.

## Conclusions

The main finding of this study was that the health status of cats influenced the effects of dietary fermentable fibers. Variance in plasma metabolites was greater for CKD cats compared with healthy cats at baseline and after feeding both fiber sources for 4 weeks. At baseline, cats with CKD had increased plasma concentrations of creatinine, urea, and microbial and host tryptophan metabolites, including several hydroxyindole sulfates. After feeding both types of fiber, metabolomics data showed that cats with CKD had higher concentrations of the more oxidized glutathione metabolites (e.g., GSSG and cysteine-glutathione disulfide) after consuming apple pomace compared with scFOS, as well as higher concentrations of inflammatory sphingolipid metabolites (e.g., sphingosine-1-phosphate) after consuming apple pomace, but not scFOS. After consuming scFOS, CKD cats had lower concentrations of phenolic uremic toxins (e.g., guaiacol sulfate and 4-vinylphenol sulfate) compared with after consuming apple pomace. Conversely, healthy cats had higher concentration of antioxidants (e.g., α-tocopherol) after consuming apple pomace; and higher concentrations of inflammatory sphingolipid metabolites after consuming scFOS, but not after consuming apple pomace. The fecal microbiome showed five significant microbial OTU differences in CKD cats compared with healthy cats, but overall, the OTUs in CKD cats were more resistant to change after feeding either fiber source. There was evidence that feeding CKD cats scFOS, but not apple pomace, was helpful for increasing counts of an unclassified genus in the family S24-7 of the order *Bacteroidales* (OTU 100296), which was negatively correlated with several microbial uremic toxins (e.g., 3-indoxylsulfate). These results suggest the importance of choosing fiber sources based on health status to maximize antioxidant status and minimize inflammatory sphingolipid metabolites, as well as to enrich beneficial bacterial members. A more readily fermented fiber such as scFOS is preferable to apple pomace as a fiber source for cats with CKD, whereas healthy cats had favorable changes when fed apple pomace as a fiber source.

## Supporting information

S1 Table (XLSX)

S2 Table (DOCX)

S3 Table (DOCX)

S4 Table (DOCX)

## Abbreviations

ADMA: asymmetric dimethylarginine
AGXT2: alanine-glyoxylate aminotransferase 2
BAIB: beta-amino isobutyrate
BSL: baseline
BUN: blood urea nitrogen
CKD: chronic kidney disease
CLR: centered log-ratio
Cr: creatinine
expH: exponential of Shannon Index
FA: fatty acids
GSSG: oxidized glutathione
H cats: healthy cats
invSimp: inverse of the Simpson index
J: evenness distribution
MUFA: monounsaturated fatty acids
OTU: operational taxonomic units
PC: principal components
PCA: principal component analysis
PCR: polymerase chain reaction
PUFA: polyunsaturated fatty acids
S: species richness
SAH: S-adenosylhomocystein
scFOS: short chain fructooligosaccharides
SDMA: symmetric dimethylarginine
SFA: saturated fatty acids
TCA: tricarboxylic acid cycle
USG: urine specific gravity
