# Supplementary material for: Chronic kidney disease in cats alters response of the plasma metabolome and fecal microbiome to dietary fiber
Source: PLoS One. 2020 Jul 2;15(7):e0235480. doi: 10.1371/journal.pone.0235480 (PMC7331996; doi:10.1371/journal.pone.0235480)
Supplement: S2 Table — (DOCX) [file pone.0235480.s002.docx]

**Table S2.** Eigenvector metabolites that contributed to separation of the two groups in Figure 1A, PC1 and PC2.

| **Figure 1A** | **PC1** |  | **Figure 1A** | **PC2** |
| --- | --- | --- | --- | --- |
| creatine | -0.05519 |  | imidazole lactate | -0.06961 |
| tryptophan | -0.05405 |  | linoleoyl-linoleoyl-glycerol (18:2/18:2) [1]* | -0.06082 |
| nervonate (24:1n9)* | -0.05172 |  | arginine | -0.05898 |
| behenate (22:0)* | -0.05045 |  | leucine | -0.05889 |
| 2'-deoxycytidine | -0.05034 |  | valine | -0.05866 |
| adenine | -0.04977 |  | AMP | -0.05738 |
| fructose | -0.04886 |  | isoleucine | -0.05688 |
| glycerophosphorylcholine (GPC) | -0.04806 |  | 5-aminovalerate | -0.05512 |
| nicotinamide | -0.04704 |  | carnosine | -0.05453 |
| glycerophosphoethanolamine | -0.04685 |  | pyroglutamine* | -0.0543 |
| mannose | -0.04646 |  | threonine | -0.05327 |
| guanine | -0.04603 |  | homoarginine | -0.05221 |
| 10-undecenoate (11:1n1) | -0.04577 |  | guanidinoacetate | -0.05149 |
| 5-bromotryptophan | -0.04558 |  | felinine | -0.05114 |
| bilirubin (E,Z or Z,E)* | -0.0455 |  | 1-stearoyl-2-docosahexaenoyl-GPC (18:0/22:6) | -0.05108 |
| eicosanodioate (C20-DC) | -0.04546 |  | dodecadienoate (12:2)* | -0.0501 |
| kynurenine | -0.0444 |  | 1-palmitoyl-2-docosahexaenoyl-GPE (16:0/22:6)* | -0.0496 |
| nicotinate ribonucleoside | -0.04437 |  | 5alpha-pregnan-3beta,20beta-diol monosulfate (1) | -0.04915 |
| docosadioate (C22-DC) | -0.04432 |  | inosine | -0.04864 |
| 2-hydroxystearate | -0.0433 |  | beta-alanine | -0.04831 |
| bilirubin | -0.04298 |  | alanine | -0.04755 |
| biliverdin | -0.04182 |  | N-acetylglycine | -0.04518 |
| 1-linoleoyl-GPI* (18:2)* | -0.04099 |  | 10-undecenoate (11:1n1) | -0.04514 |
| 1-(1-enyl-stearoyl)-2-linoleoyl-GPE (P-18:0/18:2)* | -0.04083 |  | prolylhydroxyproline | -0.04508 |
| N2,N2-dimethylguanosine | -0.0408 |  | 12-HETE | -0.0446 |
| 1-linolenoyl-GPC (18:3)* | -0.04077 |  | succinate | -0.04436 |
| 1-stearoyl-2-linoleoyl-GPC (18:0/18:2)* | -0.04064 |  | serotonin | -0.04322 |
| 1-palmitoyl-2-stearoyl-GPC (16:0/18:0) | -0.04056 |  | N-linolenoyltaurine* | -0.04311 |
| tyrosine | -0.04053 |  | 12-HHTrE | -0.04255 |
| 1-stearoyl-GPI (18:0) | -0.04036 |  | 1-palmitoyl-2-docosahexaenoyl-GPC (16:0/22:6) | -0.04158 |
| palmitoylcholine | -0.04029 |  | docosapentaenoate (DPA; 22:5n3) | -0.04083 |
| thymol sulfate | -0.04001 |  | 3-hydroxymyristate | -0.04063 |
| 1-stearoyl-GPC (18:0) | -0.03999 |  | hypoxanthine | -0.03978 |
| 1-palmitoyl-2-alpha-linolenoyl-GPC (16:0/18:3n3)* | -0.03939 |  | guanosine | -0.03896 |
| phenylalanine | -0.03916 |  | oleoyl-linoleoyl-glycerol (18:1/18:2) [1] | -0.03881 |
| 1-linoleoyl-GPC (18:2) | -0.03894 |  | deoxycarnitine | -0.03869 |
| heme | -0.03883 |  | dihomolinolenate (20:3n3 or 3n6) | -0.03851 |
| 1-palmitoyl-GPC (16:0) | -0.03868 |  | heneicosapentaenoate (21:5n3) | -0.03834 |
| octadecenedioate (C18:1-DC)* | -0.03839 |  | palmitoleoyl-linoleoyl-glycerol (16:1/18:2) [1]* | -0.03832 |
| 1-oleoyl-GPI (18:1)* | -0.03834 |  | sulfate* | -0.03724 |
| 1-palmitoyl-GPI* (16:0) | -0.03834 |  | eicosanodioate (C20-DC) | -0.03723 |
| alpha-tocopherol | -0.03818 |  | 1-stearoyl-2-linoleoyl-GPS (18:0/18:2) | -0.03686 |
| 1-arachidonoyl-GPI* (20:4)* | -0.03813 |  | N-monomethylarginine | -0.03669 |
| caproate (6:0) | -0.0372 |  | erucate (22:1n9) | -0.03664 |
| 1-stearoyl-2-arachidonoyl-GPC (18:0/20:4) | -0.03717 |  | 3-hydroxybutyrate (BHBA) | -0.03641 |
| arachidonate (20:4n6) | -0.03679 |  | stearidonate (18:4n3) | -0.03641 |
| linoleoylcholine* | -0.03665 |  | arachidonate (20:4n6) | -0.03625 |
| 2-hydroxypalmitate | -0.03639 |  | linoleoyl-linolenoyl-glycerol (18:2/18:3) [2]* | -0.03588 |
| campesterol | -0.0363 |  | eicosapentaenoate (EPA; 20:5n3) | -0.03555 |
| glucose | -0.03627 |  | prolylglycine | -0.03546 |
| 1-(1-enyl-palmitoyl)-GPE (P-16:0)* | -0.03609 |  | ornithine | -0.03469 |
| 1-arachidonoyl-GPC* (20:4)* | -0.03603 |  | 2-oxoarginine* | -0.03412 |
| 2-hydroxynervonate* | -0.03599 |  | docosadienoate (22:2n6) | -0.03404 |
| 1-(1-enyl-stearoyl)-2-oleoyl-GPE (P-18:0/18:1) | -0.03573 |  | docosadioate (C22-DC) | -0.03357 |
| bilirubin (E,E)* | -0.0356 |  | hexadecatrienoate (16:3n3) | -0.03343 |
| beta-sitosterol | -0.03537 |  | linolenate (18:3n3 or 3n6) | -0.03336 |
| 1-(1-enyl-stearoyl)-GPE (P-18:0)* | -0.03532 |  | N-acetyl-aspartyl-glutamate (NAAG) | -0.03308 |
| 1-(1-enyl-palmitoyl)-2-oleoyl-GPE (P-16:0/18:1)* | -0.03493 |  | gamma-glutamylfelinylglycine* | -0.03279 |
| methylnaphthyl sulfate (1)* | -0.03491 |  | docosahexaenoate (DHA; 22:6n3) | -0.03264 |
| valerate (5:0) | -0.03488 |  | asparagine | -0.03259 |
| N-palmitoyl-sphinganine (d18:0/16:0) | -0.03414 |  | anserine | -0.03216 |
| beta-alanine | -0.03408 |  | 4-hydroxyphenylpyruvate | -0.03183 |
| 1-(1-enyl-palmitoyl)-GPC (P-16:0)* | -0.03405 |  | docosatrienoate (22:3n3) | -0.03182 |
| gluconate | -0.03377 |  | eicosenoate (20:1n9 or 1n11) | -0.03181 |
| sphingomyelin (d18:2/14:0, d18:1/14:1)* | -0.03354 |  | dihydroorotate | -0.03156 |
| sphinganine-1-phosphate | -0.03282 |  | gamma-glutamylthreonine | -0.03154 |
| methionine | -0.03227 |  | dihomolinoleate (20:2n6) | -0.03149 |
| 1-stearoyl-2-arachidonoyl-GPS (18:0/20:4) | -0.0321 |  | histidine | -0.03148 |
| carotene diol (3) | -0.03203 |  | thromboxane B2 | -0.03144 |
| carotene diol (1) | -0.03202 |  | imidazole propionate | -0.03143 |
| thioproline | -0.03189 |  | cytidine | -0.0314 |
| 1-oleoyl-GPC (18:1) | -0.03164 |  | gamma-glutamylvaline | -0.03089 |
| aspartate | -0.03149 |  | 2-aminobutyrate | -0.03083 |
| thymidine | -0.03136 |  | N-glycolylneuraminate | -0.0302 |
| linoleoyl-arachidonoyl-glycerol (18:2/20:4) [2]* | -0.03101 |  | 1-stearoyl-2-oleoyl-GPS (18:0/18:1) | -0.02983 |
| 1-palmitoleoyl-2-linolenoyl-GPC (16:1/18:3)* | -0.03093 |  | alpha-hydroxyisovalerate | -0.0296 |
| 2'-deoxyuridine | -0.03079 |  | N-myristoyltaurine* | -0.02957 |
| 1,2-dipalmitoyl-GPC (16:0/16:0) | -0.03071 |  | 15-methylpalmitate (i17:0) | -0.02952 |
| glutamate | -0.0306 |  | indolelactate | -0.02925 |
| laurate (12:0) | -0.03059 |  | 4-methyl-2-oxopentanoate | -0.02913 |
| 1-(1-enyl-oleoyl)-GPE (P-18:1)* | -0.03034 |  | 3-hydroxylaurate | -0.02893 |
| oleoyl-arachidonoyl-glycerol (18:1/20:4) [2]* | -0.02988 |  | gamma-glutamylglutamine | -0.02878 |
| octadecanedioate (C18) | -0.02973 |  | S-methylcysteine sulfoxide | -0.02832 |
| diacylglycerol (16:1/18:2 [2], 16:0/18:3 [1])* | -0.02971 |  | 2-aminoadipate | -0.02805 |
| diacylglycerol (14:0/18:1, 16:0/16:1) [2]* | -0.02966 |  | alpha-ketobutyrate | -0.02768 |
| dihomolinolenate (20:3n3 or 3n6) | -0.02957 |  | 1-stearoyl-2-arachidonoyl-GPE (18:0/20:4) | -0.02745 |
| 1-myristoyl-2-arachidonoyl-GPC (14:0/20:4)* | -0.02956 |  | N-palmitoyltaurine | -0.02739 |
| 5-methyl-2'-deoxycytidine | -0.02947 |  | adrenate (22:4n6) | -0.02689 |
| 1-oleoyl-2-linoleoyl-GPC (18:1/18:2)* | -0.02942 |  | 2-hydroxy-3-methylvalerate | -0.02668 |
| cortisol | -0.02929 |  | gamma-glutamylleucine | -0.02649 |
| oleoylcholine | -0.02912 |  | 3-methyl-2-oxovalerate | -0.02645 |
| sphingosine 1-phosphate | -0.02894 |  | glutamine | -0.02633 |
| linoleoyl-arachidonoyl-glycerol (18:2/20:4) [1]* | -0.02864 |  | 5-dodecenoate (12:1n7) | -0.02619 |
| 1-palmitoyl-2-arachidonoyl-GPC (16:0/20:4n6) | -0.02853 |  | hexadecadienoate (16:2n6) | -0.02587 |
| 1-myristoyl-2-palmitoyl-GPC (14:0/16:0) | -0.02829 |  | myristoleate (14:1n5) | -0.02554 |
| 1,2-dilinoleoyl-GPC (18:2/18:2) | -0.02816 |  | 2-aminooctanoate | -0.02522 |
| behenoyl dihydrosphingomyelin (d18:0/22:0)* | -0.02778 |  | orotate | -0.02519 |
| palmitoyl-linoleoyl-glycerol (16:0/18:2) [1]* | -0.02739 |  | methylnaphthyl sulfate (1)* | -0.02513 |
| palmitoyl dihydrosphingomyelin (d18:0/16:0)* | -0.02722 |  | malonate | -0.02478 |
| myristoyl dihydrosphingomyelin (d18:0/14:0)* | -0.02702 |  | phenylpyruvate | -0.02472 |
| sphingomyelin (d18:1/14:0, d16:1/16:0)* | -0.02701 |  | stearate (18:0) | -0.02423 |
| 1-stearoyl-2-oleoyl-GPS (18:0/18:1) | -0.02689 |  | 4-hydroxyphenylacetylglycine | -0.0242 |
| arachidonoylcholine | -0.02651 |  | 10-nonadecenoate (19:1n9) | -0.02408 |
| docosadienoate (22:2n6) | -0.0265 |  | 4-guanidinobutanoate | -0.02377 |
| carotene diol (2) | -0.0265 |  | felinylglycine* | -0.0235 |
| isovalerate (C5) | -0.02649 |  | gamma-glutamylalanine | -0.02337 |
| 1-stearoyl-2-oleoyl-GPC (18:0/18:1) | -0.02648 |  | 3-hydroxydecanoate | -0.02334 |
| 2-hydroxylaurate | -0.02646 |  | thyroxine | -0.02321 |
| stearoylcholine* | -0.02621 |  | 3-methyl-2-oxobutyrate | -0.02312 |
| 2-palmitoyl-GPC* (16:0)* | -0.02618 |  | N-linoleoyltaurine* | -0.02308 |
| ethylmalonate | -0.026 |  | laurate (12:0) | -0.0228 |
| S-methylcysteine | -0.02587 |  | margarate (17:0) | -0.02277 |
| gamma-tocopherol/beta-tocopherol | -0.02566 |  | 3-(4-hydroxyphenyl)lactate (HPLA) | -0.02254 |
| sebacate (C10-DC) | -0.02561 |  | 13-HODE + 9-HODE | -0.02251 |
| erucate (22:1n9) | -0.02559 |  | hexanoylglycine (C6) | -0.02229 |
| 1-stearoyl-2-oleoyl-GPE (18:0/18:1) | -0.02552 |  | inosine 5'-monophosphate (IMP) | -0.02174 |
| 3-methoxytyrosine | -0.02528 |  | S-adenosylhomocysteine (SAH) | -0.02167 |
| guanidinoacetate | -0.02521 |  | 6-oxopiperidine-2-carboxylate | -0.02149 |
| xanthine | -0.0251 |  | 4-hydroxyphenylacetate | -0.02143 |
| 1-linoleoyl-2-linolenoyl-GPC (18:2/18:3)* | -0.02488 |  | N-acetyl-cadaverine | -0.02121 |
| 15-methylpalmitate (i17:0) | -0.02477 |  | caprate (10:0) | -0.02088 |
| 1-(1-enyl-stearoyl)-2-arachidonoyl-GPE (P-18:0/20:4)* | -0.02471 |  | ectoine | -0.02048 |
| 1-palmitoyl-2-palmitoleoyl-GPC (16:0/16:1)* | -0.02462 |  | iminodiacetate (IDA) | -0.02029 |
| serine | -0.0242 |  | glycine | -0.02017 |
| uridine | -0.02419 |  | maleate | -0.02016 |
| 5-hydroxymethyl-2'-deoxycytidine | -0.02409 |  | cysteine sulfinic acid | -0.01986 |
| 1-lignoceroyl-GPC (24:0) | -0.02406 |  | palmitoleate (16:1n7) | -0.01981 |
| N-behenoyl-sphingadienine (d18:2/22:0)* | -0.02369 |  | 5-hydroxymethylcytidine | -0.01971 |
| sphingomyelin (d18:0/20:0, d16:0/22:0)* | -0.02367 |  | linoleate (18:2n6) | -0.01952 |
| tryptophan betaine | -0.02362 |  | nonadecanoate (19:0) | -0.01946 |
| diacylglycerol (14:0/18:1, 16:0/16:1) [1]* | -0.0236 |  | gamma-glutamylisoleucine* | -0.01944 |
| 17-methylstearate (i19:0) | -0.02358 |  | N-acetylhistamine | -0.01922 |
| 1-palmitoyl-2-linoleoyl-GPC (16:0/18:2) | -0.02345 |  | oleoyl-linoleoyl-glycerol (18:1/18:2) [2] | -0.01919 |
| 1-palmitoleoyl-GPC* (16:1)* | -0.02335 |  | 1-methylhistamine | -0.01915 |
| 1-linoleoyl-2-arachidonoyl-GPC (18:2/20:4n6)* | -0.0233 |  | eugenol sulfate | -0.0191 |
| 1-stearoyl-2-oleoyl-GPI (18:0/18:1)* | -0.02318 |  | proline | -0.01867 |
| hypoxanthine | -0.02311 |  | stearoyl-linoleoyl-glycerol (18:0/18:2) [2]* | -0.01847 |
| palmitoyl-linoleoyl-glycerol (16:0/18:2) [2]* | -0.02307 |  | 3-phosphoglycerate | -0.01838 |
| 3-phenylpropionate (hydrocinnamate) | -0.02254 |  | cis-4-decenoylcarnitine (C10:1) | -0.01818 |
| ceramide (d18:1/14:0, d16:1/16:0)* | -0.02246 |  | dimethylarginine (ADMA + SDMA) | -0.01816 |
| sphingadienine | -0.02246 |  | 3-hydroxyhexanoate | -0.01816 |
| leucine | -0.02239 |  | homostachydrine* | -0.01805 |
| 1-stearoyl-2-linoleoyl-GPS (18:0/18:2) | -0.02235 |  | propionylglycine (C3) | -0.0178 |
| choline | -0.02224 |  | uridine | -0.01763 |
| 1-linoleoylglycerol (18:2) | -0.02157 |  | linoleoyl-arachidonoyl-glycerol (18:2/20:4) [1]* | -0.01745 |
| arachidate (20:0) | -0.02115 |  | linolenoylcarnitine (C18:3)* | -0.0174 |
| 1-palmitoyl-2-oleoyl-GPI (16:0/18:1)* | -0.02101 |  | palmitoyl-linoleoyl-glycerol (16:0/18:2) [1]* | -0.01731 |
| linoleoyl-linolenoyl-glycerol (18:2/18:3) [2]* | -0.02094 |  | myristate (14:0) | -0.01712 |
| 2,4-dichlorophenol sulfate | -0.02082 |  | 3-methoxycatechol sulfate (1) | -0.01697 |
| ceramide (d18:2/24:1, d18:1/24:2)* | -0.02082 |  | palmitate (16:0) | -0.01647 |
| 12,13-DiHOME | -0.02075 |  | 4-hydroxyphenylacetate sulfate | -0.01615 |
| palmitoleoyl-linoleoyl-glycerol (16:1/18:2) [1]* | -0.02069 |  | palmitoylcarnitine (C16) | -0.01565 |
| N-stearoylserine* | -0.02058 |  | 5-hydroxylysine | -0.01549 |
| adrenate (22:4n6) | -0.02044 |  | mevalonolactone | -0.01503 |
| sphingomyelin (d18:2/21:0, d16:2/23:0)* | -0.02036 |  | oleoyl-oleoyl-glycerol (18:1/18:1) [1]* | -0.01491 |
| nonadecanoate (19:0) | -0.02022 |  | tyrosine | -0.01479 |
| hexadecanedioate (C16) | -0.02001 |  | aspartate | -0.01472 |
| stearoyl-arachidonoyl-glycerol (18:0/20:4) [2]* | -0.01981 |  | N6-acetyllysine | -0.0147 |
| isoleucine | -0.01972 |  | N6-methyladenosine | -0.01469 |
| heptanoate (7:0) | -0.01971 |  | N4-acetylcytidine | -0.01446 |
| docosapentaenoate (n6 DPA; 22:5n6) | -0.01967 |  | octanoylcarnitine (C8) | -0.01438 |
| linolenoylcarnitine (C18:3)* | -0.0196 |  | malate | -0.01415 |
| sphingomyelin (d17:1/14:0, d16:1/15:0)* | -0.01953 |  | creatine | -0.01408 |
| palmitoyl-oleoyl-glycerol (16:0/18:1) [2]* | -0.01943 |  | lysine | -0.01406 |
| asparagine | -0.01938 |  | thymol sulfate | -0.01362 |
| linoleoyl ethanolamide | -0.01914 |  | 2-hydroxyphenylacetate | -0.01361 |
| lignoceroyl sphingomyelin (d18:1/24:0) | -0.01908 |  | phenyllactate (PLA) | -0.01358 |
| 1-palmitoyl-2-linoleoyl-GPI (16:0/18:2) | -0.01841 |  | docosatrienoate (22:3n6)* | -0.01344 |
| 4-methyl-2-oxopentanoate | -0.01839 |  | oleate/vaccenate (18:1) | -0.0134 |
| guanosine | -0.01821 |  | phosphate | -0.01324 |
| linolenate (18:3n3 or 3n6) | -0.01821 |  | tryptophan betaine | -0.01294 |
| cysteine sulfinic acid | -0.01802 |  | thymidine | -0.01279 |
| sedoheptulose | -0.01769 |  | pentadecanoate (15:0) | -0.01256 |
| ergothioneine | -0.01759 |  | gamma-glutamylglycine | -0.01245 |
| stearoyl-linoleoyl-glycerol (18:0/18:2) [2]* | -0.01742 |  | tryptophan | -0.0123 |
| taurocholenate sulfate | -0.01739 |  | 1-stearoyl-2-arachidonoyl-GPC (18:0/20:4) | -0.01201 |
| dopamine 3-O-sulfate | -0.01736 |  | 3-hydroxyisobutyrate | -0.01181 |
| 2-hydroxydecanoate | -0.01723 |  | 10-heptadecenoate (17:1n7) | -0.01177 |
| docosahexaenoate (DHA; 22:6n3) | -0.0172 |  | glutaroylcarnitine (C5) | -0.01169 |
| docosatrienoate (22:3n6)* | -0.01715 |  | cysteine-glutathione disulfide | -0.01139 |
| phosphoethanolamine (PE) | -0.01667 |  | oleoyl-oleoyl-glycerol (18:1/18:1) [2]* | -0.01138 |
| sphingomyelin (d18:1/21:0, d17:1/22:0, d16:1/23:0)* | -0.01666 |  | carnitine | -0.01134 |
| cholesterol | -0.01643 |  | O-sulfo-L-tyrosine | -0.01124 |
| sphingomyelin (d18:1/25:0, d19:0/24:1, d20:1/23:0, d19:1/24:0)* | -0.01638 |  | (N(1) + N(8))-acetylspermidine | -0.01115 |
| ferulic acid 4-sulfate | -0.01632 |  | 4-imidazoleacetate | -0.01109 |
| chenodeoxycholate | -0.01588 |  | docosapentaenoate (n6 DPA; 22:5n6) | -0.01083 |
| 1-oleoyl-GPE (18:1) | -0.01561 |  | isovalerylglycine | -0.01066 |
| oleoyl-linoleoyl-glycerol (18:1/18:2) [2] | -0.01541 |  | methylnaphthyl sulfate (2)* | -0.01062 |
| N-palmitoyl-sphingosine (d18:1/16:0) | -0.01532 |  | xylose | -0.01045 |
| ceramide (d16:1/24:1, d18:1/22:1)* | -0.01531 |  | arachidate (20:0) | -0.01005 |
| inosine 5'-monophosphate (IMP) | -0.01521 |  | diacylglycerol (16:1/18:2 [2], 16:0/18:3 [1])* | -0.00997 |
| cholate | -0.01511 |  | myristoylcarnitine (C14) | -0.00941 |
| tricosanoyl sphingomyelin (d18:1/23:0)* | -0.0151 |  | retinol (Vitamin A) | -0.00932 |
| behenoyl sphingomyelin (d18:1/22:0)* | -0.01501 |  | bilirubin (E,E)* | -0.00931 |
| palmitoyl sphingomyelin (d18:1/16:0) | -0.01491 |  | S-1-pyrroline-5-carboxylate | -0.00925 |
| sphingomyelin (d18:2/18:1)* | -0.01485 |  | adenine | -0.00917 |
| betaine | -0.01455 |  | lactate | -0.00906 |
| glycerophosphoinositol* | -0.0145 |  | pipecolate | -0.00881 |
| taurodeoxycholate | -0.01444 |  | glutamate | -0.00847 |
| lignoceroylcarnitine (C24)* | -0.01443 |  | pyruvate | -0.00839 |
| ximenoylcarnitine (C26:1)* | -0.01439 |  | dimethylglycine | -0.00827 |
| N-linolenoyltaurine* | -0.01413 |  | 2-stearoyl-GPE (18:0)* | -0.00822 |
| 1-stearoyl-GPE (18:0) | -0.0141 |  | mevalonate | -0.00805 |
| 1-linoleoyl-2-arachidonoyl-GPE (18:2/20:4)* | -0.01401 |  | N-acetylcarnosine | -0.008 |
| palmitoyl-oleoyl-glycerol (16:0/18:1) [1]* | -0.0139 |  | 4-vinylguaiacol sulfate | -0.00788 |
| 9,10-DiHOME | -0.01389 |  | 2-piperidinone | -0.00768 |
| sphingomyelin (d18:2/23:1)* | -0.01377 |  | serine | -0.00763 |
| sphingomyelin (d18:1/22:1, d18:2/22:0, d16:1/24:1)* | -0.01364 |  | 2-hydroxylaurate | -0.0074 |
| 1-(1-enyl-palmitoyl)-2-arachidonoyl-GPC (P-16:0/20:4)* | -0.01361 |  | 1,5-anhydroglucitol (1,5-AG) | -0.00732 |
| 2-aminoheptanoate | -0.01347 |  | alpha-hydroxyisocaproate | -0.00701 |
| 1-stearoyl-2-linoleoyl-GPE (18:0/18:2)* | -0.01331 |  | 3-hydroxyoctanoate | -0.00687 |
| sphingomyelin (d18:2/23:0, d18:1/23:1, d17:1/24:1)* | -0.0133 |  | 2-hydroxyglutarate | -0.00637 |
| 3-(3-hydroxyphenyl)propionate | -0.0133 |  | N-palmitoylglycine | -0.0063 |
| serotonin | -0.01323 |  | 1-palmitoyl-2-arachidonoyl-GPE (16:0/20:4)* | -0.006 |
| sphinganine | -0.0132 |  | S-methylcysteine | -0.00598 |
| deoxycholate | -0.01318 |  | linoleoylcarnitine (C18:2)* | -0.00586 |
| 1-stearoyl-2-linoleoyl-GPI (18:0/18:2) | -0.01305 |  | ophthalmate | -0.0055 |
| 3-methyl-2-oxovalerate | -0.01293 |  | 1-linoleoyl-GPA (18:2)* | -0.00545 |
| stearoyl-arachidonoyl-glycerol (18:0/20:4) [1]* | -0.01278 |  | phenylacetylserine | -0.00534 |
| sphingomyelin (d18:0/18:0, d19:0/17:0)* | -0.01243 |  | 17-methylstearate (i19:0) | -0.00524 |
| N-stearoyl-sphinganine (d18:0/18:0)* | -0.01227 |  | homocystine | -0.0051 |
| thromboxane B2 | -0.01221 |  | 2-methylserine | -0.00508 |
| pantothenate (Vitamin B5) | -0.01213 |  | 2-hydroxybutyrate/2-hydroxyisobutyrate | -0.00477 |
| taurocholate | -0.01184 |  | creatinine | -0.00474 |
| taurochenodeoxycholate | -0.01173 |  | spermidine | -0.00462 |
| oleoyl-linoleoyl-glycerol (18:1/18:2) [1] | -0.01171 |  | adenosine | -0.00438 |
| inosine | -0.0116 |  | N-oleoyltaurine | -0.00432 |
| taurolithocholate 3-sulfate | -0.01158 |  | cysteine | -0.00391 |
| 12-HHTrE | -0.01155 |  | N-palmitoleoyltaurine* | -0.00352 |
| arachidonoylcarnitine (C20:4) | -0.01153 |  | homocitrulline | -0.0034 |
| 2-aminooctanoate | -0.01153 |  | 12,13-DiHOME | -0.0033 |
| alpha-CEHC sulfate | -0.01149 |  | nervonate (24:1n9)* | -0.0029 |
| methyl-4-hydroxybenzoate sulfate | -0.01137 |  | guanine | -0.00268 |
| 1-oleoylglycerol (18:1) | -0.01132 |  | arabitol/xylitol | -0.00265 |
| thyroxine | -0.01128 |  | alpha-ketoglutaramate | -0.00255 |
| adrenoylcarnitine (C22:4)* | -0.01123 |  | arachidonoylcarnitine (C20:4) | -0.00234 |
| N-palmitoyl-sphingadienine (d18:2/16:0)* | -0.01122 |  | 2-oxoadipate | -0.00229 |
| phenylpropionylglycine | -0.01118 |  | hippurate | -0.00226 |
| AMP | -0.01096 |  | allantoic acid | -0.00207 |
| salicylate | -0.01087 |  | phenylpropionylglycine | -0.00191 |
| 1-stearoyl-2-arachidonoyl-GPI (18:0/20:4) | -0.01087 |  | gamma-tocopherol/beta-tocopherol | -0.00163 |
| spermidine | -0.01082 |  | phenylacetylglycine | -0.00157 |
| indoleacetate | -0.01077 |  | 1-oleoyl-2-docosahexaenoyl-GPC (18:1/22:6)* | -0.00147 |
| ceramide (d18:1/20:0, d16:1/22:0, d20:1/18:0)* | -0.01077 |  | 2-hydroxynervonate* | -0.00125 |
| ceramide (d18:1/17:0, d17:1/18:0)* | -0.0107 |  | salicylate | -0.00116 |
| 4-imidazoleacetate | -0.01068 |  | hydroxyproline | -0.0009 |
| hexadecenedioate (C16:1-DC)* | -0.01053 |  | 1-methylhistidine | -0.00089 |
| 5-dodecenoate (12:1n7) | -0.01028 |  | glycerol | -0.00063 |
| eicosapentaenoate (EPA; 20:5n3) | -0.01004 |  | arachidonoylcholine | -0.00052 |
| sphingomyelin (d17:1/16:0, d18:1/15:0, d16:1/17:0)* | -0.00993 |  | ethylmalonate | -0.00046 |
| 12-HETE | -0.00946 |  | palmitoleoylcarnitine (C16:1)* | -0.00045 |
| N-stearoyltaurine | -0.00924 |  | butyrylglycine (C4) | -0.00026 |
| hippurate | -0.0092 |  | argininate* | 0.00001 |
| 1-methylhistamine | -0.00911 |  | gamma-glutamylglutamate | 0.00006 |
| sphingomyelin (d18:2/24:2)* | -0.00895 |  | glycerophosphoinositol* | 0.00013 |
| cerotoylcarnitine (C26)* | -0.00885 |  | benzoylcarnitine* | 0.00014 |
| behenoylcarnitine (C22)* | -0.00879 |  | alpha-ketoglutarate | 0.00035 |
| cinnamoylglycine | -0.00878 |  | 3-hydroxyphenylacetate sulfate | 0.00055 |
| oleoyl-oleoyl-glycerol (18:1/18:1) [2]* | -0.00877 |  | butyrylcarnitine (C4) | 0.00055 |
| glycosyl ceramide (d16:1/24:1, d18:1/22:1)* | -0.00859 |  | 3-methylhistidine | 0.00075 |
| oleoyl-oleoyl-glycerol (18:1/18:1) [1]* | -0.00852 |  | N-arachidonoyltaurine | 0.00089 |
| docosatrienoate (22:3n3) | -0.00797 |  | 5-oxoproline | 0.00091 |
| sphingomyelin (d18:1/22:2, d18:2/22:1, d16:1/24:2)* | -0.00793 |  | 3-hydroxypalmitoylcarnitine | 0.00105 |
| palmitoleate (16:1n7) | -0.00792 |  | 5-dodecenoylcarnitine (C12:1) | 0.00107 |
| glycosyl ceramide (d18:1/20:0, d16:1/22:0)* | -0.00791 |  | palmitoyl-oleoyl-glycerol (16:0/18:1) [1]* | 0.00108 |
| xylose | -0.00788 |  | bilirubin (E,Z or Z,E)* | 0.00118 |
| tetradecanedioate (C14) | -0.00787 |  | choline | 0.00119 |
| sphingomyelin (d17:2/16:0, d18:2/15:0)* | -0.00754 |  | decanoylcarnitine (C10) | 0.00122 |
| 1-palmitoyl-2-docosahexaenoyl-GPE (16:0/22:6)* | -0.00729 |  | beta-hydroxyisovalerate | 0.00195 |
| docosapentaenoate (DPA; 22:5n3) | -0.00728 |  | palmitoyl-linoleoyl-glycerol (16:0/18:2) [2]* | 0.00216 |
| 3-ureidoisobutyrate | -0.00727 |  | mannose | 0.00228 |
| sphingomyelin (d18:1/20:0, d16:1/22:0)* | -0.0071 |  | gamma-glutamylhistidine | 0.00237 |
| 2-stearoyl-GPE (18:0)* | -0.00709 |  | pseudouridine | 0.00242 |
| cystine | -0.00686 |  | N-acetylneuraminate | 0.00249 |
| felinylglycine* | -0.00681 |  | 1-arachidonoyl-GPC* (20:4)* | 0.00258 |
| linoleoyl-linoleoyl-glycerol (18:2/18:2) [1]* | -0.00679 |  | linoleoyl-arachidonoyl-glycerol (18:2/20:4) [2]* | 0.00276 |
| glycosyl-N-stearoyl-sphinganine (d18:0/18:0)* | -0.00678 |  | erythronate* | 0.00306 |
| oleoyl ethanolamide | -0.00675 |  | 5-hydroxyindole sulfate | 0.00342 |
| linoleate (18:2n6) | -0.00663 |  | betaine | 0.00352 |
| hexadecatrienoate (16:3n3) | -0.00643 |  | N6-carbamoylthreonyladenosine | 0.00352 |
| methylnaphthyl sulfate (2)* | -0.00639 |  | N-acetylthreonine | 0.00356 |
| succinate | -0.00634 |  | myo-inositol | 0.00357 |
| cytidine | -0.00619 |  | N-acetylisoleucine | 0.00373 |
| margarate (17:0) | -0.00526 |  | 1-linoleoylglycerol (18:2) | 0.00379 |
| myristate (14:0) | -0.00509 |  | glycerate | 0.00394 |
| desmosterol | -0.00494 |  | sphingosine 1-phosphate | 0.00422 |
| 1,2-dilinoleoyl-GPE (18:2/18:2)* | -0.00465 |  | trimethylamine N-oxide | 0.00422 |
| stearate (18:0) | -0.00457 |  | N-acetyltaurine | 0.0045 |
| N-myristoyltaurine* | -0.00443 |  | cysteine s-sulfate | 0.00452 |
| N-stearoyl-sphingosine (d18:1/18:0)* | -0.00441 |  | 5,6-dihydrouridine | 0.00457 |
| malate | -0.0044 |  | fructose | 0.00476 |
| dodecadienoate (12:2)* | -0.0043 |  | 6-hydroxyindole sulfate | 0.00479 |
| N-acetylhistamine | -0.00405 |  | sebacate (C10-DC) | 0.00484 |
| 3-aminoisobutyrate | -0.00397 |  | 3-hydroxy-3-methylglutarate | 0.00522 |
| 1-methylnicotinamide | -0.00394 |  | indoleacetate | 0.00529 |
| 2-hydroxyoctanoate | -0.00388 |  | N-acetylfelinine* | 0.00532 |
| proline | -0.00379 |  | hexanoylcarnitine (C6) | 0.00534 |
| 1-stearoyl-2-arachidonoyl-GPE (18:0/20:4) | -0.00375 |  | 7-hydroxyindole sulfate | 0.00537 |
| glycosyl-N-(2-hydroxynervonoyl)-sphingosine (d18:1/24:1(2OH))* | -0.00375 |  | laurylcarnitine (C12) | 0.00554 |
| 3-phosphoglycerate | -0.00372 |  | ergothioneine | 0.0056 |
| sphingomyelin (d18:1/17:0, d17:1/18:0, d19:1/16:0) | -0.00371 |  | 1-oleoylglycerol (18:1) | 0.00567 |
| 5-hydroxylysine | -0.00357 |  | 3-(4-hydroxyphenyl)propionate | 0.00568 |
| threonine | -0.00321 |  | phosphocholine | 0.00595 |
| sphingomyelin (d18:1/24:1, d18:2/24:0)* | -0.00307 |  | 2'-deoxycytidine | 0.00597 |
| guaiacol sulfate | -0.00298 |  | tiglyl carnitine (C5) | 0.00602 |
| lactate | -0.00296 |  | N-acetyl-isoputreanine* | 0.00619 |
| 10-nonadecenoate (19:1n9) | -0.00284 |  | citrate | 0.00624 |
| stearidonate (18:4n3) | -0.00281 |  | cysteinylglycine | 0.00634 |
| homoarginine | -0.0028 |  | saccharin | 0.00658 |
| sphingosine | -0.0028 |  | 1-stearoyl-GPI (18:0) | 0.00665 |
| 3-(4-hydroxyphenyl)propionate | -0.00278 |  | glutathione, oxidized (GSSG) | 0.0067 |
| sphingomyelin (d18:2/16:0, d18:1/16:1)* | -0.00263 |  | thioproline | 0.00692 |
| iminodiacetate (IDA) | -0.00253 |  | 3-hydroxyoleoylcarnitine | 0.00696 |
| N-acetyl-cadaverine | -0.00245 |  | 1-methyl-4-imidazoleacetate | 0.00735 |
| 1-(1-enyl-palmitoyl)-2-arachidonoyl-GPE (P-16:0/20:4)* | -0.00225 |  | N-delta-acetylornithine | 0.00765 |
| dihomolinoleate (20:2n6) | -0.00224 |  | guanidinosuccinate | 0.00803 |
| glutamine | -0.00219 |  | 1-methylguanidine | 0.00829 |
| ribitol | -0.00202 |  | N-acetylarginine | 0.00842 |
| heneicosapentaenoate (21:5n3) | -0.00197 |  | phenylacetylalanine | 0.0086 |
| hexadecadienoate (16:2n6) | -0.00185 |  | 1-methylnicotinamide | 0.00861 |
| 3-methoxycatechol sulfate (1) | -0.00173 |  | mannitol/sorbitol | 0.00878 |
| nervonoylcarnitine (C24:1)* | -0.0015 |  | 3-hydroxy-2-ethylpropionate | 0.00882 |
| 1-oleoyl-2-linoleoyl-GPE (18:1/18:2)* | -0.00143 |  | oleoylcarnitine (C18) | 0.00888 |
| 1-oleoyl-2-arachidonoyl-GPI (18:1/20:4) * | -0.00118 |  | octadecanedioate (C18) | 0.00895 |
| glycosyl-N-stearoyl-sphingosine (d18:1/18:0) | -0.00107 |  | oleoyl-arachidonoyl-glycerol (18:1/20:4) [2]* | 0.00896 |
| caprate (10:0) | -0.00102 |  | 2-isopropylmalate | 0.0092 |
| N-arachidonoyltaurine | -0.00095 |  | gamma-glutamyltyrosine | 0.00924 |
| 1-oleoyl-2-linoleoyl-GPI (18:1/18:2)* | -0.00073 |  | N-stearoyltaurine | 0.00931 |
| saccharin | -0.00062 |  | nicotinamide riboside | 0.00953 |
| indoleacetylcarnitine* | -0.00014 |  | methyl-4-hydroxybenzoate sulfate | 0.00979 |
| 2'-O-methyluridine | -0.00007 |  | 1-methyladenosine | 0.01034 |
| benzoylcarnitine* | 0.00005 |  | gamma-glutamylphenylalanine | 0.01059 |
| gamma-glutamylfelinylglycine* | 0.00025 |  | erythritol | 0.01062 |
| alpha-hydroxyisocaproate | 0.00029 |  | nicotinamide | 0.01067 |
| 2-aminobutyrate | 0.00046 |  | nicotinate ribonucleoside | 0.01071 |
| sphingomyelin (d18:1/19:0, d19:1/18:0)* | 0.00049 |  | urea | 0.01099 |
| glycosyl-N-palmitoyl-sphingosine (d18:1/16:0) | 0.00067 |  | taurine | 0.0112 |
| 7-HOCA | 0.00077 |  | hydroxyasparagine | 0.01122 |
| N-acetyl-aspartyl-glutamate (NAAG) | 0.00084 |  | glycerophosphoglycerol | 0.01133 |
| docosapentaenoylcarnitine (C22:5n3)* | 0.00121 |  | suberoylcarnitine (C8-DC) | 0.01135 |
| alanine | 0.00127 |  | kynurenate | 0.01161 |
| 1-palmitoyl-2-arachidonoyl-GPI (16:0/20:4)* | 0.00145 |  | C-glycosyltryptophan | 0.01161 |
| eicosenoate (20:1n9 or 1n11) | 0.00165 |  | 3-hydroxyindolin-2-one sulfate | 0.01166 |
| sphingomyelin (d18:1/18:1, d18:2/18:0) | 0.00169 |  | cinnamoylglycine | 0.01166 |
| 3-hydroxymyristate | 0.00169 |  | 1-palmitoyl-GPE (16:0) | 0.01166 |
| deoxycarnitine | 0.00171 |  | 2-hydroxyoctanoate | 0.01186 |
| sphingomyelin (d18:1/20:2, d18:2/20:1, d16:1/22:2)* | 0.00179 |  | adipoylcarnitine (C6-DC) | 0.01227 |
| pentadecanoate (15:0) | 0.0018 |  | bilirubin | 0.01247 |
| 1-arachidonoyl-GPE* (20:4)* | 0.00192 |  | phosphoethanolamine (PE) | 0.01253 |
| indolelactate | 0.00239 |  | guaiacol sulfate | 0.01262 |
| 10-heptadecenoate (17:1n7) | 0.00313 |  | N-methylpipecolate | 0.01265 |
| 1-palmitoyl-2-oleoyl-GPE (16:0/18:1) | 0.00326 |  | N-acetylglutamate | 0.01273 |
| glycosyl ceramide (d18:1/23:1, d17:1/24:1)* | 0.00327 |  | 3-hydroxybutyrylcarnitine (2) | 0.0128 |
| hydroquinone sulfate | 0.00347 |  | 3-methoxytyrosine | 0.01302 |
| sarcosine | 0.0037 |  | sphinganine-1-phosphate | 0.01349 |
| oleate/vaccenate (18:1) | 0.00401 |  | adrenoylcarnitine (C22:4)* | 0.01373 |
| N-acetylleucine | 0.00418 |  | glucose | 0.01377 |
| threonate | 0.00424 |  | cortisone | 0.01381 |
| valine | 0.00446 |  | pantothenate (Vitamin B5) | 0.01384 |
| lactosyl-N-palmitoyl-sphingosine (d18:1/16:0) | 0.00448 |  | tartronate (hydroxymalonate) | 0.01414 |
| sphingomyelin (d18:2/24:1, d18:1/24:2)* | 0.00465 |  | indoleacetylglycine | 0.01444 |
| prolylhydroxyproline | 0.00469 |  | N6,N6,N6-trimethyllysine | 0.01482 |
| mannitol/sorbitol | 0.00472 |  | 3-aminoisobutyrate | 0.01483 |
| 4-hydroxyphenylacetate | 0.00475 |  | 1-ribosyl-imidazoleacetate* | 0.01483 |
| 1-linoleoyl-GPA (18:2)* | 0.00487 |  | cortisol | 0.01484 |
| phenyllactate (PLA) | 0.00509 |  | caprylate (8:0) | 0.01498 |
| alpha-ketoglutarate | 0.0051 |  | gamma-glutamylcitrulline* | 0.01504 |
| N-palmitoylglycine | 0.00512 |  | 2-aminoheptanoate | 0.01505 |
| 3-ureidopropionate | 0.00515 |  | N-acetylalanine | 0.01536 |
| arginine | 0.0052 |  | N-methylalanine | 0.01551 |
| myristoleate (14:1n5) | 0.00552 |  | ribitol | 0.01555 |
| dihydroorotate | 0.00572 |  | 3-(3-hydroxyphenyl)propionate | 0.01578 |
| palmitate (16:0) | 0.00577 |  | 3-indoxyl sulfate | 0.01592 |
| 1-linoleoyl-GPE (18:2)* | 0.00591 |  | N-acetylaspartate (NAA) | 0.01619 |
| 13-HODE + 9-HODE | 0.00593 |  | cysteinylglycine disulfide* | 0.01642 |
| glycosyl ceramide (d18:2/24:1, d18:1/24:2)* | 0.00598 |  | acetylcarnitine (C2) | 0.01643 |
| 1-(1-enyl-palmitoyl)-2-linoleoyl-GPC (P-16:0/18:2)* | 0.0061 |  | N-formylmethionine | 0.01673 |
| pyruvate | 0.00682 |  | carboxyethyl-GABA | 0.01686 |
| formiminoglutamate | 0.00684 |  | N-acetyltyrosine | 0.01702 |
| 4-hydroxyphenylpyruvate | 0.00695 |  | gamma-glutamyltryptophan | 0.0171 |
| N-oleoylserine | 0.00735 |  | allantoin | 0.01721 |
| retinal | 0.00747 |  | myristoleoylcarnitine (C14:1)* | 0.01725 |
| 1-stearoyl-2-docosahexaenoyl-GPC (18:0/22:6) | 0.00763 |  | 1-oleoyl-2-arachidonoyl-GPI (18:1/20:4) * | 0.01741 |
| 5-aminovalerate | 0.00776 |  | N6-succinyladenosine | 0.01748 |
| 1-palmitoyl-2-oleoyl-GPC (16:0/18:1) | 0.00781 |  | 16-hydroxypalmitate | 0.01749 |
| eugenol sulfate | 0.00816 |  | pyridoxamine | 0.0175 |
| octadecenedioylcarnitine (C18:1-DC)* | 0.00867 |  | N-acetylleucine | 0.01763 |
| glycerate | 0.00873 |  | trigonelline (N'-methylnicotinate) | 0.01769 |
| N6-methyladenosine | 0.00882 |  | argininosuccinate | 0.0179 |
| benzoate | 0.00887 |  | kynurenine | 0.01798 |
| pyridoxamine | 0.00891 |  | N1-methylinosine | 0.01819 |
| glycosyl-N-behenoyl-sphingadienine (d18:2/22:0)* | 0.00907 |  | N-trimethyl 5-aminovalerate | 0.01855 |
| N-acetylaspartate (NAA) | 0.00953 |  | stearoylcarnitine (C18) | 0.01867 |
| cis-4-decenoylcarnitine (C10:1) | 0.00979 |  | dimethyl sulfone | 0.01888 |
| 1-palmitoyl-GPE (16:0) | 0.0111 |  | palmitoyl-oleoyl-glycerol (16:0/18:1) [2]* | 0.01915 |
| 1-palmitoleoyl-2-linoleoyl-GPC (16:1/18:2)* | 0.01122 |  | indoleacetylglutamine | 0.01915 |
| N-palmitoyltaurine | 0.01126 |  | behenoylcarnitine (C22)* | 0.01959 |
| S-methylmethionine | 0.01127 |  | 1-arachidonoyl-GPI* (20:4)* | 0.01961 |
| indolepropionate | 0.01137 |  | gamma-glutamylmethionine | 0.01967 |
| cysteine s-sulfate | 0.0114 |  | malonylcarnitine | 0.01994 |
| alpha-ketobutyrate | 0.01163 |  | 1-stearoyl-2-linoleoyl-GPE (18:0/18:2)* | 0.01996 |
| (N(1) + N(8))-acetylspermidine | 0.01169 |  | phenylacetylglutamate | 0.01999 |
| sphingomyelin (d18:1/20:1, d18:2/20:0)* | 0.01173 |  | 3-methylglutaconate | 0.02021 |
| 4-hydroxyhippurate | 0.01179 |  | 2-methylmalonylcarnitine (C4-DC) | 0.02021 |
| phenylpyruvate | 0.01181 |  | pterin | 0.02036 |
| pterin | 0.01202 |  | diacylglycerol (14:0/18:1, 16:0/16:1) [1]* | 0.0208 |
| 1-palmitoyl-2-docosahexaenoyl-GPC (16:0/22:6) | 0.01205 |  | sarcosine | 0.0209 |
| 3-(4-hydroxyphenyl)lactate (HPLA) | 0.01208 |  | ribonate (ribonolactone) | 0.0211 |
| cysteine | 0.01223 |  | phenylalanine | 0.02129 |
| stearoyl sphingomyelin (d18:1/18:0) | 0.01247 |  | isobutyrylcarnitine (C4) | 0.02133 |
| 2'-O-methylcytidine | 0.01261 |  | p-cresol sulfate | 0.02159 |
| linoleoylcarnitine (C18:2)* | 0.01272 |  | phenylacetylglutamine | 0.02165 |
| 2-hydroxy-3-methylvalerate | 0.01392 |  | pyridoxate | 0.02174 |
| gamma-glutamyltyrosine | 0.01401 |  | isobutyrylglycine (C4) | 0.02203 |
| 1-stearoyl-GPG (18:0) | 0.01427 |  | 1-palmitoyl-2-arachidonoyl-GPI (16:0/20:4)* | 0.02207 |
| 2-hydroxyphenylacetate | 0.01443 |  | 2-hydroxydecanoate | 0.02211 |
| glucuronate | 0.01447 |  | orotidine | 0.02218 |
| N-acetylglucosaminylasparagine | 0.01451 |  | 1,2-dilinoleoyl-GPE (18:2/18:2)* | 0.02221 |
| 4-cholesten-3-one | 0.01486 |  | methionine sulfone | 0.02226 |
| maleate | 0.01525 |  | stachydrine | 0.02232 |
| 5alpha-pregnan-3beta,20beta-diol monosulfate (1) | 0.01566 |  | 4-vinylphenol sulfate | 0.02235 |
| gamma-glutamylmethionine | 0.0157 |  | indolepropionate | 0.02245 |
| 4-hydroxyphenylacetylglycine | 0.01577 |  | 1-linoleoyl-2-arachidonoyl-GPE (18:2/20:4)* | 0.02261 |
| S-methylcysteine sulfoxide | 0.01579 |  | N-acetyl-beta-alanine | 0.02282 |
| 3-methyl-2-oxobutyrate | 0.01606 |  | 1-myristoyl-2-palmitoyl-GPC (14:0/16:0) | 0.02295 |
| felinine | 0.01645 |  | anthranilate | 0.0234 |
| methionine sulfoxide | 0.01664 |  | 2,4-dichlorophenol sulfate | 0.02364 |
| tartronate (hydroxymalonate) | 0.01665 |  | 5-bromotryptophan | 0.02365 |
| imidazole lactate | 0.01667 |  | 5-(galactosylhydroxy)-L-lysine | 0.02409 |
| ornithine | 0.01692 |  | N-acetylmethionine | 0.02421 |
| 2-methylserine | 0.01713 |  | phenylacetate | 0.0243 |
| arachidoylcarnitine (C20)* | 0.01745 |  | azeloylcarnitine (C9-DC) | 0.02473 |
| phosphocholine | 0.01797 |  | succinylcarnitine (C4) | 0.02504 |
| dihomo-linolenoylcarnitine (C20:3n3 or 6)* | 0.01835 |  | 1-stearoyl-2-arachidonoyl-GPI (18:0/20:4) | 0.02508 |
| pyridoxal | 0.01847 |  | N-acetylglucosaminylasparagine | 0.02522 |
| N-acetylcarnosine | 0.01857 |  | sedoheptulose | 0.02528 |
| pyroglutamine* | 0.01903 |  | 1-methylhypoxanthine | 0.02529 |
| dihomo-linoleoylcarnitine (C20:2)* | 0.01924 |  | gluconate | 0.0253 |
| picolinate | 0.01953 |  | benzoate | 0.02537 |
| 1-palmitoyl-2-arachidonoyl-GPE (16:0/20:4)* | 0.01994 |  | stearoylcholine* | 0.0254 |
| uracil | 0.02042 |  | 1-(1-enyl-palmitoyl)-2-oleoyl-GPC (P-16:0/18:1)* | 0.02551 |
| prolylglycine | 0.02066 |  | 1-stearoyl-GPE (18:0) | 0.02568 |
| 2-oxoadipate | 0.02079 |  | 9,10-DiHOME | 0.02573 |
| 3-methoxycatechol sulfate (2) | 0.02101 |  | pimeloylcarnitine/3-methyladipoylcarnitine (C7-DC) | 0.02587 |
| propionylglycine (C3) | 0.02133 |  | 1-stearoyl-GPC (18:0) | 0.02589 |
| N-linoleoyltaurine* | 0.02146 |  | 4-methylcatechol sulfate | 0.02606 |
| catechol sulfate | 0.02147 |  | isovalerylcarnitine (C5) | 0.02612 |
| methyl indole-3-acetate | 0.02168 |  | propionylcarnitine (C3) | 0.02615 |
| margaroylcarnitine (C17)* | 0.02176 |  | aconitate [cis or trans] | 0.02617 |
| malonate | 0.02179 |  | oxalate (ethanedioate) | 0.02618 |
| histidine | 0.02338 |  | lanthionine | 0.02672 |
| 3-hydroxylaurate | 0.02363 |  | dopamine 3-O-sulfate | 0.02674 |
| cysteine-glutathione disulfide | 0.02371 |  | N-stearoyl-sphinganine (d18:0/18:0)* | 0.02681 |
| 1-(1-enyl-palmitoyl)-2-oleoyl-GPC (P-16:0/18:1)* | 0.02375 |  | N-acetylglutamine | 0.02686 |
| erucoylcarnitine (C22:1)* | 0.02426 |  | 3-acetylphenol sulfate | 0.0269 |
| 1-linoleoyl-GPG (18:2)* | 0.02482 |  | 1-stearoyl-2-linoleoyl-GPC (18:0/18:2)* | 0.02693 |
| 3beta-hydroxy-5-cholestenoate | 0.02495 |  | N-acetylputrescine | 0.02694 |
| 1-(1-enyl-palmitoyl)-2-palmitoyl-GPC (P-16:0/16:0)* | 0.02565 |  | methionine | 0.02707 |
| 2-oxoarginine* | 0.02567 |  | retinal | 0.02718 |
| N-acetylglycine | 0.02593 |  | 5-hydroxyindoleacetate | 0.02732 |
| N-delta-acetylornithine | 0.02605 |  | phenylacetylcarnitine | 0.02751 |
| 1-palmitoyl-2-linoleoyl-GPE (16:0/18:2) | 0.02606 |  | glycerol 3-phosphate | 0.02783 |
| gamma-glutamylglutamate | 0.02617 |  | linoleoylcholine* | 0.02821 |
| 16-hydroxypalmitate | 0.02675 |  | 4-acetamidobutanoate | 0.02845 |
| 2-piperidinone | 0.02694 |  | biliverdin | 0.02865 |
| 1,5-anhydroglucitol (1,5-AG) | 0.02787 |  | threonate | 0.02886 |
| 5-methylcytidine | 0.02815 |  | 7-HOCA | 0.02887 |
| N-acetylkynurenine (2) | 0.02918 |  | N-methylproline | 0.02889 |
| indoleacetylglutamine | 0.02932 |  | ferulic acid 4-sulfate | 0.0291 |
| N2,N2-dimethylguanine | 0.02972 |  | 1-oleoyl-GPI (18:1)* | 0.02921 |
| 3-hydroxyhippurate | 0.03011 |  | N2,N2-dimethylguanine | 0.0294 |
| dodecanedioate (C12) | 0.03013 |  | 2-oxindole-3-acetate | 0.02943 |
| octadecanedioylcarnitine (C18-DC)* | 0.0305 |  | 1-myristoyl-2-arachidonoyl-GPC (14:0/20:4)* | 0.02959 |
| azelate (nonanedioate; C9) | 0.03068 |  | 4-ethylphenyl sulfate | 0.02965 |
| N-acetylphenylalanine | 0.03091 |  | 1-palmitoyl-GPI* (16:0) | 0.02971 |
| 4-guanidinobutanoate | 0.03128 |  | citrulline | 0.02984 |
| oxalate (ethanedioate) | 0.03129 |  | octadecenedioate (C18:1-DC)* | 0.0299 |
| 2-aminophenol sulfate | 0.03184 |  | dodecanedioate (C12) | 0.03021 |
| riboflavin (Vitamin B2) | 0.03203 |  | methionine sulfoxide | 0.03024 |
| pyrraline | 0.03239 |  | 1,2,3-benzenetriol sulfate (2) | 0.03028 |
| oleoylcarnitine (C18) | 0.0325 |  | ceramide (d18:1/14:0, d16:1/16:0)* | 0.03041 |
| glycine | 0.0327 |  | 3-phenylpropionate (hydrocinnamate) | 0.03043 |
| palmitoleoylcarnitine (C16:1)* | 0.03279 |  | 4-hydroxyhippurate | 0.03058 |
| N-trimethyl 5-aminovalerate | 0.03306 |  | riboflavin (Vitamin B2) | 0.03061 |
| 5-dodecenoylcarnitine (C12:1) | 0.03334 |  | 1-palmitoyl-2-alpha-linolenoyl-GPC (16:0/18:3n3)* | 0.03074 |
| ectoine | 0.03356 |  | 2,3-dihydroxyisovalerate | 0.03124 |
| 1-palmitoyl-GPG (16:0)* | 0.03375 |  | 7-methylguanine | 0.03125 |
| perfluorooctanesulfonic acid (PFOS) | 0.03384 |  | uracil | 0.03171 |
| phosphate | 0.0342 |  | stearoyl-arachidonoyl-glycerol (18:0/20:4) [2]* | 0.03192 |
| glycerol | 0.03444 |  | glycerophosphorylcholine (GPC) | 0.03206 |
| 4-hydroxyphenylacetate sulfate | 0.03511 |  | N1-Methyl-2-pyridone-5-carboxamide | 0.03237 |
| urate | 0.03526 |  | formiminoglutamate | 0.0325 |
| gamma-glutamylthreonine | 0.03571 |  | oleoylcholine | 0.03262 |
| gulonate* | 0.036 |  | N-acetylvaline | 0.03285 |
| cytosine | 0.03646 |  | suberate (C8-DC) | 0.03303 |
| lanthionine | 0.03735 |  | margaroylcarnitine (C17)* | 0.03327 |
| gamma-glutamylalanine | 0.0376 |  | N-acetylphenylalanine | 0.03363 |
| trans-urocanate | 0.03824 |  | gamma-glutamyl-epsilon-lysine | 0.03371 |
| 1,2,3-benzenetriol sulfate (2) | 0.03831 |  | stearoyl-arachidonoyl-glycerol (18:0/20:4) [1]* | 0.03428 |
| stearoylcarnitine (C18) | 0.03843 |  | cystine | 0.03429 |
| N-glycolylneuraminate | 0.03865 |  | N-stearoylserine* | 0.03462 |
| N-acetylputrescine | 0.03898 |  | N-acetylkynurenine (2) | 0.03464 |
| 1-oleoyl-2-docosahexaenoyl-GPC (18:1/22:6)* | 0.03902 |  | pyridoxal | 0.03472 |
| cortisone | 0.03926 |  | 1-oleoyl-2-linoleoyl-GPE (18:1/18:2)* | 0.03476 |
| anserine | 0.03956 |  | hexadecanedioate (C16) | 0.03481 |
| lysine | 0.03976 |  | carotene diol (2) | 0.03504 |
| alpha-hydroxyisovalerate | 0.04076 |  | arabonate/xylonate | 0.0352 |
| glycerol 3-phosphate | 0.04078 |  | 1-stearoyl-2-arachidonoyl-GPS (18:0/20:4) | 0.03522 |
| N-acetylalanine | 0.04101 |  | azelate (nonanedioate; C9) | 0.03523 |
| 5-oxoproline | 0.04147 |  | eicosenoylcarnitine (C20:1)* | 0.03533 |
| 3-hydroxydecanoate | 0.04171 |  | indoleacetylcarnitine* | 0.03539 |
| myristoylcarnitine (C14) | 0.04183 |  | N-acetylserine | 0.03543 |
| laurylcarnitine (C12) | 0.04215 |  | 2-hydroxystearate | 0.03552 |
| gamma-glutamylvaline | 0.04218 |  | 5-hydroxymethyl-2'-deoxycytidine | 0.03598 |
| 4-acetylphenyl sulfate | 0.0426 |  | sphingomyelin (d18:0/18:0, d19:0/17:0)* | 0.03614 |
| mevalonolactone | 0.04269 |  | trans-urocanate | 0.03619 |
| 2-aminoadipate | 0.04309 |  | 3beta-hydroxy-5-cholestenoate | 0.03621 |
| 1-methyl-4-imidazoleacetate | 0.04354 |  | sphingomyelin (d18:1/18:1, d18:2/18:0) | 0.03644 |
| hydroxyproline | 0.04362 |  | dihomo-linoleoylcarnitine (C20:2)* | 0.03672 |
| gamma-glutamylglutamine | 0.04383 |  | hexadecenedioate (C16:1-DC)* | 0.03686 |
| S-adenosylhomocysteine (SAH) | 0.04398 |  | stearoyl sphingomyelin (d18:1/18:0) | 0.03694 |
| S-1-pyrroline-5-carboxylate | 0.04416 |  | diacylglycerol (14:0/18:1, 16:0/16:1) [2]* | 0.03701 |
| carnitine | 0.04451 |  | 5-methylcytidine | 0.03741 |
| orotate | 0.04501 |  | docosapentaenoylcarnitine (C22:5n3)* | 0.03746 |
| carboxyethyl-GABA | 0.0453 |  | N-acetyltryptophan | 0.03753 |
| 4-hydroxycinnamate sulfate | 0.04557 |  | hydroquinone sulfate | 0.03792 |
| citrulline | 0.0458 |  | behenate (22:0)* | 0.03907 |
| phenylacetylalanine | 0.04587 |  | palmitoylcholine | 0.0391 |
| N-oleoyltaurine | 0.04593 |  | 1-linoleoyl-GPI* (18:2)* | 0.03917 |
| N-acetylglutamine | 0.04597 |  | nervonoylcarnitine (C24:1)* | 0.03924 |
| ribonate (ribonolactone) | 0.04632 |  | xanthine | 0.0395 |
| 3-hydroxybutyrate (BHBA) | 0.0464 |  | cystathionine | 0.03953 |
| homostachydrine* | 0.04649 |  | 1-linolenoyl-GPC (18:3)* | 0.04065 |
| anthranilate | 0.04664 |  | 4-hydroxycinnamate sulfate | 0.04067 |
| cysteinylglycine | 0.04667 |  | lignoceroylcarnitine (C24)* | 0.04111 |
| N-monomethylarginine | 0.04673 |  | ribulonate/xylulonate* | 0.0412 |
| octanoylcarnitine (C8) | 0.04676 |  | 1-palmitoyl-2-arachidonoyl-GPC (16:0/20:4n6) | 0.04167 |
| N1-Methyl-2-pyridone-5-carboxamide | 0.04677 |  | phenol sulfate | 0.04181 |
| carnosine | 0.04677 |  | 4-methoxyphenol sulfate | 0.04255 |
| N6-acetyllysine | 0.04722 |  | 5-methylcytosine | 0.04282 |
| 2-hydroxyglutarate | 0.04725 |  | desmosterol | 0.04331 |
| dimethylglycine | 0.04806 |  | 2-methylbutyroylcarnitine (C5) | 0.04388 |
| imidazole propionate | 0.04814 |  | perfluorooctanesulfonic acid (PFOS) | 0.04406 |
| kynurenate | 0.0485 |  | 1-stearoyl-2-oleoyl-GPE (18:0/18:1) | 0.04416 |
| 5-hydroxymethylcytidine | 0.04862 |  | heme | 0.04434 |
| myristoleoylcarnitine (C14:1)* | 0.04876 |  | pyrraline | 0.04455 |
| 5-methylcytosine | 0.04917 |  | glycerophosphoethanolamine | 0.04464 |
| cystathionine | 0.04925 |  | lactosyl-N-palmitoyl-sphingosine (d18:1/16:0) | 0.04513 |
| glycerophosphoglycerol | 0.0493 |  | 5-methylthioadenosine (MTA) | 0.04516 |
| 4-methoxyphenol sulfate | 0.04954 |  | tetradecanedioate (C14) | 0.04556 |
| sulfate* | 0.04982 |  | 3-methoxycatechol sulfate (2) | 0.04582 |
| butyrylcarnitine (C4) | 0.04985 |  | linoleoyl ethanolamide | 0.04615 |
| 4-vinylguaiacol sulfate | 0.04991 |  | 3-hydroxyhippurate | 0.04617 |
| phenylacetylserine | 0.05006 |  | 1-(1-enyl-stearoyl)-2-linoleoyl-GPE (P-18:0/18:2)* | 0.04618 |
| palmitoylcarnitine (C16) | 0.05042 |  | 1-linoleoyl-2-arachidonoyl-GPC (18:2/20:4n6)* | 0.04632 |
| N-acetyltryptophan | 0.05098 |  | 2-aminophenol sulfate | 0.04653 |
| decanoylcarnitine (C10) | 0.05098 |  | 1-(1-enyl-stearoyl)-2-oleoyl-GPE (P-18:0/18:1) | 0.04718 |
| hexanoylglycine (C6) | 0.05116 |  | sphingadienine | 0.04795 |
| indoleacetylglycine | 0.05154 |  | 1-arachidonoyl-GPE* (20:4)* | 0.04796 |
| propionylcarnitine (C3) | 0.05162 |  | urate | 0.04841 |
| glutathione, oxidized (GSSG) | 0.05171 |  | 1-palmitoyl-2-stearoyl-GPC (16:0/18:0) | 0.04855 |
| cysteinylglycine disulfide* | 0.05208 |  | 3-(3-hydroxyphenyl)propionate sulfate | 0.04895 |
| taurine | 0.0521 |  | erucoylcarnitine (C22:1)* | 0.04909 |
| N-methylpipecolate | 0.05255 |  | campesterol | 0.04937 |
| N-formylmethionine | 0.05267 |  | ximenoylcarnitine (C26:1)* | 0.05017 |
| phenol sulfate | 0.05335 |  | beta-sitosterol | 0.05051 |
| adenosine | 0.05361 |  | gulonate* | 0.05051 |
| eicosenoylcarnitine (C20:1)* | 0.05367 |  | 3-hydroxypyridine sulfate | 0.05071 |
| N-methylalanine | 0.05377 |  | 2'-deoxyuridine | 0.05103 |
| pipecolate | 0.05415 |  | heptanoate (7:0) | 0.05115 |
| caprylate (8:0) | 0.05423 |  | 1-linoleoyl-GPC (18:2) | 0.05134 |
| N-acetyl-beta-alanine | 0.05426 |  | 1-palmitoyl-2-linoleoyl-GPI (16:0/18:2) | 0.05153 |
| homocystine | 0.05449 |  | 1-palmitoyl-2-linoleoyl-GPC (16:0/18:2) | 0.05252 |
| 3-(3-hydroxyphenyl)propionate sulfate | 0.05476 |  | 5-methyl-2'-deoxycytidine | 0.05277 |
| 3-acetylphenol sulfate | 0.05483 |  | N-oleoylserine | 0.05278 |
| N-palmitoleoyltaurine* | 0.05533 |  | 2-palmitoyl-GPC* (16:0)* | 0.05278 |
| 2,3-dihydroxyisovalerate | 0.05538 |  | 1-(1-enyl-palmitoyl)-2-palmitoyl-GPC (P-16:0/16:0)* | 0.05279 |
| 3-hydroxyisobutyrate | 0.05553 |  | 3-ureidoisobutyrate | 0.05289 |
| gamma-glutamylphenylalanine | 0.05564 |  | 1-oleoyl-2-linoleoyl-GPI (18:1/18:2)* | 0.05317 |
| 2-isopropylmalate | 0.0557 |  | 1-palmitoyl-2-linoleoyl-GPE (16:0/18:2) | 0.0533 |
| arabonate/xylonate | 0.05577 |  | sphingomyelin (d18:1/25:0, d19:0/24:1, d20:1/23:0, d19:1/24:0)* | 0.0533 |
| gamma-glutamylcitrulline* | 0.05656 |  | cholate | 0.05347 |
| gamma-glutamyltryptophan | 0.0571 |  | 1-palmitoleoyl-2-linoleoyl-GPC (16:1/18:2)* | 0.05358 |
| alpha-ketoglutaramate | 0.05711 |  | 1-palmitoyl-2-palmitoleoyl-GPC (16:0/16:1)* | 0.05381 |
| N-acetyltyrosine | 0.0577 |  | S-methylmethionine | 0.05431 |
| mevalonate | 0.05776 |  | 1-palmitoleoyl-2-linolenoyl-GPC (16:1/18:3)* | 0.05441 |
| hexanoylcarnitine (C6) | 0.05816 |  | 2-hydroxypalmitate | 0.05446 |
| 3-hydroxyhexanoate | 0.05821 |  | picolinate | 0.05448 |
| argininate* | 0.05824 |  | 2'-O-methylcytidine | 0.05451 |
| N-acetylmethionine | 0.05856 |  | 1-stearoyl-2-linoleoyl-GPI (18:0/18:2) | 0.05462 |
| N-acetylisoleucine | 0.0587 |  | catechol sulfate | 0.05496 |
| 3-hydroxyoctanoate | 0.05871 |  | chenodeoxycholate | 0.05529 |
| 4-ethylphenyl sulfate | 0.05922 |  | arachidoylcarnitine (C20)* | 0.0553 |
| malonylcarnitine | 0.05928 |  | 3-ureidopropionate | 0.0553 |
| N-acetylvaline | 0.05983 |  | 1-stearoyl-2-oleoyl-GPI (18:0/18:1)* | 0.05564 |
| isovalerylglycine | 0.0601 |  | behenoyl dihydrosphingomyelin (d18:0/22:0)* | 0.05642 |
| trimethylamine N-oxide | 0.06016 |  | 1,2-dipalmitoyl-GPC (16:0/16:0) | 0.0576 |
| phenylacetylglutamine | 0.06042 |  | taurolithocholate 3-sulfate | 0.05767 |
| 1-methyladenosine | 0.0608 |  | carotene diol (3) | 0.05794 |
| 3-hydroxypyridine sulfate | 0.06087 |  | 1-(1-enyl-palmitoyl)-2-arachidonoyl-GPC (P-16:0/20:4)* | 0.05834 |
| isovalerylcarnitine (C5) | 0.06136 |  | 2'-O-methyluridine | 0.05842 |
| 2-hydroxybutyrate/2-hydroxyisobutyrate | 0.06159 |  | cytosine | 0.05856 |
| N6-succinyladenosine | 0.06163 |  | ceramide (d18:2/24:1, d18:1/24:2)* | 0.05901 |
| 3-hydroxyphenylacetate sulfate | 0.06211 |  | 1-palmitoyl-GPG (16:0)* | 0.05912 |
| phenylacetate | 0.06242 |  | taurocholate | 0.05912 |
| 4-vinylphenol sulfate | 0.06255 |  | taurochenodeoxycholate | 0.05935 |
| ophthalmate | 0.06259 |  | N-palmitoyl-sphinganine (d18:0/16:0) | 0.05946 |
| N-acetylglutamate | 0.06283 |  | ceramide (d18:1/17:0, d17:1/18:0)* | 0.05983 |
| ribulonate/xylulonate* | 0.06292 |  | N2,N2-dimethylguanosine | 0.06037 |
| 2-methylbutyroylcarnitine (C5) | 0.06329 |  | glucuronate | 0.06046 |
| 4-methylcatechol sulfate | 0.06347 |  | glycosyl-N-stearoyl-sphinganine (d18:0/18:0)* | 0.06098 |
| 1-methylhistidine | 0.06375 |  | methyl indole-3-acetate | 0.06104 |
| 5-hydroxyindole sulfate | 0.06425 |  | N-stearoyl-sphingosine (d18:1/18:0)* | 0.06121 |
| 1-ribosyl-imidazoleacetate* | 0.06438 |  | cerotoylcarnitine (C26)* | 0.06136 |
| glutaroylcarnitine (C5) | 0.06452 |  | 1-palmitoyl-2-oleoyl-GPE (16:0/18:1) | 0.06158 |
| azeloylcarnitine (C9-DC) | 0.06466 |  | 1-linoleoyl-2-linolenoyl-GPC (18:2/18:3)* | 0.06229 |
| 6-oxopiperidine-2-carboxylate | 0.06504 |  | oleoyl ethanolamide | 0.06233 |
| N-acetylneuraminate | 0.06511 |  | N-palmitoyl-sphingadienine (d18:2/16:0)* | 0.06234 |
| argininosuccinate | 0.06513 |  | 1-stearoyl-2-oleoyl-GPC (18:0/18:1) | 0.06239 |
| phenylacetylglycine | 0.06551 |  | ceramide (d16:1/24:1, d18:1/22:1)* | 0.06266 |
| myo-inositol | 0.06571 |  | octadecanedioylcarnitine (C18-DC)* | 0.06269 |
| dimethyl sulfone | 0.06593 |  | 1-oleoyl-2-linoleoyl-GPC (18:1/18:2)* | 0.0629 |
| gamma-glutamylisoleucine* | 0.06619 |  | 1-(1-enyl-palmitoyl)-2-linoleoyl-GPC (P-16:0/18:2)* | 0.06314 |
| gamma-glutamylleucine | 0.06626 |  | 1-(1-enyl-palmitoyl)-GPE (P-16:0)* | 0.06388 |
| N-methylproline | 0.0663 |  | 4-acetylphenyl sulfate | 0.06401 |
| 6-hydroxyindole sulfate | 0.0665 |  | 1-palmitoyl-2-oleoyl-GPI (16:0/18:1)* | 0.0644 |
| O-sulfo-L-tyrosine | 0.06701 |  | myristoyl dihydrosphingomyelin (d18:0/14:0)* | 0.06473 |
| 3-methylhistidine | 0.06701 |  | dihomo-linolenoylcarnitine (C20:3n3 or 6)* | 0.06474 |
| 7-hydroxyindole sulfate | 0.06706 |  | 1-palmitoleoyl-GPC* (16:1)* | 0.06524 |
| citrate | 0.06707 |  | 1-stearoyl-GPG (18:0) | 0.0657 |
| N-acetylserine | 0.06771 |  | sphingomyelin (d18:0/20:0, d16:0/22:0)* | 0.06579 |
| 3-hydroxypalmitoylcarnitine | 0.06776 |  | caproate (6:0) | 0.06612 |
| 5-methylthioadenosine (MTA) | 0.06779 |  | alpha-tocopherol | 0.06635 |
| orotidine | 0.06851 |  | sphingosine | 0.06653 |
| arabitol/xylitol | 0.0686 |  | taurodeoxycholate | 0.06695 |
| succinylcarnitine (C4) | 0.06861 |  | sphingomyelin (d18:1/20:1, d18:2/20:0)* | 0.06696 |
| gamma-glutamyl-epsilon-lysine | 0.06907 |  | 1-palmitoyl-GPC (16:0) | 0.06698 |
| butyrylglycine (C4) | 0.06951 |  | palmitoyl dihydrosphingomyelin (d18:0/16:0)* | 0.06749 |
| dimethylarginine (ADMA + SDMA) | 0.06957 |  | alpha-CEHC sulfate | 0.06752 |
| nicotinamide riboside | 0.06958 |  | 1-(1-enyl-stearoyl)-2-arachidonoyl-GPE (P-18:0/20:4)* | 0.06766 |
| 3-hydroxyindolin-2-one sulfate | 0.06974 |  | 1-(1-enyl-oleoyl)-GPE (P-18:1)* | 0.06771 |
| 3-hydroxyoleoylcarnitine | 0.06986 |  | glycosyl ceramide (d18:1/23:1, d17:1/24:1)* | 0.06852 |
| homocitrulline | 0.07022 |  | valerate (5:0) | 0.06883 |
| gamma-glutamylglycine | 0.07024 |  | 1-(1-enyl-stearoyl)-GPE (P-18:0)* | 0.06932 |
| guanidinosuccinate | 0.07045 |  | N-behenoyl-sphingadienine (d18:2/22:0)* | 0.06958 |
| suberate (C8-DC) | 0.07054 |  | octadecenedioylcarnitine (C18:1-DC)* | 0.06997 |
| pseudouridine | 0.0707 |  | sphingomyelin (d18:2/24:1, d18:1/24:2)* | 0.07009 |
| pimeloylcarnitine/3-methyladipoylcarnitine (C7-DC) | 0.07085 |  | 1-linoleoyl-GPG (18:2)* | 0.07015 |
| 3-indoxyl sulfate | 0.0712 |  | 4-cholesten-3-one | 0.07031 |
| gamma-glutamylhistidine | 0.07121 |  | taurocholenate sulfate | 0.07077 |
| retinol (Vitamin A) | 0.07124 |  | 1-(1-enyl-palmitoyl)-GPC (P-16:0)* | 0.07148 |
| hydroxyasparagine | 0.07137 |  | sphingomyelin (d18:1/17:0, d17:1/18:0, d19:1/16:0) | 0.07178 |
| tiglyl carnitine (C5) | 0.07137 |  | sphingomyelin (d18:1/20:0, d16:1/22:0)* | 0.07186 |
| 5-(galactosylhydroxy)-L-lysine | 0.07159 |  | 1-(1-enyl-palmitoyl)-2-oleoyl-GPE (P-16:0/18:1)* | 0.07194 |
| adipoylcarnitine (C6-DC) | 0.07164 |  | 1-oleoyl-GPE (18:1) | 0.07216 |
| creatinine | 0.07174 |  | sphingomyelin (d18:2/24:2)* | 0.07249 |
| N-acetylthreonine | 0.07186 |  | glycosyl-N-stearoyl-sphingosine (d18:1/18:0) | 0.07276 |
| N-acetylfelinine* | 0.07195 |  | 1-oleoyl-GPC (18:1) | 0.07301 |
| 2-oxindole-3-acetate | 0.07289 |  | sphinganine | 0.07387 |
| isobutyrylcarnitine (C4) | 0.0732 |  | sphingomyelin (d18:1/14:0, d16:1/16:0)* | 0.07396 |
| N6,N6,N6-trimethyllysine | 0.07331 |  | isovalerate (C5) | 0.074 |
| p-cresol sulfate | 0.07367 |  | 1-linoleoyl-GPE (18:2)* | 0.07548 |
| N6-carbamoylthreonyladenosine | 0.07368 |  | sphingomyelin (d18:1/24:1, d18:2/24:0)* | 0.07621 |
| 4-acetamidobutanoate | 0.07395 |  | deoxycholate | 0.07625 |
| N-acetylarginine | 0.07424 |  | carotene diol (1) | 0.07638 |
| acetylcarnitine (C2) | 0.07424 |  | glycosyl-N-palmitoyl-sphingosine (d18:1/16:0) | 0.07703 |
| erythritol | 0.07436 |  | sphingomyelin (d18:2/18:1)* | 0.07774 |
| phenylacetylcarnitine | 0.07445 |  | glycosyl ceramide (d16:1/24:1, d18:1/22:1)* | 0.07856 |
| C-glycosyltryptophan | 0.0745 |  | ceramide (d18:1/20:0, d16:1/22:0, d20:1/18:0)* | 0.07902 |
| methionine sulfone | 0.07465 |  | sphingomyelin (d18:2/16:0, d18:1/16:1)* | 0.07924 |
| 1-methylhypoxanthine | 0.07503 |  | N-palmitoyl-sphingosine (d18:1/16:0) | 0.07929 |
| pyridoxate | 0.07521 |  | sphingomyelin (d18:1/22:1, d18:2/22:0, d16:1/24:1)* | 0.07995 |
| 5-hydroxyindoleacetate | 0.07577 |  | 1,2-dilinoleoyl-GPC (18:2/18:2) | 0.08005 |
| N1-methylinosine | 0.0758 |  | sphingomyelin (d18:1/20:2, d18:2/20:1, d16:1/22:2)* | 0.08049 |
| 3-methylglutaconate | 0.0765 |  | lignoceroyl sphingomyelin (d18:1/24:0) | 0.08083 |
| N4-acetylcytidine | 0.07653 |  | 1-(1-enyl-palmitoyl)-2-arachidonoyl-GPE (P-16:0/20:4)* | 0.081 |
| isobutyrylglycine (C4) | 0.0767 |  | tricosanoyl sphingomyelin (d18:1/23:0)* | 0.08169 |
| allantoin | 0.07675 |  | sphingomyelin (d18:1/22:2, d18:2/22:1, d16:1/24:2)* | 0.0823 |
| N-acetyl-isoputreanine* | 0.07703 |  | sphingomyelin (d17:2/16:0, d18:2/15:0)* | 0.08368 |
| phenylacetylglutamate | 0.0771 |  | sphingomyelin (d18:2/14:0, d18:1/14:1)* | 0.08555 |
| trigonelline (N'-methylnicotinate) | 0.07717 |  | sphingomyelin (d18:2/21:0, d16:2/23:0)* | 0.08556 |
| stachydrine | 0.07733 |  | 1-lignoceroyl-GPC (24:0) | 0.0862 |
| aconitate [cis or trans] | 0.07775 |  | glycosyl ceramide (d18:1/20:0, d16:1/22:0)* | 0.08625 |
| 7-methylguanine | 0.07789 |  | sphingomyelin (d18:2/23:1)* | 0.08667 |
| beta-hydroxyisovalerate | 0.07793 |  | sphingomyelin (d17:1/14:0, d16:1/15:0)* | 0.08782 |
| suberoylcarnitine (C8-DC) | 0.07793 |  | glycosyl-N-(2-hydroxynervonoyl)-sphingosine (d18:1/24:1(2OH))* | 0.08817 |
| 3-hydroxy-3-methylglutarate | 0.07808 |  | palmitoyl sphingomyelin (d18:1/16:0) | 0.0882 |
| allantoic acid | 0.07817 |  | sphingomyelin (d18:2/23:0, d18:1/23:1, d17:1/24:1)* | 0.0886 |
| 1-methylguanidine | 0.07843 |  | 1-palmitoyl-2-oleoyl-GPC (16:0/18:1) | 0.08879 |
| 2-methylmalonylcarnitine (C4-DC) | 0.07885 |  | sphingomyelin (d18:1/21:0, d17:1/22:0, d16:1/23:0)* | 0.09019 |
| 3-hydroxybutyrylcarnitine (2) | 0.07936 |  | sphingomyelin (d17:1/16:0, d18:1/15:0, d16:1/17:0)* | 0.09022 |
| 5,6-dihydrouridine | 0.07967 |  | cholesterol | 0.09111 |
| erythronate* | 0.0797 |  | glycosyl ceramide (d18:2/24:1, d18:1/24:2)* | 0.09184 |
| N-acetyltaurine | 0.08046 |  | glycosyl-N-behenoyl-sphingadienine (d18:2/22:0)* | 0.09258 |
| urea | 0.08113 |  | behenoyl sphingomyelin (d18:1/22:0)* | 0.09408 |
| 3-hydroxy-2-ethylpropionate | 0.08126 |  | sphingomyelin (d18:1/19:0, d19:1/18:0)* | 0.09628 |
|  |  |  |  |  |
