# Supplementary material for: Chronic kidney disease in cats alters response of the plasma metabolome and fecal microbiome to dietary fiber
Source: PLoS One. 2020 Jul 2;15(7):e0235480. doi: 10.1371/journal.pone.0235480 (PMC7331996; doi:10.1371/journal.pone.0235480)
Supplement: S3 Table — (DOCX) [file pone.0235480.s003.docx]

**Table S3.** Eigenvector metabolites that contributed to separation of the two groups in Figure 1B, PC1 and PC2.

| **Figure 1B** | **PC1** | **Figure 1B** | **PC2** |
| --- | --- | --- | --- |
| 1-arachidonoyl-GPC* (20:4)* | -0.06363 | leucine | -0.0723 |
| glycerophosphorylcholine (GPC) | -0.06296 | 1-stearoyl-2-arachidonoyl-GPE (18:0/20:4) | -0.07078 |
| tryptophan | -0.06213 | 1-palmitoyl-2-arachidonoyl-GPE (16:0/20:4)* | -0.06814 |
| 1-stearoyl-GPC (18:0) | -0.06105 | 1,2-dilinoleoyl-GPE (18:2/18:2)* | -0.06757 |
| mannose | -0.05969 | 1-stearoyl-2-oleoyl-GPS (18:0/18:1) | -0.06311 |
| 5-bromotryptophan | -0.05728 | 1-stearoyl-2-linoleoyl-GPE (18:0/18:2)* | -0.06291 |
| 1-linoleoyl-2-arachidonoyl-GPC (18:2/20:4n6)* | -0.056 | 1-stearoyl-2-arachidonoyl-GPS (18:0/20:4) | -0.06216 |
| 1-arachidonoyl-GPI* (20:4)* | -0.05197 | 3-phosphoglycerate | -0.06136 |
| 3-methoxytyrosine | -0.04995 | taurine | -0.06071 |
| serine | -0.04888 | 1-stearoyl-2-linoleoyl-GPS (18:0/18:2) | -0.06064 |
| adenine | -0.04884 | phosphoethanolamine (PE) | -0.06062 |
| 1-linoleoylglycerol (18:2) | -0.04881 | 1-(1-enyl-stearoyl)-2-arachidonoyl-GPE (P-18:0/20:4)* | -0.05895 |
| 1-linoleoyl-GPC (18:2) | -0.04872 | cysteine-glutathione disulfide | -0.05875 |
| bilirubin | -0.04841 | aspartate | -0.05851 |
| 1-oleoyl-GPI (18:1)* | -0.04805 | inosine 5'-monophosphate (IMP) | -0.05833 |
| creatine | -0.04792 | N-glycolylneuraminate | -0.05794 |
| docosadioate (C22-DC) | -0.04782 | thioproline | -0.05771 |
| 1-stearoyl-GPI (18:0) | -0.04762 | succinate | -0.0566 |
| dihomolinolenate (20:3n3 or 3n6) | -0.04683 | 1-palmitoyl-2-docosahexaenoyl-GPE (16:0/22:6)* | -0.05595 |
| S-methylcysteine | -0.04668 | 1-linoleoyl-2-arachidonoyl-GPE (18:2/20:4)* | -0.05593 |
| arachidonoylcholine | -0.04638 | isoleucine | -0.05419 |
| 2-hydroxynervonate* | -0.04628 | 1-stearoyl-GPS (18:0)* | -0.05417 |
| beta-sitosterol | -0.046 | 1-oleoyl-2-linoleoyl-GPE (18:1/18:2)* | -0.05366 |
| 1-linolenoyl-GPC (18:3)* | -0.04597 | histamine | -0.05365 |
| guanine | -0.04583 | glutathione, oxidized (GSSG) | -0.05326 |
| campesterol | -0.0447 | gamma-glutamylfelinylglycine* | -0.05294 |
| 1-linoleoyl-GPI* (18:2)* | -0.04467 | sphingosine 1-phosphate | -0.05201 |
| 1-oleoyl-GPC (18:1) | -0.04447 | N-acetylneuraminate | -0.05183 |
| bilirubin (E,Z or Z,E)* | -0.04425 | 1-(1-enyl-palmitoyl)-2-arachidonoyl-GPE (P-16:0/20:4)* | -0.05171 |
| 1-palmitoyl-GPI* (16:0) | -0.04409 | 1-stearoyl-2-oleoyl-GPE (18:0/18:1) | -0.05148 |
| linoleoylcholine* | -0.04202 | felinylglycine* | -0.05095 |
| bilirubin (E,E)* | -0.04193 | sphingosine | -0.05092 |
| palmitoylcholine | -0.04167 | 1,2-dipalmitoyl-GPC (16:0/16:0) | -0.05036 |
| glycerophosphoethanolamine | -0.04129 | stearoyl-arachidonoyl-glycerol (18:0/20:4) [1]* | -0.0502 |
| arachidonate (20:4n6) | -0.04127 | linoleoyl-linoleoyl-glycerol (18:2/18:2) [1]* | -0.04971 |
| N-stearoylserine* | -0.04114 | 5alpha-pregnan-3beta,20beta-diol monosulfate (1) | -0.04956 |
| sphingomyelin (d18:2/14:0, d18:1/14:1)* | -0.04093 | sphingadienine | -0.04955 |
| 1-stearoyl-2-oleoyl-GPC (18:0/18:1) | -0.04057 | thymol sulfate | -0.04928 |
| alpha-tocopherol | -0.04055 | stearoyl-arachidonoyl-glycerol (18:0/20:4) [2]* | -0.04923 |
| 1-palmitoyl-GPC (16:0) | -0.04035 | linoleoyl-linolenoyl-glycerol (18:2/18:3) [2]* | -0.04867 |
| 1-palmitoleoyl-GPC* (16:1)* | -0.04035 | threonine | -0.04856 |
| 1-stearoyl-2-arachidonoyl-GPC (18:0/20:4) | -0.03955 | 1-methylnicotinamide | -0.04807 |
| 1,2-dilinoleoyl-GPC (18:2/18:2) | -0.03939 | 1-(1-enyl-stearoyl)-2-linoleoyl-GPE (P-18:0/18:2)* | -0.04725 |
| eicosanodioate (C20-DC) | -0.03923 | sphinganine-1-phosphate | -0.04635 |
| 2-aminoheptanoate | -0.03901 | 1-palmitoyl-2-oleoyl-GPE (16:0/18:1) | -0.04612 |
| biliverdin | -0.03896 | valine | -0.04578 |
| 2-palmitoyl-GPC* (16:0)* | -0.03884 | prolylhydroxyproline | -0.04535 |
| oleoylcholine | -0.03871 | ergothioneine | -0.04534 |
| nonadecanoate (19:0) | -0.0381 | palmitoyl-linoleoyl-glycerol (16:0/18:2) [1]* | -0.0451 |
| 2-aminooctanoate | -0.03793 | oleoyl-arachidonoyl-glycerol (18:1/20:4) [2]* | -0.04508 |
| stearoylcholine* | -0.03723 | sulfate* | -0.04487 |
| 1-(1-enyl-palmitoyl)-GPE (P-16:0)* | -0.03706 | nicotinate ribonucleoside | -0.04365 |
| asparagine | -0.03564 | serotonin | -0.04351 |
| docosahexaenoate (DHA; 22:6n3) | -0.0353 | N-acetyltaurine | -0.04279 |
| eicosapentaenoate (EPA; 20:5n3) | -0.03519 | linoleoyl-arachidonoyl-glycerol (18:2/20:4) [1]* | -0.04217 |
| docosadienoate (22:2n6) | -0.03501 | proline | -0.0417 |
| 1-myristoyl-2-arachidonoyl-GPC (14:0/20:4)* | -0.03474 | deoxycarnitine | -0.04153 |
| 1-(1-enyl-stearoyl)-GPE (P-18:0)* | -0.03437 | guanidinoacetate | -0.04125 |
| arachidate (20:0) | -0.03426 | gamma-glutamylleucine | -0.04083 |
| 2-hydroxylaurate | -0.03387 | 1-palmitoyl-2-linoleoyl-GPE (16:0/18:2) | -0.04058 |
| 1-stearoyl-2-linoleoyl-GPC (18:0/18:2)* | -0.03373 | felinine | -0.03966 |
| 2-aminobutyrate | -0.03371 | cysteinylglycine | -0.03908 |
| kynurenine | -0.03357 | creatine | -0.03883 |
| glucose | -0.0335 | phosphocholine | -0.03853 |
| 1-stearoyl-GPE (18:0) | -0.0334 | cysteine | -0.03801 |
| 1-linoleoyl-GPA (18:2)* | -0.03308 | acetylphosphate | -0.03793 |
| cysteine sulfinic acid | -0.03264 | prolylglycine | -0.03775 |
| erucate (22:1n9) | -0.0323 | alanine | -0.03749 |
| gluconate | -0.03208 | linoleoyl-arachidonoyl-glycerol (18:2/20:4) [2]* | -0.03734 |
| cholesterol | -0.03207 | stearoyl-linoleoyl-glycerol (18:0/18:2) [2]* | -0.03709 |
| 2-stearoyl-GPE (18:0)* | -0.03139 | glutamate | -0.03667 |
| 1-palmitoleoyl-2-linolenoyl-GPC (16:1/18:3)* | -0.03123 | gamma-glutamylthreonine | -0.03638 |
| 1-linoleoyl-2-linolenoyl-GPC (18:2/18:3)* | -0.03104 | nicotinamide | -0.03607 |
| nervonate (24:1n9)* | -0.03101 | spermidine | -0.03604 |
| carotene diol (1) | -0.03081 | heme | -0.03598 |
| glutamate | -0.03067 | N-linolenoyltaurine* | -0.03566 |
| docosatrienoate (22:3n6)* | -0.03043 | palmitoleoyl-linoleoyl-glycerol (16:1/18:2) [1]* | -0.03528 |
| laurate (12:0) | -0.03034 | diacylglycerol (16:1/18:2 [2], 16:0/18:3 [1])* | -0.03432 |
| 2-hydroxystearate | -0.02984 | palmitoyl-linoleoyl-glycerol (16:0/18:2) [2]* | -0.03406 |
| N-behenoyl-sphingadienine (d18:2/22:0)* | -0.02937 | palmitoyl-oleoyl-glycerol (16:0/18:1) [1]* | -0.03392 |
| N2,N2-dimethylguanosine | -0.02922 | sphinganine | -0.03236 |
| tyrosine | -0.02916 | imidazole lactate | -0.03216 |
| behenoyl dihydrosphingomyelin (d18:0/22:0)* | -0.02886 | methionine sulfoxide | -0.03173 |
| N-palmitoyl-sphingosine (d18:1/16:0) | -0.02831 | 5-aminovalerate | -0.03139 |
| octadecenedioate (C18:1-DC)* | -0.02818 | anthranilate | -0.03123 |
| threonine | -0.0278 | phenylacetylserine | -0.03064 |
| 10-undecenoate (11:1n1) | -0.02778 | oleoyl-linoleoyl-glycerol (18:1/18:2) [1] | -0.03035 |
| thymol sulfate | -0.02745 | carnosine | -0.03021 |
| sphingomyelin (d17:1/14:0, d16:1/15:0)* | -0.02728 | 1-palmitoyl-GPE (16:0) | -0.02974 |
| behenoyl sphingomyelin (d18:1/22:0)* | -0.0271 | cysteine s-sulfate | -0.02961 |
| 4-ethylphenyl sulfate | -0.02644 | 7-hydroxyindole sulfate | -0.02955 |
| docosapentaenoate (DPA; 22:5n3) | -0.02632 | 1-stearoyl-2-arachidonoyl-GPC (18:0/20:4) | -0.02948 |
| 1-palmitoyl-2-palmitoleoyl-GPC (16:0/16:1)* | -0.02599 | N-monomethylarginine | -0.02895 |
| 1-(1-enyl-oleoyl)-GPE (P-18:1)* | -0.02599 | homoarginine | -0.02889 |
| isoleucine | -0.02597 | asparagine | -0.02874 |
| leucine | -0.02586 | glucose | -0.0286 |
| palmitoyl dihydrosphingomyelin (d18:0/16:0)* | -0.02583 | gamma-glutamylmethionine | -0.02823 |
| 1-lignoceroyl-GPC (24:0) | -0.02565 | malate | -0.0281 |
| 2-hydroxypalmitate | -0.02563 | palmitoyl-oleoyl-glycerol (16:0/18:1) [2]* | -0.0281 |
| 2'-deoxycytidine | -0.0255 | gamma-glutamylglutamate | -0.02809 |
| 1-(1-enyl-stearoyl)-2-oleoyl-GPE (P-18:0/18:1) | -0.02547 | N-myristoyltaurine* | -0.02776 |
| margarate (17:0) | -0.02526 | gamma-glutamylvaline | -0.02775 |
| sphingomyelin (d17:2/16:0, d18:2/15:0)* | -0.02523 | glutaroylcarnitine (C5) | -0.02728 |
| 1-(1-enyl-palmitoyl)-GPC (P-16:0)* | -0.02507 | arginine | -0.02724 |
| stearoyl-linoleoyl-glycerol (18:0/18:2) [2]* | -0.02505 | phenylacetylglutamine | -0.02724 |
| tryptophan betaine | -0.02495 | 1-(1-enyl-palmitoyl)-2-oleoyl-GPE (P-16:0/18:1)* | -0.02703 |
| 1-stearoyl-2-oleoyl-GPI (18:0/18:1)* | -0.0249 | N-stearoyl-sphinganine (d18:0/18:0)* | -0.02694 |
| pantothenate (Vitamin B5) | -0.0249 | methionine | -0.02689 |
| ceramide (d18:2/24:1, d18:1/24:2)* | -0.02479 | 5-methylthioadenosine (MTA) | -0.02686 |
| ceramide (d18:1/14:0, d16:1/16:0)* | -0.02465 | 10-undecenoate (11:1n1) | -0.02608 |
| 1-oleoylglycerol (18:1) | -0.0246 | 1-stearoyl-2-arachidonoyl-GPI (18:0/20:4) | -0.02565 |
| 1-palmitoleoyl-2-linoleoyl-GPC (16:1/18:2)* | -0.02447 | methylnaphthyl sulfate (1)* | -0.02544 |
| stearate (18:0) | -0.02434 | oleoyl-linoleoyl-glycerol (18:1/18:2) [2] | -0.0252 |
| behenate (22:0)* | -0.02433 | 1-oleoyl-2-arachidonoyl-GPI (18:1/20:4) * | -0.02494 |
| adrenate (22:4n6) | -0.02428 | azelate (nonanedioate; C9) | -0.0249 |
| methylnaphthyl sulfate (1)* | -0.02412 | 2-piperidinone | -0.02481 |
| docosapentaenoate (n6 DPA; 22:5n6) | -0.0238 | N4-acetylcytidine | -0.02455 |
| 1-palmitoyl-2-stearoyl-GPC (16:0/18:0) | -0.02378 | diacylglycerol (14:0/18:1, 16:0/16:1) [2]* | -0.02452 |
| N6-methyladenosine | -0.02374 | 4-methyl-2-oxopentanoate | -0.02436 |
| N-acetyl-aspartyl-glutamate (NAAG) | -0.02372 | S-adenosylhomocysteine (SAH) | -0.02432 |
| pyridoxal | -0.0236 | 4-hydroxycinnamate sulfate | -0.0243 |
| fructose | -0.02359 | indolepropionate | -0.02403 |
| octadecanedioate (C18) | -0.02329 | laurate (12:0) | -0.02385 |
| N-palmitoyl-sphingadienine (d18:2/16:0)* | -0.02289 | dimethylglycine | -0.02385 |
| 1-oleoyl-2-linoleoyl-GPC (18:1/18:2)* | -0.02289 | diacylglycerol (14:0/18:1, 16:0/16:1) [1]* | -0.0237 |
| 2,4-dichlorophenol sulfate | -0.02277 | 2-aminobutyrate | -0.02358 |
| linoleoyl-arachidonoyl-glycerol (18:2/20:4) [2]* | -0.02274 | 1-(1-enyl-stearoyl)-2-oleoyl-GPE (P-18:0/18:1) | -0.02332 |
| 3-methoxycatechol sulfate (1) | -0.02229 | methylnaphthyl sulfate (2)* | -0.02331 |
| ergothioneine | -0.02219 | ornithine | -0.02324 |
| lignoceroyl sphingomyelin (d18:1/24:0) | -0.02213 | cysteinylglycine disulfide* | -0.02321 |
| 1-(1-enyl-stearoyl)-2-linoleoyl-GPE (P-18:0/18:2)* | -0.02206 | 1-stearoyl-2-docosahexaenoyl-GPC (18:0/22:6) | -0.0231 |
| linoleoyl-arachidonoyl-glycerol (18:2/20:4) [1]* | -0.02204 | alpha-hydroxyisocaproate | -0.02288 |
| N-palmitoyl-sphinganine (d18:0/16:0) | -0.02167 | gamma-glutamylalanine | -0.0225 |
| 1-myristoyl-2-palmitoyl-GPC (14:0/16:0) | -0.02149 | indoleacetylcarnitine* | -0.02234 |
| sphingomyelin (d18:0/20:0, d16:0/22:0)* | -0.02136 | 1-(1-enyl-stearoyl)-GPE (P-18:0)* | -0.02221 |
| myristoyl dihydrosphingomyelin (d18:0/14:0)* | -0.02098 | 3-hydroxybutyrate (BHBA) | -0.02215 |
| glycosyl ceramide (d18:1/23:1, d17:1/24:1)* | -0.02072 | 12,13-DiHOME | -0.02213 |
| diacylglycerol (16:1/18:2 [2], 16:0/18:3 [1])* | -0.02056 | indolelactate | -0.02208 |
| alpha-ketobutyrate | -0.02054 | pipecolate | -0.02188 |
| nicotinate ribonucleoside | -0.02016 | 1-myristoyl-2-palmitoyl-GPC (14:0/16:0) | -0.02174 |
| linolenate (18:3n3 or 3n6) | -0.02006 | homocitrulline | -0.02075 |
| glycosyl-N-stearoyl-sphinganine (d18:0/18:0)* | -0.02001 | 3-(4-hydroxyphenyl)propionate | -0.02048 |
| glycerophosphoinositol* | -0.01978 | homocystine | -0.02037 |
| thymidine | -0.01962 | 3-hydroxyindolin-2-one sulfate | -0.02031 |
| N-acetylleucine | -0.01954 | N-acetylcarnosine | -0.0202 |
| gamma-tocopherol/beta-tocopherol | -0.01948 | arabitol/xylitol | -0.02007 |
| hypoxanthine | -0.0194 | gamma-glutamylisoleucine* | -0.01995 |
| palmitoleate (16:1n7) | -0.01925 | threonate | -0.01981 |
| hydroquinone sulfate | -0.01914 | 5-hydroxyindole sulfate | -0.01918 |
| linoleate (18:2n6) | -0.01907 | ferulic acid 4-sulfate | -0.01903 |
| retinal | -0.01898 | creatinine | -0.01896 |
| ceramide (d18:1/17:0, d17:1/18:0)* | -0.01884 | N-acetyl-aspartyl-glutamate (NAAG) | -0.01893 |
| taurocholate | -0.01875 | S-1-pyrroline-5-carboxylate | -0.01868 |
| dihomolinoleate (20:2n6) | -0.01874 | cortisol | -0.01862 |
| 1-stearoyl-2-arachidonoyl-GPI (18:0/20:4) | -0.01859 | 2-aminoadipate | -0.01837 |
| caprate (10:0) | -0.01859 | 2-methylserine | -0.01824 |
| 3-(3-hydroxyphenyl)propionate | -0.01854 | 4-hydroxyphenylpyruvate | -0.01799 |
| phenylalanine | -0.01852 | glycerophosphoethanolamine | -0.01794 |
| proline | -0.01821 | malonate | -0.01736 |
| betaine | -0.0182 | 1-oleoyl-2-docosahexaenoyl-GPC (18:1/22:6)* | -0.01736 |
| heneicosapentaenoate (21:5n3) | -0.01815 | cholate | -0.01727 |
| taurochenodeoxycholate | -0.01807 | 4-guanidinobutanoate | -0.01693 |
| stearidonate (18:4n3) | -0.01759 | isovalerylglycine | -0.01684 |
| cytidine | -0.01758 | N-acetyl-cadaverine | -0.01635 |
| palmitoleoyl-linoleoyl-glycerol (16:1/18:2) [1]* | -0.01741 | 6-hydroxyindole sulfate | -0.01631 |
| linoleoyl-linolenoyl-glycerol (18:2/18:3) [2]* | -0.01719 | beta-hydroxyisovalerate | -0.01608 |
| guaiacol sulfate | -0.01711 | 3-hydroxypyridine sulfate | -0.01591 |
| thyroxine | -0.01703 | N-acetylleucine | -0.01587 |
| docosatrienoate (22:3n3) | -0.01686 | phenylacetylalanine | -0.01562 |
| hexadecenedioate (C16:1-DC)* | -0.01674 | 1-methylhistamine | -0.0156 |
| 1-(1-enyl-palmitoyl)-2-oleoyl-GPE (P-16:0/18:1)* | -0.01646 | phenylacetate | -0.01541 |
| palmitoyl-linoleoyl-glycerol (16:0/18:2) [2]* | -0.01642 | AMP | -0.0151 |
| guanosine | -0.01626 | ethylmalonate | -0.01509 |
| N-palmitoylglycine | -0.01619 | docosadioate (C22-DC) | -0.01502 |
| ethylmalonate | -0.01593 | 6-oxopiperidine-2-carboxylate | -0.01497 |
| 1-palmitoyl-2-oleoyl-GPI (16:0/18:1)* | -0.01579 | oxalate (ethanedioate) | -0.01489 |
| diacylglycerol (14:0/18:1, 16:0/16:1) [1]* | -0.01559 | N2,N2-dimethylguanine | -0.01468 |
| iminodiacetate (IDA) | -0.01549 | 3-indoxyl sulfate | -0.01458 |
| taurolithocholate 3-sulfate | -0.01544 | 1-methyladenosine | -0.01456 |
| heme | -0.01523 | 4-hydroxyphenylacetate sulfate | -0.01432 |
| taurocholenate sulfate | -0.01494 | heptanoate (7:0) | -0.01422 |
| tricosanoyl sphingomyelin (d18:1/23:0)* | -0.01465 | 4-hydroxyhippurate | -0.01389 |
| oleoyl-linoleoyl-glycerol (18:1/18:2) [2] | -0.01462 | 12-HETE | -0.01368 |
| cystine | -0.01449 | 2-oxoarginine* | -0.01342 |
| choline | -0.01446 | behenoylcarnitine (C22)* | -0.01305 |
| hexadecadienoate (16:2n6) | -0.01434 | gluconate | -0.01288 |
| taurodeoxycholate | -0.01424 | 4-hydroxyphenylacetate | -0.01273 |
| 5-methyl-2'-deoxycytidine | -0.01424 | 4-methylcatechol sulfate | -0.01239 |
| palmitoyl sphingomyelin (d18:1/16:0) | -0.0142 | 5-dodecenoate (12:1n7) | -0.01234 |
| eicosenoate (20:1n9 or 1n11) | -0.01419 | N-acetylglucosaminylasparagine | -0.01233 |
| 2-hydroxyglutarate | -0.01416 | 1-palmitoyl-2-arachidonoyl-GPI (16:0/20:4)* | -0.01219 |
| uridine | -0.01404 | N-linoleoyltaurine* | -0.01208 |
| diacylglycerol (14:0/18:1, 16:0/16:1) [2]* | -0.01345 | gamma-glutamylhistidine | -0.01207 |
| sphingomyelin (d18:2/23:0, d18:1/23:1, d17:1/24:1)* | -0.01338 | linolenate (18:3n3 or 3n6) | -0.01206 |
| myristate (14:0) | -0.01296 | mannitol/sorbitol | -0.01197 |
| N-linolenoyltaurine* | -0.01295 | myo-inositol | -0.0118 |
| N-stearoyl-sphingosine (d18:1/18:0)* | -0.01275 | N-acetylfelinine* | -0.01151 |
| 1-stearoyl-2-linoleoyl-GPI (18:0/18:2) | -0.0126 | N-arachidonoyltaurine | -0.01146 |
| 10-nonadecenoate (19:1n9) | -0.01259 | pyrraline | -0.01129 |
| homoarginine | -0.01255 | 2-hydroxyoctanoate | -0.0112 |
| cortisol | -0.01255 | 1-palmitoyl-2-alpha-linolenoyl-GPC (16:0/18:3n3)* | -0.0111 |
| pentadecanoate (15:0) | -0.01248 | alpha-hydroxyisovalerate | -0.01067 |
| methionine | -0.01247 | 1-oleoyl-2-linoleoyl-GPI (18:1/18:2)* | -0.01029 |
| hexadecanedioate (C16) | -0.01244 | docosatrienoate (22:3n3) | -0.01015 |
| alanine | -0.01224 | 4-hydroxyphenylacetylglycine | -0.00998 |
| xylose | -0.01221 | N-acetylaspartate (NAA) | -0.00997 |
| cysteine s-sulfate | -0.01218 | phenylacetylcarnitine | -0.0097 |
| linoleoyl ethanolamide | -0.01204 | cysteine sulfinic acid | -0.00954 |
| 4-methyl-2-oxopentanoate | -0.01204 | xylose | -0.00954 |
| gamma-glutamylfelinylglycine* | -0.01196 | oleoyl-oleoyl-glycerol (18:1/18:1) [1]* | -0.00941 |
| guanidinoacetate | -0.01182 | eugenol sulfate | -0.00929 |
| 2-hydroxydecanoate | -0.01175 | pyroglutamine* | -0.00923 |
| glycosyl ceramide (d18:1/20:0, d16:1/22:0)* | -0.01166 | lanthionine | -0.00891 |
| 1-stearoyl-2-oleoyl-GPE (18:0/18:1) | -0.01159 | trimethylamine N-oxide | -0.00883 |
| oleate/vaccenate (18:1) | -0.01156 | 2-aminooctanoate | -0.00882 |
| sphingomyelin (d18:1/17:0, d17:1/18:0, d19:1/16:0) | -0.01154 | 3-methyl-2-oxovalerate | -0.00881 |
| 4-imidazoleacetate | -0.01138 | 3-(4-hydroxyphenyl)lactate (HPLA) | -0.00866 |
| sphingomyelin (d18:0/18:0, d19:0/17:0)* | -0.01137 | retinol (Vitamin A) | -0.00858 |
| alpha-CEHC sulfate | -0.01132 | 2-isopropylmalate | -0.00849 |
| ceramide (d18:1/20:0, d16:1/22:0, d20:1/18:0)* | -0.01131 | benzoate | -0.00848 |
| salicylate | -0.0112 | 4-vinylguaiacol sulfate | -0.00847 |
| palmitoyl-linoleoyl-glycerol (16:0/18:2) [1]* | -0.01119 | 5-hydroxylysine | -0.00838 |
| carotene diol (3) | -0.01112 | 1-(1-enyl-oleoyl)-GPE (P-18:1)* | -0.00833 |
| hexadecatrienoate (16:3n3) | -0.01102 | 1-palmitoyl-2-stearoyl-GPC (16:0/18:0) | -0.00826 |
| arginine | -0.01097 | serine | -0.00825 |
| histamine | -0.01097 | eicosanodioate (C20-DC) | -0.00819 |
| 1-palmitoyl-2-arachidonoyl-GPC (16:0/20:4n6) | -0.01085 | 2,3-dihydroxyisovalerate | -0.00813 |
| ceramide (d16:1/24:1, d18:1/22:1)* | -0.0107 | lactate | -0.0081 |
| saccharin | -0.01063 | indoleacetate | -0.00808 |
| 1,2-dipalmitoyl-GPC (16:0/16:0) | -0.0106 | 5-methylcytosine | -0.00794 |
| S-methylmethionine | -0.01046 | O-sulfo-L-tyrosine | -0.00731 |
| malate | -0.01045 | 2-oxoadipate | -0.00724 |
| methyl-4-hydroxybenzoate sulfate | -0.01043 | hexadecatrienoate (16:3n3) | -0.00719 |
| methylnaphthyl sulfate (2)* | -0.01028 | isovalerate (C5) | -0.00698 |
| 1-stearoyl-2-docosahexaenoyl-GPC (18:0/22:6) | -0.01016 | 1-myristoyl-2-arachidonoyl-GPC (14:0/20:4)* | -0.00698 |
| 2-methylserine | -0.01012 | adenine | -0.00695 |
| phosphate | -0.00986 | EDTA | -0.00687 |
| inosine | -0.00966 | phenylacetylglycine | -0.00686 |
| EDTA | -0.00954 | 1-palmitoyl-2-arachidonoyl-GPC (16:0/20:4n6) | -0.00649 |
| 3-ureidoisobutyrate | -0.00951 | 5-(galactosylhydroxy)-L-lysine | -0.00641 |
| N-stearoyltaurine | -0.00942 | 2-stearoyl-GPE (18:0)* | -0.0063 |
| glycosyl-N-palmitoyl-sphingosine (d18:1/16:0) | -0.00923 | orotate | -0.00609 |
| sarcosine | -0.00919 | cystine | -0.00607 |
| sphingomyelin (d18:2/21:0, d16:2/23:0)* | -0.00915 | phosphate | -0.00596 |
| 1-stearoyl-GPS (18:0)* | -0.00915 | anserine | -0.00581 |
| sedoheptulose | -0.00905 | alpha-ketobutyrate | -0.00577 |
| felinylglycine* | -0.00893 | 3-hydroxyisobutyrate | -0.00568 |
| sphingomyelin (d18:1/21:0, d17:1/22:0, d16:1/23:0)* | -0.00877 | isobutyrylglycine (C4) | -0.00554 |
| 1-stearoyl-2-linoleoyl-GPE (18:0/18:2)* | -0.00856 | gamma-glutamylglutamine | -0.00519 |
| glycosyl-N-stearoyl-sphingosine (d18:1/18:0) | -0.0085 | N-methylalanine | -0.00517 |
| lactosyl-N-palmitoyl-sphingosine (d18:1/16:0) | -0.00848 | N-palmitoyltaurine | -0.00511 |
| oleoyl-arachidonoyl-glycerol (18:1/20:4) [2]* | -0.00834 | 2-hydroxy-3-methylvalerate | -0.00508 |
| sphingomyelin (d18:1/14:0, d16:1/16:0)* | -0.00828 | 3-phenylpropionate (hydrocinnamate) | -0.00504 |
| oleoyl-linoleoyl-glycerol (18:1/18:2) [1] | -0.00823 | 13-HODE + 9-HODE | -0.00487 |
| 1-palmitoyl-2-alpha-linolenoyl-GPC (16:0/18:3n3)* | -0.00793 | 4-imidazoleacetate | -0.00476 |
| sphingomyelin (d18:1/24:1, d18:2/24:0)* | -0.00786 | 1-palmitoyl-2-docosahexaenoyl-GPC (16:0/22:6) | -0.00454 |
| chenodeoxycholate | -0.00771 | 1-stearoyl-2-linoleoyl-GPI (18:0/18:2) | -0.00452 |
| heptanoate (7:0) | -0.0077 | 1,2,3-benzenetriol sulfate (2) | -0.00441 |
| sphinganine | -0.00762 | propionylcarnitine (C3) | -0.00429 |
| 17-methylstearate (i19:0) | -0.00758 | N-acetylisoleucine | -0.00406 |
| sphingomyelin (d17:1/16:0, d18:1/15:0, d16:1/17:0)* | -0.00755 | equol sulfate | -0.004 |
| sphingosine 1-phosphate | -0.00753 | 3-hydroxymyristate | -0.00391 |
| oleoyl-oleoyl-glycerol (18:1/18:1) [2]* | -0.00747 | dimethylarginine (ADMA + SDMA) | -0.0038 |
| sphingomyelin (d18:2/18:1)* | -0.00741 | thymidine | -0.00357 |
| mannitol/sorbitol | -0.00736 | maleate | -0.00331 |
| 1-palmitoyl-2-oleoyl-GPC (16:0/18:1) | -0.00735 | valerate (5:0) | -0.00309 |
| palmitoyl-oleoyl-glycerol (16:0/18:1) [2]* | -0.00724 | mevalonate | -0.00308 |
| glycosyl ceramide (d16:1/24:1, d18:1/22:1)* | -0.00716 | dihydroorotate | -0.00299 |
| sphinganine-1-phosphate | -0.007 | caprate (10:0) | -0.00275 |
| N-acetyl-cadaverine | -0.00695 | betaine | -0.00269 |
| 10-heptadecenoate (17:1n7) | -0.00687 | oleoyl-oleoyl-glycerol (18:1/18:1) [2]* | -0.00262 |
| 1-linoleoyl-2-arachidonoyl-GPE (18:2/20:4)* | -0.00687 | caproate (6:0) | -0.00249 |
| palmitate (16:0) | -0.00659 | 1-palmitoyl-2-linoleoyl-GPI (16:0/18:2) | -0.00248 |
| aspartate | -0.00645 | N-delta-acetylornithine | -0.00246 |
| oleoyl-oleoyl-glycerol (18:1/18:1) [1]* | -0.00644 | 1-stearoyl-GPE (18:0) | -0.00239 |
| formiminoglutamate | -0.00613 | S-methylmethionine | -0.00205 |
| 9,10-DiHOME | -0.006 | cinnamoylglycine | -0.00204 |
| 1-methylhistamine | -0.00598 | tryptophan | -0.00171 |
| cytosine | -0.00597 | hippurate | -0.00159 |
| 1-stearoyl-2-arachidonoyl-GPE (18:0/20:4) | -0.00563 | nicotinamide riboside | -0.00147 |
| 1-oleoyl-2-linoleoyl-GPI (18:1/18:2)* | -0.00553 | picolinate | -0.00143 |
| nicotinamide | -0.00539 | aconitate [cis or trans] | -0.00134 |
| 3-methyl-2-oxovalerate | -0.00527 | pyridoxamine | -0.00105 |
| N-stearoyl-sphinganine (d18:0/18:0)* | -0.00527 | imidazole propionate | -0.00064 |
| serotonin | -0.00524 | glycerophosphoglycerol | -0.00063 |
| glucuronate | -0.00514 | chenodeoxycholate | -0.00055 |
| 15-methylpalmitate (i17:0) | -0.00497 | 4-ethylphenyl sulfate | -0.00051 |
| 1-stearoyl-2-arachidonoyl-GPS (18:0/20:4) | -0.0047 | phenyllactate (PLA) | -0.0002 |
| maleate | -0.00465 | carotene diol (2) | -0.00015 |
| 1-(1-enyl-stearoyl)-2-arachidonoyl-GPE (P-18:0/20:4)* | -0.00449 | C-glycosyltryptophan | -0.00006 |
| glycosyl-N-(2-hydroxynervonoyl)-sphingosine (d18:1/24:1(2OH))* | -0.0044 | tryptophan betaine | -0.00002 |
| 12,13-DiHOME | -0.00416 | phenylpropionylglycine | 0.0001 |
| N-myristoyltaurine* | -0.00402 | dodecanedioate (C12) | 0.00039 |
| N-oleoylserine | -0.00401 | choline | 0.00049 |
| ribitol | -0.00383 | glycerate | 0.0005 |
| lignoceroylcarnitine (C24)* | -0.00379 | 2-hydroxylaurate | 0.00052 |
| inosine 5'-monophosphate (IMP) | -0.00377 | phenylacetylglutamate | 0.00058 |
| tetradecanedioate (C14) | -0.00349 | 2-hydroxybutyrate/2-hydroxyisobutyrate | 0.00064 |
| carotene diol (2) | -0.00315 | erythritol | 0.00081 |
| 1-palmitoyl-2-arachidonoyl-GPI (16:0/20:4)* | -0.00309 | gamma-tocopherol/beta-tocopherol | 0.00082 |
| sphingomyelin (d18:2/23:1)* | -0.00297 | 2-hydroxyphenylacetate | 0.00085 |
| 5-aminovalerate | -0.00296 | salicylate | 0.00092 |
| glutamine | -0.00273 | tyrosine | 0.00105 |
| 1-stearoyl-2-linoleoyl-GPS (18:0/18:2) | -0.00272 | 4-acetylphenyl sulfate | 0.00126 |
| 3-ureidopropionate | -0.00255 | 1,5-anhydroglucitol (1,5-AG) | 0.00138 |
| N-acetylaspartate (NAA) | -0.00254 | mevalonolactone | 0.00149 |
| sphingosine | -0.00251 | 3-acetylphenol sulfate | 0.00158 |
| sphingadienine | -0.00238 | N-oleoyltaurine | 0.00166 |
| palmitoyl-oleoyl-glycerol (16:0/18:1) [1]* | -0.00232 | 12-HHTrE | 0.00169 |
| valine | -0.00221 | 3-methylhistidine | 0.0018 |
| eugenol sulfate | -0.00207 | hydroxyproline | 0.00211 |
| thromboxane B2 | -0.00194 | citrate | 0.00216 |
| gamma-glutamylmethionine | -0.00185 | dodecadienoate (12:2)* | 0.00224 |
| indolepropionate | -0.00136 | N6-methyladenosine | 0.00234 |
| stearoyl-arachidonoyl-glycerol (18:0/20:4) [2]* | -0.00121 | octadecanedioate (C18) | 0.00234 |
| 1-palmitoyl-2-linoleoyl-GPC (16:0/18:2) | -0.00117 | 3-hydroxybutyrylcarnitine (2) | 0.00258 |
| 12-HETE | -0.00102 | erythronate* | 0.00297 |
| 2'-deoxyuridine | -0.0008 | alpha-ketoglutarate | 0.00302 |
| phosphoethanolamine (PE) | -0.00071 | stearidonate (18:4n3) | 0.00304 |
| thioproline | -0.00046 | urea | 0.00312 |
| xanthine | -0.00031 | hypoxanthine | 0.00313 |
| desmosterol | -0.00029 | guanidinosuccinate | 0.00327 |
| 2'-O-methylcytidine | -0.00021 | gamma-glutamyltyrosine | 0.00331 |
| alpha-hydroxyisocaproate | 0.00023 | N6-acetyllysine | 0.00346 |
| 3-phosphoglycerate | 0.00023 | sebacate (C10-DC) | 0.00351 |
| sphingomyelin (d18:1/22:1, d18:2/22:0, d16:1/24:1)* | 0.00029 | (N(1) + N(8))-acetylspermidine | 0.00379 |
| 5-methylcytosine | 0.00031 | S-methylcysteine sulfoxide | 0.00381 |
| 1-oleoyl-GPE (18:1) | 0.00032 | 5-bromotryptophan | 0.00384 |
| N-acetylkynurenine (2) | 0.00032 | 1-methylguanidine | 0.00436 |
| 4-vinylguaiacol sulfate | 0.00039 | N-acetylarginine | 0.00442 |
| 1-palmitoyl-2-linoleoyl-GPI (16:0/18:2) | 0.00048 | 3-(3-hydroxyphenyl)propionate | 0.00447 |
| 5-hydroxymethyl-2'-deoxycytidine | 0.0006 | riboflavin (Vitamin B2) | 0.00453 |
| 3-aminoisobutyrate | 0.00074 | 3-methoxycatechol sulfate (1) | 0.00463 |
| 4-methoxyphenol sulfate | 0.00088 | N-stearoyltaurine | 0.00468 |
| phenylpyruvate | 0.00101 | beta-alanine | 0.00469 |
| gamma-glutamylalanine | 0.00109 | 3-hydroxy-3-methylglutarate | 0.00469 |
| stearoyl-arachidonoyl-glycerol (18:0/20:4) [1]* | 0.00137 | orotidine | 0.00478 |
| 1-methylnicotinamide | 0.00138 | N-palmitoleoyltaurine* | 0.005 |
| acetylphosphate | 0.0015 | adipoylcarnitine (C6-DC) | 0.00505 |
| anthranilate | 0.00166 | N-acetylalanine | 0.00575 |
| S-1-pyrroline-5-carboxylate | 0.00166 | N-acetyltryptophan | 0.00611 |
| cholate | 0.00182 | 2,4-dichlorophenol sulfate | 0.00612 |
| S-methylcysteine sulfoxide | 0.00183 | docosatrienoate (22:3n6)* | 0.00612 |
| AMP | 0.00183 | indoleacetylglutamine | 0.00616 |
| sphingomyelin (d18:1/20:2, d18:2/20:1, d16:1/22:2)* | 0.00211 | 1-arachidonoyl-GPE* (20:4)* | 0.00663 |
| linoleoyl-linoleoyl-glycerol (18:2/18:2) [1]* | 0.00212 | methyl indole-3-acetate | 0.00663 |
| pyridoxamine | 0.00224 | benzoylcarnitine* | 0.00689 |
| 1-(1-enyl-palmitoyl)-2-arachidonoyl-GPE (P-16:0/20:4)* | 0.00224 | gamma-glutamyltryptophan | 0.00699 |
| 1-stearoyl-2-oleoyl-GPS (18:0/18:1) | 0.00224 | 2-hydroxydecanoate | 0.00712 |
| 2'-O-methyluridine | 0.00253 | myristate (14:0) | 0.00715 |
| 1-(1-enyl-palmitoyl)-2-arachidonoyl-GPC (P-16:0/20:4)* | 0.00262 | 2-aminophenol sulfate | 0.00722 |
| beta-alanine | 0.00269 | 3-methyl-2-oxobutyrate | 0.00731 |
| deoxycholate | 0.00288 | 3-aminoisobutyrate | 0.00739 |
| 12-HHTrE | 0.00297 | gamma-glutamylglycine | 0.00741 |
| ferulic acid 4-sulfate | 0.00318 | sarcosine | 0.00777 |
| cystathionine | 0.00321 | methionine sulfone | 0.008 |
| methionine sulfoxide | 0.0033 | hexadecadienoate (16:2n6) | 0.00823 |
| 1-oleoyl-2-arachidonoyl-GPI (18:1/20:4) * | 0.00359 | 15-methylpalmitate (i17:0) | 0.00854 |
| uracil | 0.00373 | adrenate (22:4n6) | 0.00854 |
| 3-hydroxyhippurate | 0.00377 | octadecenedioate (C18:1-DC)* | 0.00887 |
| succinate | 0.00378 | ribitol | 0.00911 |
| 4-hydroxyphenylacetate | 0.00386 | propionylglycine (C3) | 0.00919 |
| adenosine | 0.00428 | 1-ribosyl-imidazoleacetate* | 0.00931 |
| 3-(4-hydroxyphenyl)propionate | 0.00445 | 5-hydroxymethylcytidine | 0.00934 |
| alpha-ketoglutarate | 0.00452 | 2-oxindole-3-acetate | 0.00944 |
| 5-dodecenoate (12:1n7) | 0.00491 | hydroxyasparagine | 0.00963 |
| dimethylglycine | 0.00515 | N-acetylvaline | 0.00969 |
| methyl indole-3-acetate | 0.00519 | 5-hydroxyindoleacetate | 0.00971 |
| docosapentaenoylcarnitine (C22:5n3)* | 0.00534 | 16-hydroxypalmitate | 0.00974 |
| tartronate (hydroxymalonate) | 0.0054 | N-acetyl-beta-alanine | 0.01055 |
| 1-oleoyl-2-linoleoyl-GPE (18:1/18:2)* | 0.00546 | hexanoylglycine (C6) | 0.01064 |
| oleoyl ethanolamide | 0.00576 | ectoine | 0.01102 |
| 1,2-dilinoleoyl-GPE (18:2/18:2)* | 0.0058 | thromboxane B2 | 0.01104 |
| sphingomyelin (d18:1/18:1, d18:2/18:0) | 0.0058 | 5,6-dihydrouridine | 0.01123 |
| indolelactate | 0.00592 | myristoleate (14:1n5) | 0.01133 |
| N-arachidonoyltaurine | 0.00594 | thyroxine | 0.01137 |
| 3-methoxycatechol sulfate (2) | 0.00614 | 1-palmitoyl-2-oleoyl-GPI (16:0/18:1)* | 0.01146 |
| cysteine-glutathione disulfide | 0.00615 | 1-methylhistidine | 0.01158 |
| catechol sulfate | 0.00652 | N-acetylthreonine | 0.01162 |
| sphingomyelin (d18:1/19:0, d19:1/18:0)* | 0.00692 | catechol sulfate | 0.01182 |
| N-glycolylneuraminate | 0.00692 | 1-palmitoyl-2-palmitoleoyl-GPC (16:0/16:1)* | 0.01195 |
| myristoleate (14:1n5) | 0.00701 | lysine | 0.01205 |
| 1-palmitoyl-2-oleoyl-GPE (16:0/18:1) | 0.00703 | N-acetyl-isoputreanine* | 0.01225 |
| 1-arachidonoyl-GPE* (20:4)* | 0.00709 | caprylate (8:0) | 0.01227 |
| pyrraline | 0.00725 | palmitoleate (16:1n7) | 0.01229 |
| N-palmitoyltaurine | 0.00731 | 1-methyl-4-imidazoleacetate | 0.01252 |
| glycosyl-N-behenoyl-sphingadienine (d18:2/22:0)* | 0.00732 | ribonate (ribonolactone) | 0.01263 |
| 2-oxoadipate | 0.00738 | N-acetylglycine | 0.01271 |
| 5-hydroxylysine | 0.00739 | 3-methoxycatechol sulfate (2) | 0.01276 |
| butyrylglycine (C4) | 0.0074 | N-formylmethionine | 0.0128 |
| 1-stearoyl-GPG (18:0) | 0.00764 | N-palmitoyl-sphinganine (d18:0/16:0) | 0.01284 |
| sphingomyelin (d18:2/16:0, d18:1/16:1)* | 0.00824 | p-cresol sulfate | 0.01301 |
| glycerate | 0.00842 | butyrylglycine (C4) | 0.01302 |
| taurine | 0.00844 | fructose | 0.01303 |
| equol sulfate | 0.00849 | dihomolinolenate (20:3n3 or 3n6) | 0.01311 |
| phosphocholine | 0.0085 | 3-(3-hydroxyphenyl)propionate sulfate | 0.01353 |
| N-acetylneuraminate | 0.00854 | hydroquinone sulfate | 0.01359 |
| N-linoleoyltaurine* | 0.00884 | isobutyrylcarnitine (C4) | 0.01361 |
| 4-cholesten-3-one | 0.00902 | 4-methoxyphenol sulfate | 0.01364 |
| 13-HODE + 9-HODE | 0.00909 | indoleacetylglycine | 0.01388 |
| ornithine | 0.00914 | tiglyl carnitine (C5) | 0.01407 |
| glycerophosphoglycerol | 0.00929 | glutamine | 0.01409 |
| 3-hydroxypyridine sulfate | 0.00933 | 3-hydroxyhexanoate | 0.01425 |
| sphingomyelin (d18:2/24:2)* | 0.00933 | N-acetylphenylalanine | 0.0143 |
| carboxyethyl-GABA | 0.00935 | guanine | 0.01435 |
| phenyllactate (PLA) | 0.00947 | ophthalmate | 0.01449 |
| sphingomyelin (d18:1/20:0, d16:1/22:0)* | 0.00956 | pseudouridine | 0.0145 |
| caprylate (8:0) | 0.00963 | dopamine 3-O-sulfate | 0.01477 |
| 2-hydroxyphenylacetate | 0.01005 | N6-carbamoylthreonyladenosine | 0.0148 |
| 1-palmitoyl-2-arachidonoyl-GPE (16:0/20:4)* | 0.01011 | cystathionine | 0.0149 |
| 4-hydroxyhippurate | 0.01022 | N-acetylserine | 0.01492 |
| histidine | 0.01029 | biliverdin | 0.01495 |
| prolylglycine | 0.01032 | azeloylcarnitine (C9-DC) | 0.01522 |
| lactate | 0.01033 | S-methylcysteine | 0.01527 |
| 1-oleoyl-2-docosahexaenoyl-GPC (18:1/22:6)* | 0.01037 | argininate* | 0.01529 |
| sphingomyelin (d18:2/24:1, d18:1/24:2)* | 0.01069 | 3-hydroxyphenylacetate sulfate | 0.01531 |
| sphingomyelin (d18:1/22:2, d18:2/22:1, d16:1/24:2)* | 0.01088 | cortisone | 0.01538 |
| 1-palmitoyl-2-docosahexaenoyl-GPC (16:0/22:6) | 0.01103 | palmitate (16:0) | 0.01555 |
| 5alpha-pregnan-3beta,20beta-diol monosulfate (1) | 0.01111 | margarate (17:0) | 0.01574 |
| phenylpropionylglycine | 0.01128 | sedoheptulose | 0.01578 |
| 1-linoleoyl-GPE (18:2)* | 0.01157 | behenate (22:0)* | 0.01605 |
| 2-aminophenol sulfate | 0.01167 | 1-stearoyl-2-oleoyl-GPI (18:0/18:1)* | 0.01607 |
| glycosyl ceramide (d18:2/24:1, d18:1/24:2)* | 0.01171 | carotene diol (3) | 0.01613 |
| 3-(3-hydroxyphenyl)propionate sulfate | 0.01205 | N1-Methyl-2-pyridone-5-carboxamide | 0.01629 |
| dopamine 3-O-sulfate | 0.01243 | glycine | 0.01632 |
| 4-acetylphenyl sulfate | 0.01248 | N-acetylhistamine | 0.01637 |
| N-acetylhistamine | 0.01269 | mannose | 0.01641 |
| dihydroorotate | 0.01284 | linoleate (18:2n6) | 0.01649 |
| spermidine | 0.01333 | acetylcarnitine (C2) | 0.01658 |
| 3-(4-hydroxyphenyl)lactate (HPLA) | 0.01363 | erucate (22:1n9) | 0.01667 |
| gamma-glutamyltyrosine | 0.01388 | suberate (C8-DC) | 0.01673 |
| cerotoylcarnitine (C26)* | 0.01429 | lactosyl-N-palmitoyl-sphingosine (d18:1/16:0) | 0.01674 |
| indoleacetate | 0.01438 | pantothenate (Vitamin B5) | 0.0169 |
| cinnamoylglycine | 0.01449 | heneicosapentaenoate (21:5n3) | 0.01703 |
| 1-palmitoyl-GPG (16:0)* | 0.01465 | 1-(1-enyl-palmitoyl)-GPE (P-16:0)* | 0.01726 |
| pyruvate | 0.01472 | N6,N6,N6-trimethyllysine | 0.01738 |
| nervonoylcarnitine (C24:1)* | 0.01498 | 1-linoleoyl-GPA (18:2)* | 0.01753 |
| N-methylalanine | 0.015 | dihomolinoleate (20:2n6) | 0.01763 |
| felinine | 0.01518 | 2-methylmalonylcarnitine (C4-DC) | 0.01767 |
| 1-(1-enyl-palmitoyl)-2-linoleoyl-GPC (P-16:0/18:2)* | 0.01631 | gamma-glutamylphenylalanine | 0.01768 |
| 1-palmitoyl-2-docosahexaenoyl-GPE (16:0/22:6)* | 0.01636 | methyl-4-hydroxybenzoate sulfate | 0.01793 |
| 4-hydroxycinnamate sulfate | 0.01637 | pyridoxate | 0.01796 |
| 3-hydroxymyristate | 0.01664 | 3-hydroxy-2-ethylpropionate | 0.0182 |
| propionylglycine (C3) | 0.01691 | 3-hydroxydecanoate | 0.01822 |
| octadecenedioylcarnitine (C18:1-DC)* | 0.01693 | tartronate (hydroxymalonate) | 0.01848 |
| dihomo-linolenoylcarnitine (C20:3n3 or 6)* | 0.01706 | pyruvate | 0.01864 |
| 3-phenylpropionate (hydrocinnamate) | 0.0175 | saccharin | 0.01871 |
| 2-isopropylmalate | 0.01787 | N-acetylglutamate | 0.0188 |
| 7-HOCA | 0.0179 | eicosenoate (20:1n9 or 1n11) | 0.01886 |
| 3-methyl-2-oxobutyrate | 0.01794 | stachydrine | 0.01895 |
| glutathione, oxidized (GSSG) | 0.0184 | suberoylcarnitine (C8-DC) | 0.01899 |
| 2-hydroxyoctanoate | 0.01848 | N-acetylmethionine | 0.01907 |
| ximenoylcarnitine (C26:1)* | 0.01877 | N-methylpipecolate | 0.01916 |
| 2-hydroxy-3-methylvalerate | 0.01948 | 2-hydroxyglutarate | 0.01919 |
| hippurate | 0.01948 | iminodiacetate (IDA) | 0.01925 |
| behenoylcarnitine (C22)* | 0.01948 | 10-heptadecenoate (17:1n7) | 0.01926 |
| 3-hydroxybutyrate (BHBA) | 0.02029 | N-acetyltyrosine | 0.01932 |
| threonate | 0.02062 | trans-urocanate | 0.01944 |
| 4-hydroxyphenylpyruvate | 0.02069 | phenol sulfate | 0.01947 |
| dimethyl sulfone | 0.02073 | 1-linoleoyl-2-linolenoyl-GPC (18:2/18:3)* | 0.01953 |
| sphingomyelin (d18:1/25:0, d19:0/24:1, d20:1/23:0, d19:1/24:0)* | 0.02073 | argininosuccinate | 0.01963 |
| cysteine | 0.02106 | carboxyethyl-GABA | 0.01965 |
| arachidoylcarnitine (C20)* | 0.02118 | 3-hydroxylaurate | 0.01969 |
| 1-palmitoyl-GPE (16:0) | 0.02129 | oleate/vaccenate (18:1) | 0.02008 |
| (N(1) + N(8))-acetylspermidine | 0.02132 | docosapentaenoate (n6 DPA; 22:5n6) | 0.02017 |
| lanthionine | 0.02172 | 10-nonadecenoate (19:1n9) | 0.02023 |
| stearoyl sphingomyelin (d18:1/18:0) | 0.02208 | 4-vinylphenol sulfate | 0.02027 |
| homocystine | 0.02236 | sphingomyelin (d18:1/20:0, d16:1/22:0)* | 0.02052 |
| N-acetylglucosaminylasparagine | 0.02251 | guaiacol sulfate | 0.02056 |
| N-acetylphenylalanine | 0.02255 | malonylcarnitine | 0.02094 |
| glycerol | 0.02266 | histidine | 0.02114 |
| picolinate | 0.02286 | allantoic acid | 0.02118 |
| deoxycarnitine | 0.02287 | docosadienoate (22:2n6) | 0.0212 |
| benzoate | 0.02323 | 9,10-DiHOME | 0.02153 |
| dodecadienoate (12:2)* | 0.02343 | N-acetylkynurenine (2) | 0.02171 |
| cortisone | 0.02358 | uridine | 0.02171 |
| N-oleoyltaurine | 0.02395 | 1-(1-enyl-palmitoyl)-2-oleoyl-GPC (P-16:0/18:1)* | 0.02173 |
| sphingomyelin (d18:1/20:1, d18:2/20:0)* | 0.02414 | desmosterol | 0.02202 |
| 1-linoleoyl-GPG (18:2)* | 0.02473 | N6-succinyladenosine | 0.02212 |
| azelate (nonanedioate; C9) | 0.0248 | N-methylproline | 0.02212 |
| cis-4-decenoylcarnitine (C10:1) | 0.02508 | 3-hydroxyoctanoate | 0.02253 |
| 2-piperidinone | 0.02529 | 3-hydroxypalmitoylcarnitine | 0.02287 |
| cysteinylglycine | 0.02548 | pentadecanoate (15:0) | 0.0229 |
| margaroylcarnitine (C17)* | 0.02549 | 2-aminoheptanoate | 0.02311 |
| gulonate* | 0.02595 | 1-oleoyl-2-linoleoyl-GPC (18:1/18:2)* | 0.02317 |
| perfluorooctanesulfonic acid (PFOS) | 0.02601 | glycerol | 0.02324 |
| 16-hydroxypalmitate | 0.02608 | trigonelline (N'-methylnicotinate) | 0.0233 |
| 1,2,3-benzenetriol sulfate (2) | 0.02657 | allantoin | 0.02347 |
| glycine | 0.02728 | arachidate (20:0) | 0.02358 |
| indoleacetylcarnitine* | 0.02792 | 5-oxoproline | 0.02374 |
| indoleacetylglutamine | 0.02809 | ribulonate/xylulonate* | 0.02379 |
| malonate | 0.02828 | arabonate/xylonate | 0.02388 |
| 1-(1-enyl-palmitoyl)-2-oleoyl-GPC (P-16:0/18:1)* | 0.02839 | carnitine | 0.02414 |
| 1-methyl-4-imidazoleacetate | 0.0286 | 1-stearoyl-2-linoleoyl-GPC (18:0/18:2)* | 0.02436 |
| linolenoylcarnitine (C18:3)* | 0.02958 | homostachydrine* | 0.02456 |
| erucoylcarnitine (C22:1)* | 0.02958 | 2-hydroxynervonate* | 0.02468 |
| alpha-hydroxyisovalerate | 0.02971 | tetradecanedioate (C14) | 0.02531 |
| lysine | 0.02985 | inosine | 0.02563 |
| 3-acetylphenol sulfate | 0.03009 | stearate (18:0) | 0.02596 |
| N-acetylglutamate | 0.03018 | cytidine | 0.02603 |
| phenylacetylalanine | 0.03028 | campesterol | 0.02619 |
| gamma-glutamylglutamate | 0.0303 | lignoceroyl sphingomyelin (d18:1/24:0) | 0.02624 |
| arachidonoylcarnitine (C20:4) | 0.03039 | 1-(1-enyl-palmitoyl)-2-arachidonoyl-GPC (P-16:0/20:4)* | 0.02639 |
| 4-vinylphenol sulfate | 0.03098 | phenylpyruvate | 0.02641 |
| orotate | 0.03139 | gamma-glutamylcitrulline* | 0.02653 |
| 1-palmitoyl-2-linoleoyl-GPE (16:0/18:2) | 0.03147 | nonadecanoate (19:0) | 0.02684 |
| sebacate (C10-DC) | 0.03174 | butyrylcarnitine (C4) | 0.02687 |
| N-acetylglycine | 0.03179 | 1-palmitoleoyl-2-linoleoyl-GPC (16:1/18:2)* | 0.02702 |
| N-acetylcarnosine | 0.03193 | 1-palmitoleoyl-2-linolenoyl-GPC (16:1/18:3)* | 0.02796 |
| N-acetylputrescine | 0.03195 | N-acetylglutamine | 0.02817 |
| N-acetyltryptophan | 0.03216 | 3-methylglutaconate | 0.0286 |
| 2,3-dihydroxyisovalerate | 0.03272 | 1-methylhypoxanthine | 0.02915 |
| 4-guanidinobutanoate | 0.03272 | 1-(1-enyl-palmitoyl)-2-palmitoyl-GPC (P-16:0/16:0)* | 0.02946 |
| N-acetylisoleucine | 0.03288 | adenosine | 0.02948 |
| 5-methylcytidine | 0.03337 | 1-palmitoyl-2-linoleoyl-GPC (16:0/18:2) | 0.02976 |
| N-delta-acetylornithine | 0.034 | kynurenine | 0.02979 |
| octadecanedioylcarnitine (C18-DC)* | 0.03457 | pimeloylcarnitine/3-methyladipoylcarnitine (C7-DC) | 0.03011 |
| pterin | 0.03481 | 3-hydroxyhippurate | 0.03018 |
| phenol sulfate | 0.03481 | N1-methylinosine | 0.03051 |
| N-acetyl-beta-alanine | 0.03573 | alpha-ketoglutaramate | 0.03121 |
| imidazole lactate | 0.03591 | 3-hydroxyoleoylcarnitine | 0.03143 |
| 1-ribosyl-imidazoleacetate* | 0.03661 | uracil | 0.03157 |
| urate | 0.03662 | N-acetylputrescine | 0.03168 |
| 5-methylthioadenosine (MTA) | 0.03703 | 1-oleoyl-GPE (18:1) | 0.03237 |
| stearoylcarnitine (C18) | 0.03782 | cytosine | 0.03246 |
| cysteinylglycine disulfide* | 0.03787 | arachidonate (20:4n6) | 0.03255 |
| oleoylcarnitine (C18) | 0.03794 | octanoylcarnitine (C8) | 0.03266 |
| linoleoylcarnitine (C18:2)* | 0.03875 | docosapentaenoate (DPA; 22:5n3) | 0.03333 |
| hexanoylglycine (C6) | 0.03913 | linoleoyl ethanolamide | 0.03345 |
| trans-urocanate | 0.03921 | pterin | 0.03429 |
| 1,5-anhydroglucitol (1,5-AG) | 0.03941 | pyridoxal | 0.03432 |
| N1-Methyl-2-pyridone-5-carboxamide | 0.03947 | glycerophosphoinositol* | 0.03439 |
| hydroxyproline | 0.03954 | carotene diol (1) | 0.03483 |
| phenylacetylserine | 0.03977 | guanosine | 0.03485 |
| phenylacetylglutamine | 0.03982 | 3-ureidoisobutyrate | 0.03508 |
| argininosuccinate | 0.03984 | 4-acetamidobutanoate | 0.03583 |
| gamma-glutamylhistidine | 0.04061 | deoxycholate | 0.03635 |
| citrulline | 0.04062 | sphingomyelin (d18:1/25:0, d19:0/24:1, d20:1/23:0, d19:1/24:0)* | 0.03639 |
| 2-oxoarginine* | 0.04064 | 3-methoxytyrosine | 0.03684 |
| ribonate (ribonolactone) | 0.04068 | linolenoylcarnitine (C18:3)* | 0.03691 |
| 7-hydroxyindole sulfate | 0.04113 | 1-arachidonoyl-GPC* (20:4)* | 0.03712 |
| glycerol 3-phosphate | 0.04163 | 3-ureidopropionate | 0.03747 |
| 3beta-hydroxy-5-cholestenoate | 0.04198 | dimethyl sulfone | 0.03794 |
| sulfate* | 0.04215 | isovalerylcarnitine (C5) | 0.03821 |
| butyrylcarnitine (C4) | 0.04231 | succinylcarnitine (C4) | 0.03825 |
| gamma-glutamyltryptophan | 0.04283 | 17-methylstearate (i19:0) | 0.03906 |
| prolylhydroxyproline | 0.04289 | cis-4-decenoylcarnitine (C10:1) | 0.03929 |
| 5-hydroxymethylcytidine | 0.04306 | perfluorooctanesulfonic acid (PFOS) | 0.03956 |
| gamma-glutamylthreonine | 0.04312 | bilirubin (E,E)* | 0.03984 |
| benzoylcarnitine* | 0.04321 | docosahexaenoate (DHA; 22:6n3) | 0.03991 |
| myristoylcarnitine (C14) | 0.04375 | 1-linoleoyl-GPE (18:2)* | 0.04 |
| gamma-glutamylvaline | 0.04378 | bilirubin (E,Z or Z,E)* | 0.04061 |
| N-trimethyl 5-aminovalerate | 0.04425 | bilirubin | 0.04109 |
| mevalonolactone | 0.04433 | gamma-glutamyl-epsilon-lysine | 0.04113 |
| oxalate (ethanedioate) | 0.04435 | hexadecanedioate (C16) | 0.04115 |
| N-acetylvaline | 0.04452 | 1-stearoyl-GPI (18:0) | 0.04129 |
| anserine | 0.04456 | ceramide (d18:1/14:0, d16:1/16:0)* | 0.04139 |
| 2-hydroxybutyrate/2-hydroxyisobutyrate | 0.04505 | behenoyl dihydrosphingomyelin (d18:0/22:0)* | 0.04172 |
| phenylacetate | 0.04542 | beta-sitosterol | 0.04324 |
| 5-oxoproline | 0.04547 | 1-linoleoylglycerol (18:2) | 0.04349 |
| 4-hydroxyphenylacetylglycine | 0.04563 | decanoylcarnitine (C10) | 0.04349 |
| palmitoleoylcarnitine (C16:1)* | 0.04576 | citrulline | 0.04434 |
| nicotinamide riboside | 0.04579 | 3beta-hydroxy-5-cholestenoate | 0.0445 |
| dihomo-linoleoylcarnitine (C20:2)* | 0.04648 | glycosyl ceramide (d18:1/23:1, d17:1/24:1)* | 0.04525 |
| alpha-ketoglutaramate | 0.04648 | N-palmitoylglycine | 0.04525 |
| isovalerylcarnitine (C5) | 0.04656 | 7-methylguanine | 0.04547 |
| N-monomethylarginine | 0.04658 | N-trimethyl 5-aminovalerate | 0.04549 |
| isovalerylglycine | 0.04662 | 1-linolenoyl-GPC (18:3)* | 0.046 |
| indoleacetylglycine | 0.04689 | phenylalanine | 0.04618 |
| N2,N2-dimethylguanine | 0.04706 | myristoylcarnitine (C14) | 0.04695 |
| 3-hydroxylaurate | 0.04718 | palmitoylcarnitine (C16) | 0.04725 |
| palmitoylcarnitine (C16) | 0.04763 | 1-arachidonoyl-GPI* (20:4)* | 0.04739 |
| 6-oxopiperidine-2-carboxylate | 0.04766 | lignoceroylcarnitine (C24)* | 0.04784 |
| pipecolate | 0.04856 | oleoyl ethanolamide | 0.04816 |
| malonylcarnitine | 0.04857 | 1-stearoyl-GPC (18:0) | 0.0482 |
| caproate (6:0) | 0.04868 | glycerol 3-phosphate | 0.04858 |
| 1-(1-enyl-palmitoyl)-2-palmitoyl-GPC (P-16:0/16:0)* | 0.04868 | hexanoylcarnitine (C6) | 0.04861 |
| gamma-glutamylphenylalanine | 0.04868 | stearoyl sphingomyelin (d18:1/18:0) | 0.04863 |
| kynurenate | 0.04878 | arachidonoylcholine | 0.04877 |
| laurylcarnitine (C12) | 0.04926 | 1-lignoceroyl-GPC (24:0) | 0.04878 |
| 2-aminoadipate | 0.04966 | 1-oleoyl-GPI (18:1)* | 0.0499 |
| argininate* | 0.04966 | 1-palmitoleoyl-GPC* (16:1)* | 0.05026 |
| dodecanedioate (C12) | 0.04972 | sphingomyelin (d18:0/18:0, d19:0/17:0)* | 0.0504 |
| myristoleoylcarnitine (C14:1)* | 0.04976 | alpha-tocopherol | 0.05098 |
| 3-hydroxyoctanoate | 0.05019 | 2'-deoxyuridine | 0.05125 |
| 5-dodecenoylcarnitine (C12:1) | 0.05021 | tricosanoyl sphingomyelin (d18:1/23:0)* | 0.05179 |
| N-palmitoleoyltaurine* | 0.05044 | 1-palmitoyl-GPI* (16:0) | 0.05197 |
| S-adenosylhomocysteine (SAH) | 0.0505 | 1-linoleoyl-2-arachidonoyl-GPC (18:2/20:4n6)* | 0.0524 |
| propionylcarnitine (C3) | 0.05117 | eicosenoylcarnitine (C20:1)* | 0.05255 |
| 3-hydroxyindolin-2-one sulfate | 0.05177 | laurylcarnitine (C12) | 0.05307 |
| homostachydrine* | 0.05181 | hexadecenedioate (C16:1-DC)* | 0.05354 |
| 1-methyladenosine | 0.05254 | adrenoylcarnitine (C22:4)* | 0.05402 |
| N-acetylalanine | 0.05318 | 5-methyl-2'-deoxycytidine | 0.05403 |
| riboflavin (Vitamin B2) | 0.0533 | 1,2-dilinoleoyl-GPC (18:2/18:2) | 0.05405 |
| N-acetyltaurine | 0.05359 | cerotoylcarnitine (C26)* | 0.05427 |
| arabonate/xylonate | 0.05359 | arachidonoylcarnitine (C20:4) | 0.05466 |
| gamma-glutamylglutamine | 0.05431 | palmitoyl dihydrosphingomyelin (d18:0/16:0)* | 0.05469 |
| carnitine | 0.05436 | sphingomyelin (d18:0/20:0, d16:0/22:0)* | 0.0547 |
| retinol (Vitamin A) | 0.05458 | myristoyl dihydrosphingomyelin (d18:0/14:0)* | 0.05503 |
| 4-hydroxyphenylacetate sulfate | 0.05517 | N-stearoylserine* | 0.05524 |
| 3-hydroxydecanoate | 0.05526 | 5-dodecenoylcarnitine (C12:1) | 0.05591 |
| 4-methylcatechol sulfate | 0.05572 | 2-hydroxypalmitate | 0.05661 |
| valerate (5:0) | 0.05627 | sphingomyelin (d18:1/21:0, d17:1/22:0, d16:1/23:0)* | 0.0568 |
| N-acetylglutamine | 0.05667 | sphingomyelin (d18:2/24:1, d18:1/24:2)* | 0.05704 |
| isovalerate (C5) | 0.0572 | 2'-O-methyluridine | 0.05753 |
| gamma-glutamyl-epsilon-lysine | 0.05727 | glycerophosphorylcholine (GPC) | 0.05772 |
| 2-methylbutyroylcarnitine (C5) | 0.05729 | 1-(1-enyl-palmitoyl)-2-linoleoyl-GPC (P-16:0/18:2)* | 0.05784 |
| carnosine | 0.05798 | ceramide (d16:1/24:1, d18:1/22:1)* | 0.05804 |
| mevalonate | 0.058 | 2-methylbutyroylcarnitine (C5) | 0.0582 |
| N-methylpipecolate | 0.05803 | urate | 0.05843 |
| N4-acetylcytidine | 0.05858 | sphingomyelin (d18:1/24:1, d18:2/24:0)* | 0.05846 |
| pyroglutamine* | 0.05883 | N-palmitoyl-sphingosine (d18:1/16:0) | 0.05872 |
| N-formylmethionine | 0.05888 | stearoylcarnitine (C18) | 0.05881 |
| N-acetylmethionine | 0.05945 | 2'-O-methylcytidine | 0.05892 |
| 5-hydroxyindole sulfate | 0.0597 | palmitoleoylcarnitine (C16:1)* | 0.05897 |
| 6-hydroxyindole sulfate | 0.05976 | nervonoylcarnitine (C24:1)* | 0.05907 |
| decanoylcarnitine (C10) | 0.05984 | xanthine | 0.05996 |
| imidazole propionate | 0.05997 | sphingomyelin (d18:1/18:1, d18:2/18:0) | 0.05997 |
| 3-hydroxyhexanoate | 0.0609 | 2'-deoxycytidine | 0.06011 |
| adrenoylcarnitine (C22:4)* | 0.06162 | 1-palmitoyl-GPG (16:0)* | 0.06021 |
| 2-oxindole-3-acetate | 0.06238 | 5-hydroxymethyl-2'-deoxycytidine | 0.06022 |
| gamma-glutamylcitrulline* | 0.06292 | myristoleoylcarnitine (C14:1)* | 0.06098 |
| suberate (C8-DC) | 0.06326 | N-stearoyl-sphingosine (d18:1/18:0)* | 0.06117 |
| homocitrulline | 0.06331 | taurodeoxycholate | 0.06142 |
| gamma-glutamylleucine | 0.06353 | nervonate (24:1n9)* | 0.06161 |
| ribulonate/xylulonate* | 0.06365 | N-behenoyl-sphingadienine (d18:2/22:0)* | 0.06205 |
| aconitate [cis or trans] | 0.06426 | N-palmitoyl-sphingadienine (d18:2/16:0)* | 0.06214 |
| eicosenoylcarnitine (C20:1)* | 0.06503 | sphingomyelin (d18:1/20:1, d18:2/20:0)* | 0.06232 |
| 3-hydroxypalmitoylcarnitine | 0.06596 | eicosapentaenoate (EPA; 20:5n3) | 0.06277 |
| 3-indoxyl sulfate | 0.0664 | 1-stearoyl-2-oleoyl-GPC (18:0/18:1) | 0.06299 |
| glutaroylcarnitine (C5) | 0.06739 | N2,N2-dimethylguanosine | 0.06365 |
| ophthalmate | 0.06744 | 1-(1-enyl-palmitoyl)-GPC (P-16:0)* | 0.06386 |
| gamma-glutamylisoleucine* | 0.06776 | 2-palmitoyl-GPC* (16:0)* | 0.06404 |
| citrate | 0.06868 | sphingomyelin (d18:1/14:0, d16:1/16:0)* | 0.06419 |
| N-acetylfelinine* | 0.06898 | margaroylcarnitine (C17)* | 0.06475 |
| trimethylamine N-oxide | 0.06936 | 2-hydroxystearate | 0.06484 |
| N6-acetyllysine | 0.06953 | ceramide (d18:2/24:1, d18:1/24:2)* | 0.06491 |
| phenylacetylcarnitine | 0.06987 | 5-methylcytidine | 0.06533 |
| N-methylproline | 0.06996 | 1-linoleoyl-GPI* (18:2)* | 0.06549 |
| 3-hydroxyoleoylcarnitine | 0.06998 | stearoylcholine* | 0.06621 |
| myo-inositol | 0.07138 | retinal | 0.06622 |
| N6-succinyladenosine | 0.07141 | 1-palmitoyl-GPC (16:0) | 0.06638 |
| 3-hydroxyisobutyrate | 0.07157 | linoleoylcarnitine (C18:2)* | 0.06655 |
| 3-hydroxyphenylacetate sulfate | 0.07253 | ceramide (d18:1/17:0, d17:1/18:0)* | 0.06669 |
| N-acetyltyrosine | 0.07264 | 1-oleoylglycerol (18:1) | 0.0667 |
| hexanoylcarnitine (C6) | 0.07319 | ximenoylcarnitine (C26:1)* | 0.06679 |
| dimethylarginine (ADMA + SDMA) | 0.0733 | behenoyl sphingomyelin (d18:1/22:0)* | 0.06757 |
| 4-acetamidobutanoate | 0.07364 | arachidoylcarnitine (C20)* | 0.06841 |
| N-acetylserine | 0.07383 | sphingomyelin (d18:2/14:0, d18:1/14:1)* | 0.06889 |
| arabitol/xylitol | 0.07437 | dihomo-linoleoylcarnitine (C20:2)* | 0.06966 |
| octanoylcarnitine (C8) | 0.07445 | sphingomyelin (d18:2/23:0, d18:1/23:1, d17:1/24:1)* | 0.07033 |
| 5-hydroxyindoleacetate | 0.07447 | sphingomyelin (d18:2/21:0, d16:2/23:0)* | 0.0708 |
| 3-methylhistidine | 0.07574 | glycosyl ceramide (d18:2/24:1, d18:1/24:2)* | 0.071 |
| stachydrine | 0.07601 | ceramide (d18:1/20:0, d16:1/22:0, d20:1/18:0)* | 0.07173 |
| pimeloylcarnitine/3-methyladipoylcarnitine (C7-DC) | 0.07642 | oleoylcarnitine (C18) | 0.07276 |
| 1-methylhistidine | 0.077 | sphingomyelin (d17:1/14:0, d16:1/15:0)* | 0.07315 |
| beta-hydroxyisovalerate | 0.07757 | dihomo-linolenoylcarnitine (C20:3n3 or 6)* | 0.0732 |
| 5-(galactosylhydroxy)-L-lysine | 0.07757 | 4-cholesten-3-one | 0.07325 |
| p-cresol sulfate | 0.07776 | sphingomyelin (d18:2/24:2)* | 0.07356 |
| orotidine | 0.07827 | 1-stearoyl-GPG (18:0) | 0.07374 |
| ectoine | 0.07863 | sphingomyelin (d17:2/16:0, d18:2/15:0)* | 0.07483 |
| isobutyrylglycine (C4) | 0.07899 | N-oleoylserine | 0.07533 |
| succinylcarnitine (C4) | 0.0792 | kynurenate | 0.07565 |
| methionine sulfone | 0.08 | linoleoylcholine* | 0.07588 |
| N-acetylarginine | 0.08005 | glycosyl-N-stearoyl-sphinganine (d18:0/18:0)* | 0.07655 |
| N1-methylinosine | 0.08032 | 1-linoleoyl-GPC (18:2) | 0.07708 |
| gamma-glutamylglycine | 0.08105 | palmitoylcholine | 0.07765 |
| azeloylcarnitine (C9-DC) | 0.08154 | taurolithocholate 3-sulfate | 0.07793 |
| 3-hydroxy-2-ethylpropionate | 0.08175 | taurocholate | 0.07796 |
| phenylacetylglycine | 0.08204 | taurochenodeoxycholate | 0.07803 |
| guanidinosuccinate | 0.08252 | sphingomyelin (d18:2/16:0, d18:1/16:1)* | 0.07863 |
| 1-methylhypoxanthine | 0.08287 | erucoylcarnitine (C22:1)* | 0.07867 |
| creatinine | 0.08308 | gulonate* | 0.07887 |
| allantoic acid | 0.08338 | taurocholenate sulfate | 0.0792 |
| tiglyl carnitine (C5) | 0.08483 | formiminoglutamate | 0.07934 |
| 3-hydroxybutyrylcarnitine (2) | 0.08487 | docosapentaenoylcarnitine (C22:5n3)* | 0.07966 |
| allantoin | 0.08494 | 1-linoleoyl-GPG (18:2)* | 0.0801 |
| pseudouridine | 0.08529 | sphingomyelin (d18:1/22:1, d18:2/22:0, d16:1/24:1)* | 0.08063 |
| isobutyrylcarnitine (C4) | 0.08567 | 7-HOCA | 0.08116 |
| 3-methylglutaconate | 0.08575 | glycosyl-N-behenoyl-sphingadienine (d18:2/22:0)* | 0.08203 |
| erythronate* | 0.08596 | oleoylcholine | 0.08246 |
| trigonelline (N'-methylnicotinate) | 0.08621 | 1-palmitoyl-2-oleoyl-GPC (16:0/18:1) | 0.08257 |
| N6,N6,N6-trimethyllysine | 0.08625 | alpha-CEHC sulfate | 0.08287 |
| O-sulfo-L-tyrosine | 0.08631 | cholesterol | 0.08301 |
| pyridoxate | 0.08648 | sphingomyelin (d18:1/22:2, d18:2/22:1, d16:1/24:2)* | 0.08315 |
| N-acetylthreonine | 0.08674 | 1-oleoyl-GPC (18:1) | 0.08372 |
| 2-methylmalonylcarnitine (C4-DC) | 0.0869 | sphingomyelin (d18:2/18:1)* | 0.08423 |
| 3-hydroxy-3-methylglutarate | 0.087 | glycosyl-N-(2-hydroxynervonoyl)-sphingosine (d18:1/24:1(2OH))* | 0.08513 |
| hydroxyasparagine | 0.08772 | octadecanedioylcarnitine (C18-DC)* | 0.08536 |
| phenylacetylglutamate | 0.08782 | sphingomyelin (d18:1/17:0, d17:1/18:0, d19:1/16:0) | 0.08646 |
| N-acetyl-isoputreanine* | 0.08806 | octadecenedioylcarnitine (C18:1-DC)* | 0.08652 |
| C-glycosyltryptophan | 0.08832 | sphingomyelin (d17:1/16:0, d18:1/15:0, d16:1/17:0)* | 0.08782 |
| acetylcarnitine (C2) | 0.08871 | glucuronate | 0.08804 |
| adipoylcarnitine (C6-DC) | 0.08915 | glycosyl ceramide (d16:1/24:1, d18:1/22:1)* | 0.08868 |
| 7-methylguanine | 0.08932 | sphingomyelin (d18:2/23:1)* | 0.08904 |
| erythritol | 0.08952 | palmitoyl sphingomyelin (d18:1/16:0) | 0.0894 |
| suberoylcarnitine (C8-DC) | 0.08966 | glycosyl ceramide (d18:1/20:0, d16:1/22:0)* | 0.08989 |
| 1-methylguanidine | 0.09168 | glycosyl-N-palmitoyl-sphingosine (d18:1/16:0) | 0.09026 |
| N6-carbamoylthreonyladenosine | 0.09171 | sphingomyelin (d18:1/20:2, d18:2/20:1, d16:1/22:2)* | 0.09181 |
| urea | 0.09202 | glycosyl-N-stearoyl-sphingosine (d18:1/18:0) | 0.09206 |
| 5,6-dihydrouridine | 0.09493 | sphingomyelin (d18:1/19:0, d19:1/18:0)* | 0.09452 |
