# Supplementary material for: Chronic kidney disease in cats alters response of the plasma metabolome and fecal microbiome to dietary fiber
Source: PLoS One. 2020 Jul 2;15(7):e0235480. doi: 10.1371/journal.pone.0235480 (PMC7331996; doi:10.1371/journal.pone.0235480)
Supplement: S4 Table — (DOCX) [file pone.0235480.s004.docx]

**Table S4.** Changes in the abundance of bacterial operational taxonomic units (OTUs) using centered log-ratio (CLR) transformed microbiome data for a) differences between CKD cats and healthy cats at baseline, b) CKD cats only: differences in OTUs from baseline after consuming food containing scFOS, c) CKD cats only: differences in OTUs from baseline after consuming food containing apple pomace, d) CKD cats only: differences in OTUs after consuming food containing apple pomace versus food containing scFOS, e) Healthy cats only: differences in OTUs from baseline after consuming food containing scFOS, f) Healthy cats only: differences in OTUs from baseline after consuming food containing apple pomace, and g) Healthy cats only: differences in OTUs after consuming food containing apple pomace versus food containing scFOS.

|  | **OTUs** | **Mean difference** | **SE** | **P-value** |
| --- | --- | --- | --- | --- |
| **a) Baseline:**  **CKD-HC** | 100296__o__Bacteroidales;_f__S24-7;_g_ | -1.19 | 0.3 | 0.001 |
|  | 1003206__g__Sphingomonas_unclassified | 1.77 | 0.54 | 0.005 |
|  | 100883__ f__Legionellaceae_unclassified | -0.71 | 0.24 | 0.013 |
|  | 102407_g__Bacteroides;_s__ | -3.28 | 1.2 | 0.023 |
|  | 1103499_ _g__Peptoniphilus;_s__ | -0.44 | 0.18 | 0.025 |
| **b) CKD cats only:**  **scFOS-BSL** | 1000113_ o__Clostridiales_unclassified | -0.46 | 0.08 | 0.0004 |
|  | 1024529__f__Clostridiaceae;_g_ | -0.63 | 0.13 | 0.0008 |
|  | 99508__g__Turicibacter;_ | -1.05 | 0.23 | 0.0014 |
|  | 4319416_g__Bartonella;_s__bacilliformis | -0.51 | 0.12 | 0.0016 |
|  | 100212_ f__Veillonellaceae;_g__; | -2.96 | 0.76 | 0.0036 |
|  | 305561_ g__Enterococcus_unclassified | -1.51 | 0.40 | 0.0041 |
|  | 856920_g__Gallicola | -1.03 | 0.28 | 0.0049 |
|  | 100296_ o__Bacteroidales;_f__S24-7;_g__ | 0.51 | 0.14 | 0.0056 |
|  | 893335_ f__Streptococcaceae_unclassified | -0.49 | 0.15 | 0.0086 |
|  | 206978_ g__Methanosarcina;_s__mazei | -1.01 | 0.31 | 0.0099 |
|  | 4441081_f__Coriobacteriaceae;_g_ | 1.01 | 0.31 | 0.0101 |
|  | 839684_ f__Lachnospiraceae_unclassified | -0.58 | 0.19 | 0.0146 |
|  | 1067143_f__Rikenellaceae;_g_ | 0.52 | 0.17 | 0.0150 |
|  | 100001_c__Deltaproteobacteria_unclassified | -1.01 | 0.35 | 0.0171 |
|  | 2530636_g__Megamonas | -0.97 | 0.36 | 0.0243 |
|  | 4361046_g__Campylobacter_unclassified | 2.14 | 0.83 | 0.0300 |
|  | 28914_g__Roseburia | 1.36 | 0.54 | 0.0322 |
|  | 16204_ g__Sarcina | 0.72 | 0.29 | 0.0346 |
|  | 651436_ g__Ralstonia | 0.40 | 0.16 | 0.0370 |
|  | 266392__g__Coprococcus | 0.65 | 0.27 | 0.0393 |
|  | 109554_ g__Epulopiscium | 0.44 | 0.18 | 0.0422 |
|  | 1000592_ g__Anaerococcus | -0.55 | 0.23 | 0.0437 |
| **c) CKD cats only:**  **Apple pomace - BSL** | 1024529___ f__Clostridiaceae;_g_ | -1.51 | 0.20 | 0.0001 |
|  | 128382__g__Dialister | -1.06 | 0.15 | 0.0001 |
|  | 302647__g__Collinsella | -0.68 | 0.10 | 0.0002 |
|  | 893335_f__Streptococcaceae_unclassified | -0.61 | 0.14 | 0.0027 |
|  | 856920_g__Gallicola | -1.18 | 0.30 | 0.0040 |
|  | 839684_f_Lachnospiraceae_unclassified | -0.76 | 0.19 | 0.0044 |
|  | 4319416_ g__Bartonella;_s__bacilliformis | -0.66 | 0.17 | 0.0048 |
|  | 1000113_ o__Clostridiales_unclassified | -0.51 | 0.13 | 0.0050 |
|  | 99508_g__Turicibacter | -1.03 | 0.27 | 0.0053 |
|  | 510683_ g__Desulfovibrio | -0.51 | 0.13 | 0.0055 |
|  | 110221_g__Ruminococcus;_s__gnavus | -0.54 | 0.15 | 0.0064 |
|  | 1084643_g__Mogibacterium;_s_ | -0.44 | 0.13 | 0.0081 |
|  | 384716_o__Gemellales;_f__;_g_ | 1.10 | 0.33 | 0.0105 |
|  | 100212_f__Veillonellaceae;_g_ | -2.15 | 0.71 | 0.0167 |
|  | 1099710_ g__Peptococcus | -0.47 | 0.16 | 0.0172 |
|  | 100744_g__Butyrivibrio | 0.70 | 0.28 | 0.0362 |
|  | 1120090_ g__Leucobacter | -1.44 | 0.62 | 0.0485 |
| **d) CKD cats only:**  **Apple pomace - scFOS** | 1024529_f__Clostridiaceae;_g_ | -0.85 | 0.28 | 0.0176 |
|  | 100035_f__Helicobacteraceae_unclassified | -0.76 | 0.29 | 0.0306 |
|  | 305561_g__Enterococcus_unclassified | 0.61 | 0.23 | 0.0309 |
|  | 28914__g__Roseburia | -0.76 | 0.30 | 0.0357 |
|  | 356403_g__Clostridium | -0.49 | 0.20 | 0.0380 |
|  | 527323_g__Yersinia | 1.08 | 0.43 | 0.0381 |
|  | 1000161_f__Pseudomonadaceae_unclassified | -1.15 | 0.49 | 0.0450 |
| **e) Healthy Cats only:**  **scFOS-BSL** | 99508__g__Turicibacter;_s_ | -1.22 | 0.15 | 2E-07 |
|  | 206978__g__Methanosarcina;_s__mazei | -1.20 | 0.21 | 1E-05 |
|  | 100212_f__Veillonellaceae;_g__;_s_ | -2.85 | 0.60 | 1E-04 |
|  | 308309_f__Peptostreptococcaceae;_g__;_s_ | -1.62 | 0.35 | 2E-04 |
|  | 893335_f__Streptococcaceae_unclassified | -0.43 | 0.10 | 3E-04 |
|  | 856920_g__Gallicola;_s_ | -0.82 | 0.19 | 0.0005 |
|  | 651436_g__Ralstonia;_s_ | 0.33 | 0.09 | 0.0015 |
|  | 305561_g__Enterococcus_unclassified | -1.07 | 0.30 | 0.0018 |
|  | 4441081_f__Coriobacteriaceae;_g__;_s_ | 1.00 | 0.28 | 0.0018 |
|  | 1066621_f__Prevotellaceae_unclassified | 0.22 | 0.06 | 0.0020 |
|  | 1030652_g__Odoribacter;_s_ | 0.21 | 0.06 | 0.0020 |
|  | 109554_g__Epulopiscium;_s_ | 0.35 | 0.10 | 0.0024 |
|  | 1103170_g__Parvimonas;_s_ | 0.45 | 0.13 | 0.0032 |
|  | 1067143_f__Rikenellaceae;_g__;_s_ | 0.34 | 0.10 | 0.0036 |
|  | 1000113_o__Clostridiales_unclassified | -0.40 | 0.12 | 0.0037 |
|  | 98948_f__Ruminococcaceae_unclassified | -0.63 | 0.19 | 0.0041 |
|  | 4384058_g__Salmonella_unclassified | 0.69 | 0.22 | 0.0047 |
|  | 1120090_g__Leucobacter;_s_ | -1.16 | 0.37 | 0.0054 |
|  | 1007180_g__Methylobacterium;_s_ | 0.18 | 0.06 | 0.0065 |
|  | 302647__g__Collinsella;_s_ | -0.40 | 0.13 | 0.0072 |
|  | 16204_g__Sarcina;_s_ | 0.49 | 0.16 | 0.0073 |
|  | 652696_g__Bifidobacterium;_s__adolescentis | 0.85 | 0.29 | 0.0093 |
|  | 135410_g__Anaerofustis;_s_ | 0.16 | 0.05 | 0.0097 |
|  | 510683_g__Desulfovibrio;_s_ | -0.34 | 0.12 | 0.0103 |
|  | 1000592_g__Anaerococcus;_s_ | -0.57 | 0.20 | 0.0106 |
|  | 4319416_g__Bartonella;_s__bacilliformis | -0.62 | 0.22 | 0.0107 |
|  | 298592_f__Fusobacteriaceae;_g__;_s_ | 0.18 | 0.06 | 0.0110 |
|  | 11299_f__Leptotrichiaceae;_g__;_s_ | 0.25 | 0.09 | 0.0115 |
|  | 101021_f__Succinivibrionaceae_unclassified | 0.86 | 0.32 | 0.0149 |
|  | 384716_o__Gemellales;_f__;_g__;_s_ | 0.90 | 0.34 | 0.0169 |
|  | 1038384_g__Acidaminococcus;_s_ | 0.39 | 0.15 | 0.0184 |
|  | 104272_f__Mogibacteriaceae_unclassified | -0.64 | 0.25 | 0.0195 |
|  | 40839_g__Catenibacterium;_s_ | 0.56 | 0.23 | 0.0250 |
|  | 146880_g__Bulleidia;_s__p-1630-c5 | 0.21 | 0.09 | 0.0267 |
|  | 4361046__g__Campylobacter_unclassified | 1.19 | 0.50 | 0.0270 |
|  | 1017249_f__Bifidobacteriaceae;_g__;_s_ | -0.52 | 0.22 | 0.0287 |
|  | 1001762_f__Streptococcaceae;_g__;_s_ | 0.13 | 0.06 | 0.0296 |
|  | 128382_g__Dialister;_s_ | -0.33 | 0.14 | 0.0313 |
|  | 361186_g__Blautia;_s_ | 0.71 | 0.32 | 0.0381 |
|  | 4379318_f__Pseudomonadaceae;_g__;_s_ | 0.44 | 0.20 | 0.0381 |
|  | 1057116_p__Bacteroidetes_unclassified | 0.36 | 0.16 | 0.0392 |
| **f) Healthy Cats only:**  **Apple pomace - BSL** | 99508_g__Turicibacter;_s__ | -1.29 | 0.19 | 0.0001 |
|  | 98948_f__Ruminococcaceae_unclassified | -0.99 | 0.15 | 0.0001 |
|  | 52166_g__Megasphaera;_s__ | 0.72 | 0.11 | 0.0001 |
|  | 135410_g__Anaerofustis;_s__ | 0.34 | 0.06 | 0.0002 |
|  | 1066621_f__Prevotellaceae_unclassified | 0.34 | 0.06 | 0.0002 |
|  | 651436_g__Ralstonia;_s__ | 0.34 | 0.06 | 0.0002 |
|  | 1007180_g__Methylobacterium;_s__ | 0.34 | 0.06 | 0.0002 |
|  | 401717___k__Bacteria;_p__TM7;_c__TM7-3;_o__CW040;_f__F16;_g__;_s__ | 0.37 | 0.07 | 0.0006 |
|  | 1001762_f__Streptococcaceae;_g__;_s__ | 0.37 | 0.07 | 0.0007 |
|  | 101844___k__Bacteria;_p__TM7;_c__TM7-3;_o__;_f__;_g__;_s__ | 0.37 | 0.07 | 0.0007 |
|  | 1084045_g__Lysinibacillus;_s__ | 0.63 | 0.13 | 0.0009 |
|  | 100065_f__Peptococcaceae_unclassified | 0.50 | 0.11 | 0.0012 |
|  | 100744_g__Butyrivibrio;_s__ | 0.32 | 0.07 | 0.0013 |
|  | 1000062_o__Bacteroidales_unclassified | 0.35 | 0.08 | 0.0016 |
|  | 163857_g__Succinivibrio;_s__ | 0.29 | 0.07 | 0.0020 |
|  | 1057116_p__Bacteroidetes_unclassified | 0.66 | 0.15 | 0.0020 |
|  | 104272_f__Mogibacteriaceae_unclassified | -1.38 | 0.32 | 0.0021 |
|  | 1103170_g__Parvimonas;_s__ | 0.47 | 0.11 | 0.0024 |
|  | 206978__g__Methanosarcina;_s__mazei | -1.18 | 0.28 | 0.0025 |
|  | 308309_f__Peptostreptococcaceae;_g__;_s__ | -2.16 | 0.54 | 0.0032 |
|  | 128382_g__Dialister;_s__ | -1.00 | 0.26 | 0.0036 |
|  | 249375__p__Actinobacteria_unclassified | -1.87 | 0.49 | 0.0041 |
|  | 1024529_f__Clostridiaceae;_g__;_s__ | -1.19 | 0.33 | 0.0058 |
|  | 1006387_f__Thermoactinomycetaceae_unclassified | 0.51 | 0.15 | 0.0065 |
|  | 1120090_g__Leucobacter;_s__ | -1.48 | 0.42 | 0.0068 |
|  | 261251_f__Clostridiaceae_unclassified | -1.03 | 0.30 | 0.0071 |
|  | 302647_g__Collinsella;_s__ | -0.36 | 0.10 | 0.0077 |
|  | 1122547_f__Bacillaceae;_g__;_s__ | 0.46 | 0.14 | 0.0088 |
|  | 1030652_g__Odoribacter;_s__ | 0.29 | 0.09 | 0.0092 |
|  | 298592_f__Fusobacteriaceae;_g__;_s__ | 0.29 | 0.09 | 0.0092 |
|  | 109554_g__Epulopiscium;_s__ | 0.48 | 0.16 | 0.0128 |
|  | 1000113_o__Clostridiales_unclassified | -0.59 | 0.19 | 0.0138 |
|  | 4384058_g__Salmonella_unclassified | 1.03 | 0.35 | 0.0153 |
|  | 1000161_f__Pseudomonadaceae_unclassified | -1.31 | 0.45 | 0.0172 |
|  | 1000735_g__Corynebacterium_unclassified | -0.95 | 0.33 | 0.0173 |
|  | 367139_g__Slackia;_s__ | -0.92 | 0.32 | 0.0174 |
|  | 1124879_g__Gallibacterium;_s__ | 0.26 | 0.09 | 0.0177 |
|  | 146880_g__Bulleidia;_s__p-1630-c5 | 0.45 | 0.16 | 0.0180 |
|  | 1009482_g__Legionella;_s__ | 0.26 | 0.09 | 0.0180 |
|  | 652696_g__Bifidobacterium;_s__adolescentis | 1.06 | 0.38 | 0.0209 |
|  | 40839_g__Catenibacterium;_s__ | 0.69 | 0.25 | 0.0210 |
|  | 4435655_c__Alphaproteobacteria;_o__RF32;_f_g_ | 0.47 | 0.18 | 0.0276 |
|  | 146665_g__Eubacterium;_s__biforme | -1.28 | 0.49 | 0.0291 |
|  | 4441081_f__Coriobacteriaceae;_g__;_s__ | 1.05 | 0.45 | 0.0438 |
|  | 1038384_g__Acidaminococcus;_s__ | 0.61 | 0.26 | 0.0457 |
|  | 100035_f__Helicobacteraceae_unclassified | -0.92 | 0.40 | 0.0476 |
| **g) Healthy Cats only:**  **Apple pomace - scFOS** | 1024529_f__Clostridiaceae;_g__;_s_ | -1.05 | 0.24 | 0.0004 |
|  | 1000161_f__Pseudomonadaceae_unclassified | -1.12 | 0.27 | 0.0006 |
|  | 128382_g__Dialister;_s_ | -0.68 | 0.17 | 0.0008 |
|  | 4410166_g__Prevotella;_s__copri | 0.65 | 0.18 | 0.0019 |
|  | 1000735_g__Corynebacterium_unclassified | -0.95 | 0.27 | 0.0028 |
|  | 100035_f__Helicobacteraceae_unclassified | -0.59 | 0.18 | 0.0048 |
|  | 28914_g__Roseburia;_s_ | -0.94 | 0.31 | 0.0075 |
|  | 529762_g__Mycobacterium_unclassified | -0.59 | 0.21 | 0.0127 |
|  | 1017249_f__Bifidobacteriaceae;_g__;_s_ | 0.35 | 0.13 | 0.0164 |
|  | 100212_f__Veillonellaceae;_g__;_s_ | 1.10 | 0.42 | 0.0184 |
|  | 261251_f__Clostridiaceae_unclassified | -0.50 | 0.20 | 0.0209 |
|  | 100001_c__Deltaproteobacteria_unclassified | 0.62 | 0.26 | 0.0298 |
|  | 1099710_g__Peptococcus;_s_ | -0.35 | 0.15 | 0.0306 |
|  | 195865_f__Lachnospiraceae;_g__;_s_ | -0.97 | 0.43 | 0.0351 |
|  | 1057116_p__Bacteroidetes_unclassified | 0.20 | 0.09 | 0.0375 |
|  | 530416_g__Acinetobacter;_s__lwoffii | -0.32 | 0.14 | 0.0392 |
|  | 527323_g__Yersinia;_s_ | 0.53 | 0.25 | 0.0473 |
